# LncRNA XIST regulates breast cancer stem cells by activating proinflammatory IL-6/STAT3 signaling

**DOI:** 10.1038/s41388-023-02652-3

**Published:** 2023-03-15

**Authors:** Yuxi Ma, Yongyou Zhu, Li Shang, Yan Qiu, Na Shen, Jonathan Wang, Tiffany Adam, Wei Wei, Qingxuan Song, Jun Li, Max S. Wicha, Ming Luo

**Affiliations:** 1 Department of Internal Medicine, Division of Hematology and Oncology, University of Michigan Medical School, Ann Arbor, MI 48109 USA; 2 University of Michigan Rogel Cancer Center, Ann Arbor, MI 48109 USA; 3 Department of Breast and Thyroid Surgery, Peking University Shenzhen Hospital, Shenzhen, 518036 China; 4 Department of Human Genetics, University of Michigan Medical School, Ann Arbor, MI 48109 USA; 5 Present Address: Department of Cancer Center, Union Hospital, Tongji Medical College, Huazhong University of Science and Technology, Wuhan, 430022 China; 6 Present Address: Department of Breast and Thyroid Surgery, Union Hospital, Tongji Medical College, Huazhong University of Science and Technology, Wuhan, 430022 China

**Keywords:** Cancer stem cells, Non-coding RNAs

## Abstract

Aberrant expression of XIST, a long noncoding RNA (lncRNA) initiating X chromosome inactivation (XCI) in early embryogenesis, is a common feature of breast cancer (BC). However, the roles of post-XCI XIST in breast carcinogenesis remain elusive. Here we identify XIST as a key regulator of breast cancer stem cells (CSCs), which exhibit aldehyde dehydrogenase positive (ALDH^+^) epithelial- (E) and CD24^lo^CD44^hi^ mesenchymal-like (M) phenotypes. *XIST* is variably expressed across the spectrum of BC subtypes, and doxycycline (DOX)-inducible knockdown (KD) of *XIST* markedly inhibits spheroid/colony forming capacity, tumor growth and tumor-initiating potential. This phenotype is attributed to impaired E-CSC in luminal and E- and M-CSC activities in triple-negative (TN) BC. Gene expression profiling unveils that *XIST* KD most significantly affects cytokine-cytokine receptor interactions, leading to markedly suppressed expression of proinflammatory cytokines IL-6 and IL-8 in ALDH^-^ bulk BC cells. Exogenous IL-6, but not IL-8, rescues the reduced sphere-forming capacity and proportion of ALDH^+^ E-CSCs in luminal and TN BC upon *XIST* KD. XIST functions as a nuclear sponge for microRNA let-7a-2-3p to activate IL-6 production from ALDH^-^ bulk BC cells, which acts in a paracrine fashion on ALDH^+^ E-CSCs that display elevated cell surface IL-6 receptor (IL6R) expression. This promotes CSC self-renewal via STAT3 activation and expression of key CSC factors including c-MYC, KLF4 and SOX9. Together, this study supports a novel role of XIST by derepressing let-7 controlled paracrine IL-6 proinflammatory signaling to promote CSC self-renewal.

## Introduction

Cancer-stem like cells (CSCs), also called tumor initiating cells (TICs), promote tumorigenesis, disease progression, therapeutic resistance, and metastasis [1, 2]. In breast cancer (BC) and other malignancies, elevated ALDH activity is widely used to identify a highly tumorigenic cell population with the capacities of self-renewal and differentiation, driving primary tumor growth and distant metastases [3–8]. Distinct from more quiescent basal/mesenchymal CSCs characterized by CD24^-/lo^CD44^+/hi^ marker expression [9], CSCs characterized by high ALDH activity display an epithelial-like (E), proliferative phenotype [10]. These ALDH^+^ CSCs, designed as E-CSCs, express high level of phosphorylated STAT3 with nuclear localization [11], suggesting a critical role of STAT3 signaling in maintaining this proliferative CSC population.

Tumor cells and their microenvironment co-evolve to drive tumor growth and progression [12]. Paracrine signaling between distinct subsets of tumor cells and between tumor cells and stromal cells constantly modulates tumorigenic CSCs [13, 14]. Proinflammatory cytokines, such as IL-1, IL-6, and IL-8, play critical roles in the induction and maintenance of CSCs by activating STAT3/NFκB signaling pathways [15–19]. For instance, transient activation of the Src oncoprotein induces a high level of IL-6 production in immortalized breast epithelial cells, which in turn promotes tumorigenesis and generation of CSCs [17]. Moreover, IL-6 can convert differentiated bulk tumor cells into CSC-like cells in multiple molecular subtypes of BC [16], providing support for a causal role of IL-6 in tumorigenesis and cancer progression by inducing and/or maintaining cancer stemness.

The human *X-inactive specific transcript*, or *XIST*, encodes a 17 kb long noncoding RNA (lncRNA), which coats one of the two X chromosomes in female mammals to initiate gene silencing, thereby preventing gene dosage imbalance between females and males [20, 21]. In addition to its well-established role in X chromosome inactivation (XCI) during early embryogenesis, accumulating evidence suggests that aberrant *XIST* expression in post-XCI somatic cells plays a role in tumor development and progression. For example, genetic deletion of *XIST* in mouse hematopoietic and human mammary epithelial cells promotes the formation of highly aggressive myeloproliferative neoplasm and HRAS^G12V^-driven mammary carcinoma respectively [22, 23], suggesting a role of *XIST* expression from the inactive X chromosome (Xi) in protecting somatic cells from oncogenesis. However, in late stage breast tumors or established BC cell lines, the Xi, also called Barr body, is commonly absent, presumably due to the loss of Xi and replication of the active X chromosome (Xa) [24] and/or epigenetic erosion of the Xi [25], leading to the formation of XIST clouds in the nucleus deficient in XCI [26]. Supporting this abnormal function of XIST in post-XCI tumor cells, XIST expression in a wide variety of cancer cells suppresses or promotes tumor growth and/or metastasis [27–29]. Such divergent roles of XIST in cancer development and progression may reflect the fact that XIST functions as a major molecular sponge to repress a plethora of oncogenic or tumor suppressive microRNAs (miRNAs) and lncRNAs, leading to suppression or promotion of tumor growth and metastatic progression in a highly context-dependent manner [27–29].

In parallel with the findings that XIST is aberrantly expressed in BC cells [24, 25], high XIST expression in BC is associated with treatment resistance and poor patient outcomes. For instance, low expression of XIST correlates with cisplatin hypersensitivity and predicts long recurrence-free survival of HER2-negative, stage III BC patients treated with intensive platinum-based chemotherapy [30]. In patients with BRCA1-deficient BC, high XIST expression predicts poor outcomes after high-dose alkylating chemotherapy [31]. This association of high XIST expression and chemoresistance suggests a role of aberrant XIST expression in promoting CSCs, which display intrinsic resistance to a variety of therapeutic agents. Indeed, in a study to evaluate histone deacetylase inhibitors (HDACi) as potential anti-CSC therapy, only the BC cells with low XIST expression exhibit HDACi response in mouse xenograft models, and this response is associated with a significant reduction of CSCs [32]. Despite this evidence, an understanding of how elevated XIST expression promotes CSCs remains elusive.

In this study, we examined *XIST* expression across a spectrum of BC cell lines representing different BC subtypes and investigated the impact of DOX-inducible KD of *XIST* on the maintenance of ALDH^+^ E- and CD24^lo^CD44^hi^ M-like CSCs, as well as tumor growth and tumor-initiating potential in mouse xenograft models of luminal and TN BC. We demonstrate that XIST acts as a master regulator of cytokine-cytokine receptor interactions and drives IL-6 expression from ALDH^-^ bulk tumor cells to regulate ALDH^+^ CSCs in a paracrine fashion. XIST directly binds and suppresses let-7a-2-3p, a member of the let-7 family of miRNAs with tumor suppressor functions [33, 34], leading to markedly elevated IL-6 production in ALDH^-^ BC cells (BCCs) and, to a lesser extent, in ALDH^+^ CSCs. IL-6 derived from the bulk ALDH^-^ BCCs binds to IL6R preferentially expressed on ALDH^+^ CSCs to induce STAT3 activation and expression of key stemness factors c-MYC, KLF4 and SOX9, promoting self-renewal of ALDH^+^ CSCs.

## Results

### Aberrant *XIST* expression promotes ALDH^+^ E-CSCs in luminal and TN BC

**Fig. 1:**
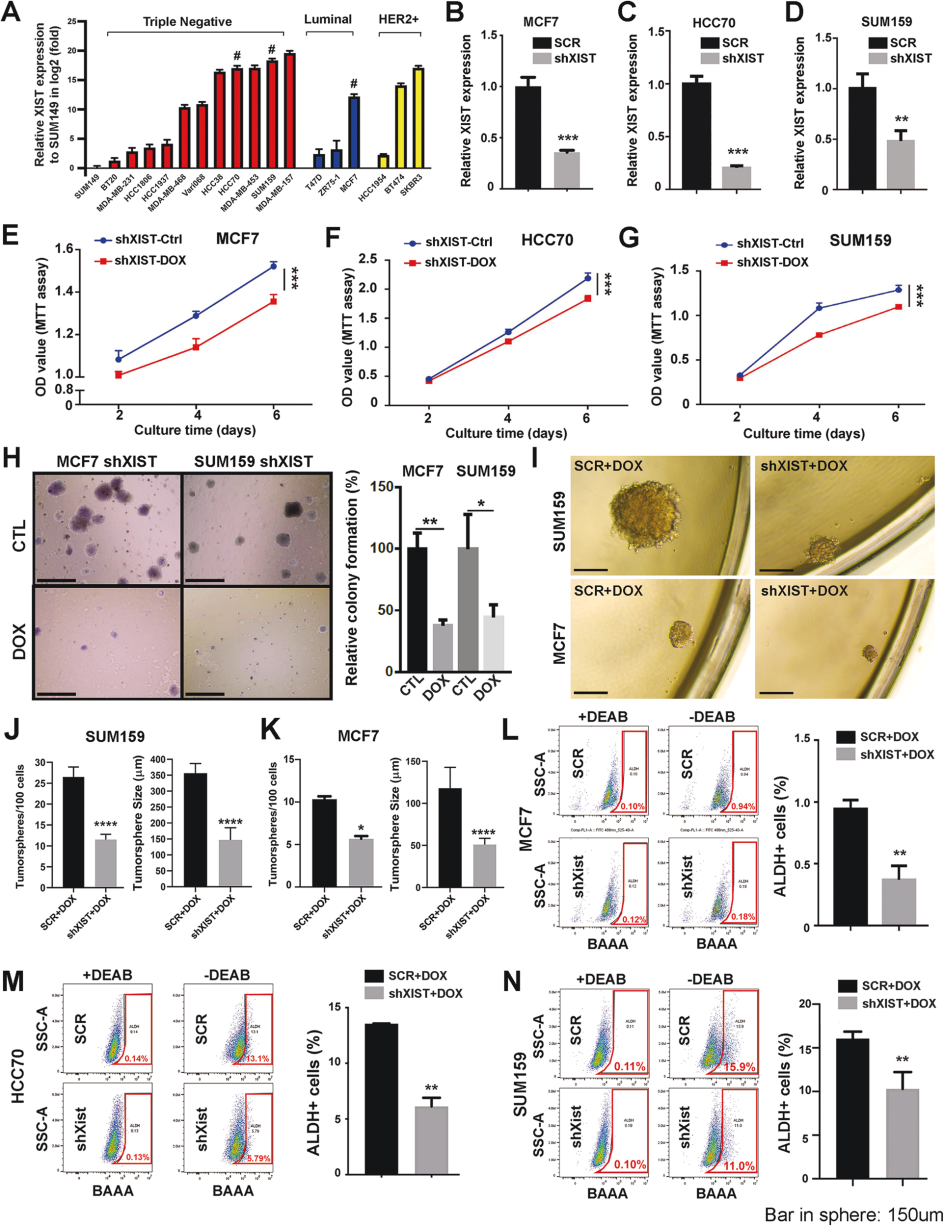
Aberrant *XIST* expression promotes ALDH^+^ E-CSCs in luminal and TN BC cells. **A** Relative expression of *XIST* across different subtypes of BC in triple negative (TN), estrogen receptor positive (ER^+^) luminal, and HER2^+^ BC cell lines. Data are presented as log2 (fold change) over the expression level of XIST in SUM149 (*n* = 3). #: BCCs with relatively high *XIST* expression selected for DOX-inducible *XIST* KD. **B**–**D** qRT-PCR analysis of *XIST* expression in MCF7-shXIST (**B**), HCC70-shXIST (**C**) and SUM159-shXIST (**D**) BCCs vs. the cells expressing a SCR sequence after DOX (1 µg/ml) treatment for 3 days (*n* = 3). (**E**–**G** Cell growth evaluated by MTT assay in MCF7-shXIST (**E**), HCC70-shXIST (**F**) and SUM159-shXIST (**G**) BCCs vs. the corresponding cells expressing a SCR sequence following DOX (1 µg/ml) treatment for 2, 4 and 6 days. **H** Tumor colonies formed in 3D soft agar for SUM159-shXIST and MCF7-shXIST cells in the presence or absence of DOX (1 µg/ml) for 2 weeks were counted in each well of a 6-well plate (*n* = 6), and results are normalized based on the numbers of colonies derived in the absence of DOX. Scale bar: 200 µm. **I**–**K** Tumorsphere formation of SUM159-shXIST (**I**, **J**) and MCF7-shXIST (**I**, **K**) BCCs vs. corresponding SCR cells under ultralow adherent conditions at clonal density in the presence of 1 µg/ml of DOX for 14 days. Tumorspheres with diameter ≥ 40 µm were counted in each plate (*n* = 3) and the sizes of tumorspheres for shXIST vs. SCR cells of SUM159 (**J**) and MCF7 (**K**) were calculated based on 10 randomly selected tumorspheres (*n* = 10). Scale bar: 150 µm. **L**, **N** MCF7-shXIST (**L**), HCC70-shXIST (**M**) and SUM159-shXIST (**N**) BCCs vs. the corresponding cells expressing a SCR sequence were treated with DOX (1 µg/ml) for 3 days and analyzed by ALDEFLOUR assay to determine the proportion of ALDH^+^ CSCs in three biological repeats (*n* = 3). All data are presented as mean ± SD. *P < 0.05, **P < 0.01, ***P < 0.001, ****P < 0.0001.

To explore the roles of aberrant *XIST* expression in BCCs, we examined the relative levels of *XIST* expression in panels of triple negative (TN), estrogen receptor positive (ER^+^) luminal, and HER2^+^ BC cell lines. SUM149, a basal BC cell line derived from an inflammatory BC harboring *BRCA1* mutation (2288delT) [35] expresses minimal level of *XIST* (Fig. 1A). Compared to SUM149, *XIST* is variably expressed in a panel of TNBC cell lines, with high levels of expression found in HCC38, HCC70, MDA-MB-453, SUM159 and MDA-MD-157, while MDA-MB-468 and Vari068 modestly express, and BT20, MDA-MB-231, HCC1806 and HCC1937 BCCs express relatively low levels of *XIST* (Fig. 1A). Such variable *XIST* expression is also observed in luminal (where MCF7 expresses relatively higher level of *XIST* than T47D and ZR75-1) and HER2^+^ (where BT474 and SKBR3 express relatively higher levels of *XIST* than HCC1954) BCCs.

To further examine how *XIST* is expressed in BCCs vs. normal breast epithelial cells, we compared the relative expression of *XIST* in TNBC, luminal, and HER2^+^ BCCs against MCF10A, a non-tumorigenic human breast epithelial cell line. This revealed that the majority of BCCs across different BC subtypes maintain very low levels of *XIST* expression relative to their normal counterpart (Fig. S1A–C). This low *XIST* expression in BCCs reflects the fact that, Xi, where *XIST* is actively expressed/maintained in normal somatic cells, is frequently lost in late-stage BC and BC cell lines [24]. Although the majority of BCCs maintain low *XIST* expression, a panel of TNBC cells including MDA-MD-157, SUM159, MDA-MB-453, HCC70 and HCC38 have abnormal high levels of *XIST* expression (Fig. S1A). Considering the fact that XIST-bound Xi is lost in BCCs [24], aberrant *XIST* expression in a panel of TNBC cells is most likely derived from the active X chromosome or Xa.

To investigate the functional significance of aberrant *XIST* expression in TNBC, which contain higher proportions of E- and M-CSCs relative to luminal BCCs, we established DOX inducible *XIST* KD cell lines in HCC70 and SUM159, two TNBC cell lines representing the basal and mesenchymal subtypes respectively with aberrant *XIST* expression (Fig. 1A). As a subtype control, we use MCF7, which expresses the highest level of *XIST* compared to other luminal BCCs tested, to establish DOX-inducible *XIST* KD luminal BCCs. By quantitative real-time PCR (qRT-PCR) analysis, we confirmed that DOX-induced *XIST* KD in MCF7 (Fig. 1B), HCC70 (Fig. 1C), and SUM159 (Fig. 1D) significantly reduced *XIST* expression compared to the corresponding cells expressing a scrambled sequence (SCR). DOX-induced *XIST* KD modestly inhibited cell growth of MCF7 (Fig. 1E), HCC70 (Fig. 1F), and SUM159 (Fig. 1G) BCCs grown under 2D adherent conditions as evaluated by MTT assays. However, under 3D soft-agar culturing conditions, DOX-induced *XIST* KD markedly impaired colony-forming capacity of MCF7 and SUM159 BCCs (Fig. 1H).

We next measured tumorsphere formation at clonal density, a property of CSCs, in MCF7 and SUM159 BCCs with or without *XIST* KD. To ensure tumorsphere formation at clonal density, live (DAPI^-^) SUM159 and MCF7 BCCs expressing shXIST hairpin vs. a SCR sequence were FACS sorted at 10 (for SUM159) or 20 (for MCF7) cells/well into ultralow-attachment 96-well plates preloaded with serum-free mammosphere medium containing DOX (1 µg/ml). Following DOX-induced *XIST* KD, both SUM159 (Fig. 1I, J) and MCF7 (Fig. 1I, K) BCCs exhibited significantly reduced tumorsphere-forming capacity, characterized by significantly reduced numbers and size of tumorspheres formed. This suggests that *XIST* expression is required for maintenance of self-renewal and/or proliferative capacity of CSCs in serum-free, anchorage-independent conditions. Enumeration of ALDH^+^ BCSCs by ALDEFLOUR assay in MCF7 (Fig. 1L), HCC70 (Fig. 1M) and SUM159 (Fig. 1N) BCCs revealed that DOX-induced *XIST* KD significantly decreases the proportion of ALDH^+^ E-CSCs in each cell line, suggesting that lncRNA XIST is required to maintain proliferative ALDH^+^ E-CSCs in luminal and TN BC.

To rule out potential off-target effects associated with a single shXIST hairpin sequence, we employed additional lentiviral vectors expressing three different DOX-inducible shXIST hairpins (shXIST-7769, shXIST-1017, shXIST-1352). DOX-induced KD of *XIST* in SUM159 BCCs with three distinct shXIST hairpins significantly decreasing *XIST* expression compared to the cells expressing a SCR sequence (Fig. S1D). Further analysis of SUM159 cells expressing shXIST-7769 (Fig. S1E), shXIST-1017 (Fig. S1F) and shXIST-1352 (Fig. S1G) vs. SCR confirmed that DOX-induced *XIST* KD with three distinct hairpins all significantly reduced the percentage of ALDH^+^ CSCs and tumorsphere-forming capacity in shXIST-7769 vs. SCR (Fig. S1H-J**)**. Together, these studies indicate that XIST expression promotes CSC activity and DOX-induced *XIST* KD significantly reduces ALDH^+^ E-CSCs in BCCs derived from luminal and basal/mesenchymal BC.

### XIST is required to maintain CD24^-/lo^CD44^+/hi^ M-CSCs in TNBC by suppressing luminal differentiation

Given a role of XIST in promoting proliferative ALDH^+^ E-CSCs in luminal and TN BCCs, we next examined whether aberrant *XIST* expression is required to maintain the more quiescent M-like CSCs characterized by CD24^-/lo^CD44^+/hi^ expression [9]. DOX-induced *XIST* KD in MCF7 luminal BCCs did not significantly affect the percentage of CD24^-/lo^CD44^+/hi^ M-like CSCs (Fig. S2A–C). However, in HCC70 (Fig. S2D–G) and SUM159 (Fig. S2H–K) BCCs, DOX-induced *XIST* KD significantly decreased the percentage of CD24^-/lo^CD44^+/hi^ M-CSC-like population compared to the cells expressing a SCR sequence. This reduction of M-CSC-like cells is mainly attributed to the significantly increased population of cells expressing epithelial marker CD24 (CD24^+^CD44^+^) in HCC70 (Fig. S2G) and SUM159 (Fig. S2K) upon DOX-induced *XIST* KD. This suggests that XIST inhibits luminal differentiation in TNBC cells. Together, our studies demonstrate that high *XIST* expression plays a role in promoting proliferative, ALDH^+^ E-CSCs in both luminal and TN BC. High *XIST* expression is also required to maintain CD24^-/lo^CD44^+/hi^ M-CSCs in TNBC by inhibiting luminal differentiation.

### DOX-induced *XIST* KD significantly abrogates tumor growth and tumor-initiating potential in xenograft models of luminal and TN BC

**Fig. 2:**
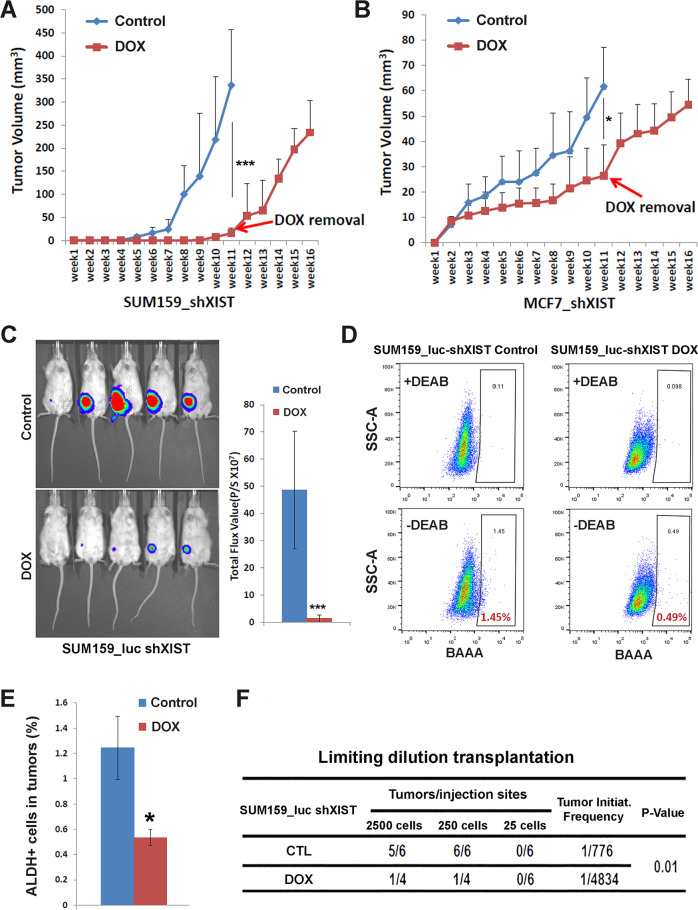
DOX-inducible KD of XIST significantly suppresses tumor growth and tumor-initiating potential in NOD/SCID mice. **A**, **B** SUM159-shXIST (**A**) and MCF7-shXIST (**B**) BCCs engrafted in NOD/SCID mice were each randomized in two groups containing 6 mice (*n* = 6), which were treated with or without DOX-containing water and monitored for tumor growth for 11 weeks. After 11 weeks, DOX water was removed, and tumor growth of SUM159-shXIST and MCF7-shXIST BCCs was continuously monitored for 5 weeks. **C** Mice implanted with SUM159_Luc-shXIST cells were randomized in two groups (*n* = 5) and fed with or without DOX containing water for 13 weeks. Tumor growth was monitored by bioluminescence imaging of luciferase activity. **D**, **E** Tumor cells dissociated from tumors of Control and DOX treated mice as shown in Figure C were subjected to ALDEFLOUR assay (**D**) to determine the percentage of ALDH^+^ CSCs in the tumor (**E**). **F** H2Kd^+^ mouse stromal cells from pooled tumors of Control or DOX treated mice were gated out by FACS, and live (DAPI^-^) H2Kd^-^ SUM159 tumor cells were sorted and transplanted into the #4 mammary fat pad of secondary NOD/SCID mice bilaterally (*n* = 6) with three different dilutions (2500, 250 and 25 cells/injection). Tumor appearance in each group of mice was monitored for 3 months to calculate tumorigenicity of Control or DOX treated tumor cells. Data are presented as mean ± SD. *, ***P < 0.05 and 0.001 respectively vs. Control.

To determine if DOX-induced KD of *XIST* affects tumor growth in vivo, we injected SUM159 and MCF7 BCCs harboring the DOX-inducible shXIST hairpin sequence (V2THS_92229) into the #4 mammary fat pad (MFP) of 6-8-week-old female NOD/SCID mice, which were randomized in two cohorts (*n* = 5 per cohort) and fed with or without DOX-containing water for 11 weeks, starting one day after tumor cell injection. As shown in Fig. 2A, mice implanted with SUM159_shXIST cells without DOX treatment (Control) generated palpable mammary tumors at week 4 post injection, which grew rapidly to reach a mean tumor volume of 336.45 ± 120.27 mm^3^ (Mean ± SD) at week 11. In contrast, mice fed with DOX-containing water for 11 weeks exhibited markedly reduced tumor growth, with a mean tumor volume of 15.52 ± 11.46 mm^3^ (Mean ± SD) at week 11. Notably, following DOX withdrawal after week 11, SUM159_shXIST tumor cells resumed rapid tumor growth, suggesting that DOX-induced *XIST* KD did not kill SUM59 tumor cells, but rather impaired their growth. Similar results were observed in NOD/SCID mice implanted with MCF7_shXIST BCCs (Fig. 2B), where mice subjected to 11-week DOX treatment displayed significantly inhibited tumor growth vs. controls, while DOX withdrawal after week 11 resulted in resumption of rapid tumor growth. This DOX-induced tumor growth retardation is not due to the direct effect of DOX, as this drug had no effect on tumor growth of parental SUM159 (Fig. S3A) or MCF7 (Fig. S3B) xenografts.

To further substantiate the role of XIST in regulating tumor growth and CSC activity, we implanted SUM159-shXIST cells with stable expression of firefly luciferase into NOD/SCID mice and monitored mammary tumor growth by bioluminescence imaging in mice fed with or without DOX-containing water. We observed a similar inhibitory effect on tumor growth upon DOX-induced *XIST* KD, assessed by luciferase-elicited bioluminescence imaging (Fig. 2C) and measurement of tumor volume (Fig. S3C) following DOX vs. control water treatment. Furthermore, tumors isolated from DOX-treated XIST KD mice contained a significantly reduced percentage of ALDH^+^ cells compared to the tumors of control mice (Fig. 2D, E), suggesting a role of XIST in maintaining ALDH^+^ CSCs in vivo.

To directly assess the impact of *XIST* KD on tumor-initiating potential, we performed serial dilution transplantation using H2Kd^-^ tumor cells dissociated from SUM159 *XIST* KD or control tumors into secondary NOD/SCID mice and calculated tumor initiating frequency based on subsequent tumor development.This assay revealed that DOX-induced *XIST* KD in primary tumor cells resulted in a 6-fold decrease in tumor initiation frequency (Fig. 2F) as well as reduced tumor growth upon implantation of 2500 (Fig. S3D) or 250 (Fig. S3E) tumor cells.These in vivo studies confirmed that loss of XIST in MCF7 and SUM159 BCCs suppresses tumor growth and tumorigenic potential, presumably due to the depletion of proliferative ALDH^+^ E-CSCs in MCF7 (Fig. 1L) and E- (Fig. 2D, E) and M-CSCs (Fig. S2H–K) in SUM159.

### XIST is a master regulator of cytokine-cytokine receptor interactions

**Fig. 3:**
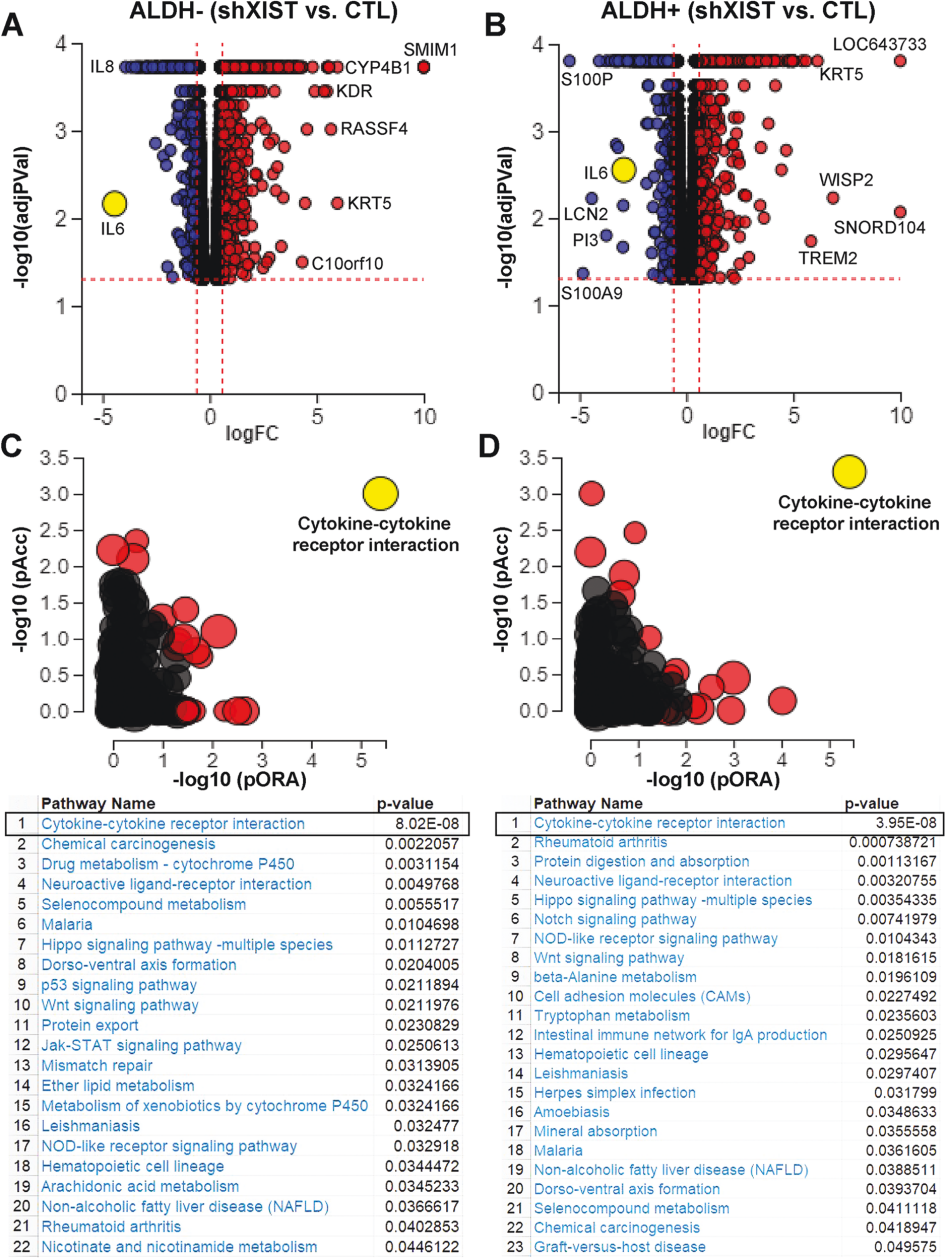
XIST acts as a master regulator of cytokine-cytokine receptor interaction in ALDH^-^ and ALDH^+^ BC cells. **A**, **B** Significantly downregulated (log_2_FC ≤ 0.6, blue dots) and upregulated (log_2_FC ≥ 0.6, red dots) genes in ALDH^-^ (**A**) and ALDH^+^ (**B**) cell populations of SUM159 shXIST BCCs upon 3-day treatment with DOX (1 µg/ml) vs. CTL (no DOX treatment), with data presented as volcano plots. **C**, **D** Impact analyses based on the over-representation of differentially expressed genes in a given pathway (pORA) and the perturbation of that pathway computed by propagating the measured expression changes across the pathway topology (pAcc) were conducted to determine the significantly changed pathways in ALDH^-^ and ALDH^+^ BCCs following treatment with DOX vs. CTL, which identify 22 and 23 significantly changed pathways in ALDH^-^ (**C**) and ALDH^+^ (**D**) BCCs respectively.

To explore the potential mechanisms by which XIST regulates tumor growth and CSC activity, we FACS sorted ALDH^-^ and ALDH^+^ cells from DOX-untreated SUM159-shXIST cells, which were replated and treated with or without DOX for 3 days and subjected to next-generation RNA sequencing (RNAseq). Using DOX-untreated samples as controls, we characterized the significantly downregulated (log_2_FC ≤ 0.6, blue dots) and upregulated (log_2_FC ≥ 0.6, red dots) genes in ALDH^-^ (Fig. 3A) and ALDH^+^ (Fig. 3B) cells upon DOX-induced *XIST* KD, with data presented as volcano plots. These genes represent 22 and 23 signaling pathways significantly changed in ALDH^-^ (Fig. 3C) and ALDH^+^ (Fig. 3D) cells, respectively. Interestingly, in both ALDH^-^ and ALDH^+^ BCCs, cytokine-cytokine receptor interaction emerged as the most significantly affected pathway upon DOX-induced *XIST* KD.

Further heatmap analysis and mapping of the significantly changed genes involved in cytokine-cytokine receptor interaction mediated by the CXC or CC chemokine subfamilies, gp130 (IL6ST) or IL-3RB (CSFRB) shared hematopoietins, and PDGF, TNF, and TGFβ families in ALDH^-^ (Fig. S4A) and ALDH^+^ (Fig. S4B) BCCs revealed that a variety of proinflammatory cytokine/chemokine genes with tumor supportive functions including *IL-6* [16, 17], *IL-8* [19], *IL-1A/B* [36], *LIF* (leukemia inhibitory factor) [37], *CSF3* [38], *CXCL2* [39], *CXCL3* [40], etc. are significantly downregulated, whereas the cytokine or chemokine genes with tumor suppressive properties including *CCL5* [41], *IL-7* [42], *IL-15* [42, 43], *IL-18* [43, 44], etc. are significantly upregulated upon *XIST* knockdown. This unbiased RNAseq analyses suggests that aberrant *XIST* expression may function as a master regulator augmenting pro-inflammatory and suppressing anti-inflammatory cytokine signaling suggesting a possible causative role of these pathways in mediating XIST’s effects on CSCs, tumor initiation and tumor growth.

### IL-6, but not IL-8, plays a prominent role mediating XIST regulation of ALDH^+^ E-CSCs

**Fig. 4:**
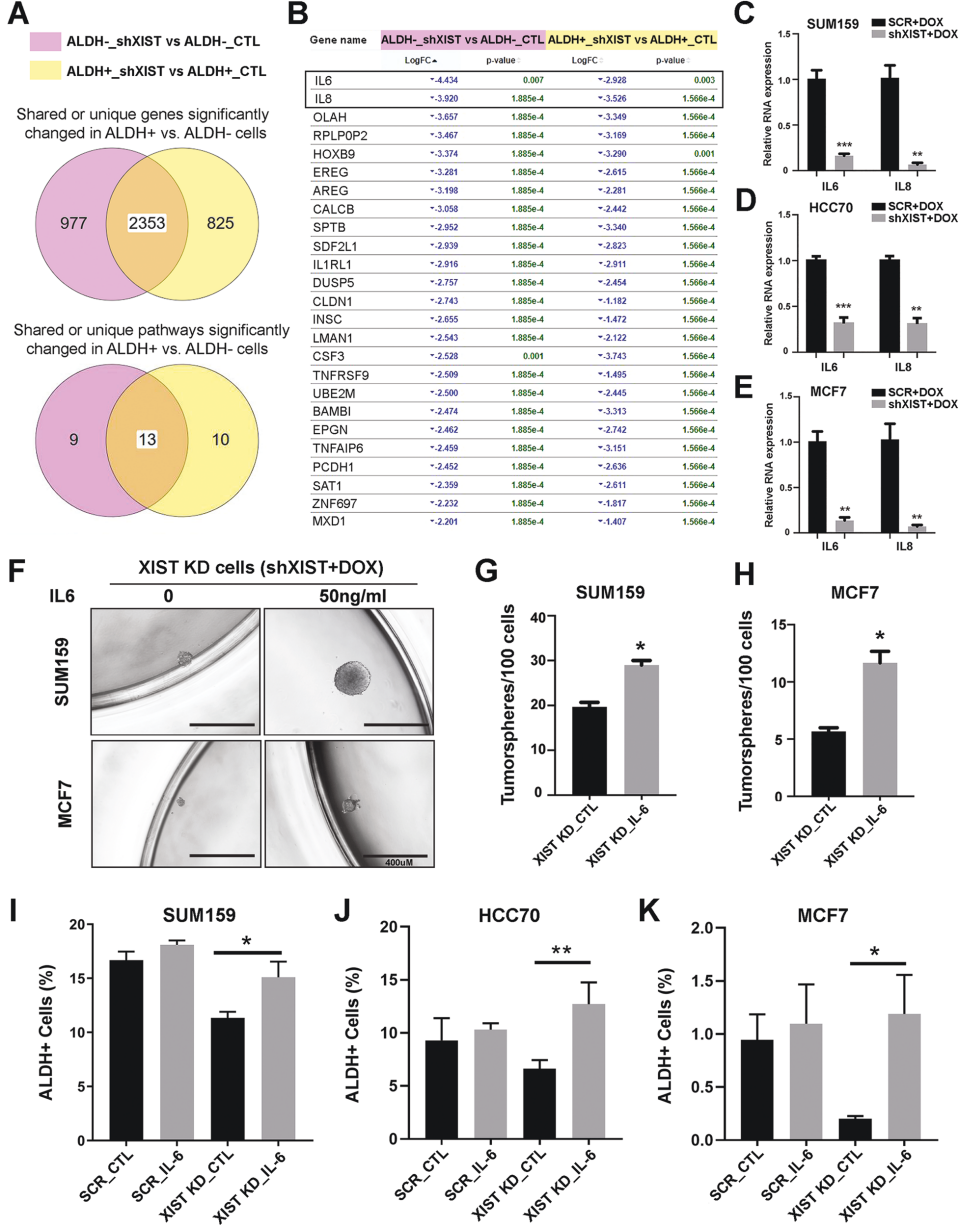
IL-6, but not IL-8, plays a prominent role mediating XIST regulation of ALDH^+^ E-CSCs. **A** Venn Diagram analysis of the significantly changed genes and pathways in SUM159 ALDH^-^ and ALDH^+^ cells upon DOX-induced XIST KD. **B** Meta-analysis of downregulated genes shared in ALDH^-^ vs. ALDH^+^ cells after DOX-induced *XIST* KD identified *IL6* and *IL8* as the top 2 genes most significantly downregulated in ALDH^-^ BCCs and, to a less extent, in ALDH^+^ CSCs. **C**–**E** Validation of *IL6* and *IL8* expression in SUM159 (**C**), HCC70 (**D**), and MCF7 (**E**) BCCs following DOX-induced *XIST* KD by qRT-PCR (*n* = 3). **F**–**H** Supplementation of human IL-6 at 50 ng/mL rescues the impaired tumorsphere formation of SUM159-shXIST (**F**, **G**) and MCF7-shXIST BCCs (**F**, **H**) treated with DOX (1 µg/ml). **I**–**K** Addition of exogenous IL-6 vs. CTL (water) significantly rescues the decreased percentage of ALDH^+^ CSCs in DOX-treated SUM159 (**I**), HCC70 (**J**), and MCF7 (**K**) BCCs expressing shXIST but not SCR sequence. Experiments were repeated three times with similar results and data from a representative experiment are shown. All data are presented as mean ± SD. *p < 0.05, **p < 0.01, ***p < 0.001, respectively.

To elucidate the significantly changed genes and pathways shared by ALDH^-^ and ALDH^+^ BCCs or differentially expressed in ALDH^+^ CSCs upon DOX-induced *XIST* KD, we performed Venn Diagram meta-analysis, which identified 2353 genes shared in ALDH^-^ and ALDH^+^ BCCs and 825 genes in ALDH^+^ CSCs (Fig. 4A, upper panel). These genes represent 13 and 10 signaling pathways respectively (Fig. 4A, lower panel), and cytokine-cytokine receptor interaction remained as the most significantly changed pathway in ALDH^-^ and ALDH^+^ cells upon *XIST* KD (Fig. S5A). Further examination of top 25 downregulated genes in ALDH^-^ vs. ALDH^+^ cells upon *XIST* KD identified *IL-6* and *IL-8* as the top two genes most significantly inhibited in ALDH^-^ bulk tumor cells, and these two cytokine genes are also downregulated, to a lesser extent, in ALDH^+^ CSCs (Fig. 4B). As ALDH^-^ BCCs constitute the majority of tumor cells in the tumor mass, these data suggest that loss of XIST in BCCs significantly reduced the production of proinflammatory cytokine IL-6 and IL-8 in the tumor milieu, which may be responsible for the impaired CSC activities. We also examined the 825 significantly changed genes differentially expressed in ALDH^+^ CSCs (Fig. 4A, upper panel), which identified *S100P* and *S100A9* as the top two genes most significantly inhibited in ALDH^+^ CSCs but not ALDH^-^ bulk tumor cells upon *XIST* KD (Fig. S5B). This suggests that S100P/A9 inflammatory proteins may have cell-autonomous roles mediating XIST regulation of ALDH^+^ CSCs.

To validate if DOX-induced *XIST* KD indeed affect gene expression of *IL-6*, *IL-8*, *S100P* and *S100A9* in luminal and TN BCCs, we next performed qRT-PCR analysis of these genes in DOX-treated SUM159, HCC70 and MCF7 BCCs expressing shXIST vs. a SCR sequence. These studies confirmed the RNAseq data indicating that *IL-6* and *IL-8* gene expression are significantly reduced upon DOX-induced *XIST* KD (Fig. 4C–E) across multiple BCC subtypes. Therefore, aberrant *XIST* expression in BCCs may promote ALDH^+^ CSCs through IL-6 or IL-8 mediated proinflammatory signaling, which have previously been implicated in the regulation of CSCs, metastasis, and therapeutic resistance[17–19].

Further validation of *S100P* and *S100A9* gene expression revealed that, upon DOX-induced *XIST* KD, *S100P* and *S100A9* are both significantly downregulated in SUM159 (Fig. S5C), but not MCF7 (Fig. S5D) BCCs. In HCC70, DOX-induced *XIST* KD significantly reduces *S100P* but not *S100A9* expression (Fig. S5E). This cell-specific inhibition of *S100P* expression upon DOX-induced *XIST* KD suggests that S100P inflammatory protein may play more significant role in TN than in luminal BCCs.

The findings that *IL-6* and *IL-8* gene expression are consistently downregulated in MCF7, HCC70 and SUM159 BCCs following *XIST* KD prompted us to explore the functional significance of IL-6 and IL-8 cytokines in mediating XIST regulation of CSCs. We determined whether exogenous IL-6 or IL-8 rescues the impaired sphere-forming capacity of SUM159 and MCF7 BCCs upon *XIST* KD. While 50 ng/ml exogenous IL-6 had no significant impact on sphere-forming capacity of DOX-treated SUM159 (Fig. S6A) and MCF7 (Fig. S6B) cells expressing a SCR sequence, addition of IL-6 significantly rescued spheroid-forming capacity of DOX-treated SUM159-shXIST and MCF7-shXIST BCCs (Fig. 4F–H). In contrast, addition of IL-8 at 50 ng/ml failed to significantly rescue spheroid-forming capacity of SUM159 BCCs with *XIST* KD (Fig. S6C). This suggests that IL-6, but not IL-8, plays a prominent role in mediating XIST CSC regulation. Indeed, addition of IL-6 at 50 ng/ml to DOX-treated SUM159-shXIST BCCs grown in 2D adherent culturing conditions for 3 days significantly rescued the decreased proportion of ALDH^+^ CSCs, while ALDH^+^ CSCs in SUM159 BCCs expressing a SCR sequence only had a small but not significant increase following IL-6 treatment (Fig. 4I). Similar results were obtained in HCC70 (Fig. 4J) and MCF7 (Fig. 4K), where addition of IL-6 to DOX-induced *XIST* KD cells significantly rescued the decreased proportion of ALDH^+^ CSCs, while addition of IL-6 to DOX-treated cells expressing a SCR sequence had no significant effect. These data demonstrate a functional role of IL-6 in mediating XIST regulation of ALDH^+^ CSCs in luminal and TNBC. Moreover, XIST-driven IL-6 cytokine production appears to be sufficient for maintaining ALDH^+^ CSCs, as exogenous IL-6 added to MCF7, HCC70 and SUM159 BCCs without *XIST* KD failed to significantly increase the proportion of ALDH^+^ CSCs in each cell line.

We next examined whether exogenous IL-6 rescues the decreased proportion of CD24^lo^CD44^hi^ M-like CSCs in SUM159 BCCs with *XIST* KD. DOX-treated SUM159-shXIST cells incubated with IL-6 (50 ng/ml) for 3 days did not exhibit significantly increased CD24^lo^CD44^hi^ M-like CSCs, although IL-6 treatment modestly but significantly increased CD24^lo^CD44^hi^ M-like CSCs in DOX-treated SUM159 BCCs expressing a SCR sequence (Fig. S6D). This suggests that loss of CD24^lo^CD44^hi^ M-like CSCs (due to increased CD24 expression and luminal differentiation) following DOX-induced *XIST* KD in SUM159 is not attributed to impaired IL-6 cytokine expression. Together, our studies support a specific role of XIST-driven IL-6 expression in maintaining ALDH^+^ E- but not CD24^lo^CD44^hi^ M-like CSCs.

### XIST activates *IL-6* expression by suppressing let-7a-2-3p

Given our findings that DOX-induced *XIST* KD in ALDH^-^ and ALDH^+^ cells most significantly affected the genes involved in cytokine-cytokine receptor signaling (Fig. 3A–D), we next ask if lncRNA XIST indirectly regulates the expression of these genes (i.e., *IL-6* and *IL-8*) through its traditional XCI function. As the majority of genes that were significantly changed in ALDH^-^ and ALDH^+^ cells upon DOX-induced *XIST* KD are protein coding genes, we examined the expression of 816 X-linked protein coding genes (Table S3) in ALDH^-^ and ALDH^+^ cells following DOX-induced *XIST* KD. This revealed that 696 out of 816 X-linked protein coding genes were not significantly changed upon DOX-induced *XIST* KD in ALDH^-^ and ALDH^+^ cells (Fig.S6E). Further analysis of significantly changed X-linked protein coding genes in ALDH^-^ or ALDH^+^ cells revealed that 56 genes were upregulated, and 45 genes downregulated in ALDH^-^ cells (Table S4), and 40 genes were upregulated, and 45 genes downregulated in ALDH^+^ cells (Table S5) following DOX-induced *XIST* KD. Similarly, in 66 X-linked protein coding genes that were significantly changed in both ALDH^-^ and ALDH^+^ cells, 34 genes were upregulated, and 32 genes downregulated (Table S6). This meta-analysis strongly argues against an XCI function of *XIST* accounting for its effects on CSCs since *XIST* knockdown had no preferential effect on X linked genes.

**Fig. 5:**
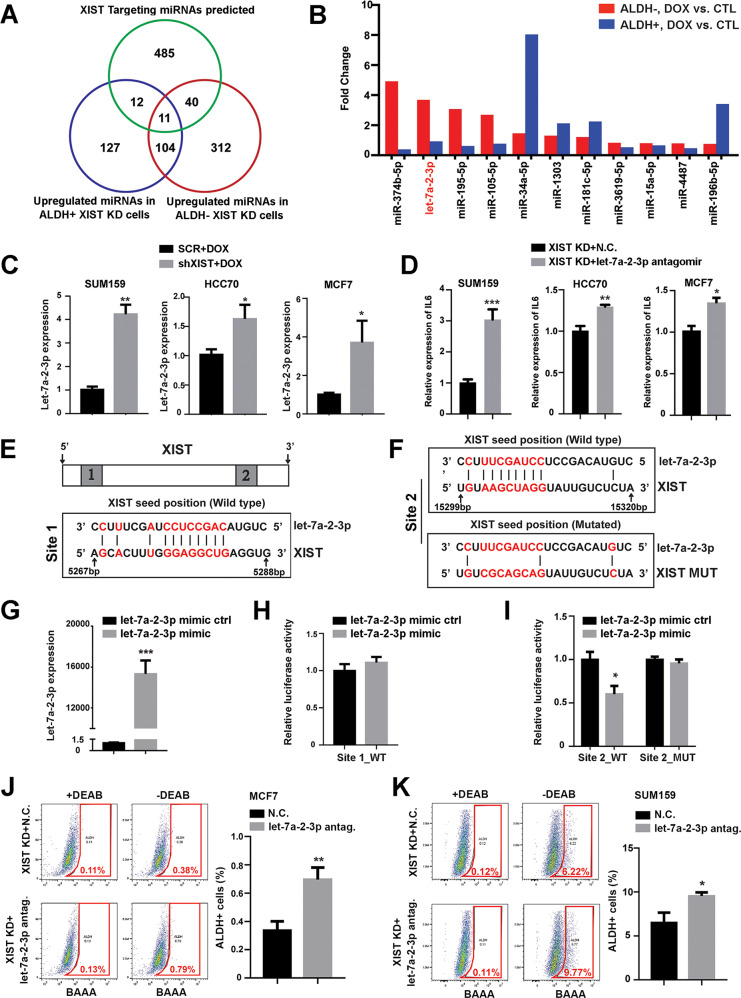
Aberrant XIST expression promotes *IL-6* expression and ALDH^+^ CSCs through its repression of let-7a-2-3p. **A**, **B** Identification of candidate miRNAs targeted by XIST via Venn Diagram analysis of the significantly upregulated miRNAs in ALDH^-^ and ALDH^+^ cells of SUM159-shXIST BCCs upon DOX vs. CTL treatment and XIST target miRNAs predicted by the LNCipedia database (**A**) and 11 potential XIST target miRNAs shared in ALDH^-^ vs. ALDH^+^ BCCs are plotted (**B**). **C** qRT-PCR validation of let-7a-2-3p expression in DOX-treated SUM159, HCC70, and MCF70 BCCs expressing shXIST vs. a SCR sequence (*n* = 3). **D** Relative *IL-6* expression in DOX-treated SUM159-shXIST, HCC70-shXIST and MCF7-shXIST BCCs after transfected with a let-7a-2-3p antagomir vs. negative control RNA (*n* = 3). **E**, **F** Sequence alignments of XIST Site 1 (**E**) and Site 2 (**F**) with highest probability of binding let-7a-2-3p. **G**, **H** let-7a-2-3p expression in SUM159 BCCs after transfected with a let-7a-2-3p mimic vs. control sequence (**G**) and luciferase reporter activity of SUM159 BCCs transfected with the luciferase reporter plasmid harboring XIST Site 1 (WT) at the presence of a let-7a-2-3p mimic vs. control sequence (**H**). **I** Luciferase reporter activity of SUM159 BCCs transfected with the luciferase reporter plasmid harboring XIST WT or mutant Site 2 at the presence of a let-7a-2-3p mimic vs. control sequence. **J**, **K** DOX-treated MCF7-shXIST (**J**) and SUM159-shXIST (**K**) BCCs were transfected with a let-7a-2-3p antagomir vs. N.C. and the percentage of ALDH^+^ CSCs was examined by ALDEFLOUR assay in three independent experiments. Experiments were repeated three times with similar results and data from a representative experiment are shown and presented as mean ± SD. *p < 0.05, **p < 0.01, ***p < 0.001, respectively.

Recent studies have indicated that XIST functions as a competing endogenous RNA (ceRNA) or molecular sponge for many miRNAs [27–29]. To identify potential miRNAs directly targeted by XIST, ALDH^-^ and ALDH^+^ BCCs from DOX-untreated SUM159-shXIST cells were sorted, replated, and treated with or without DOX for 3 days, and subjected to GeneChip™ miRNA Array analysis. As loss of XIST de-represses its target miRNAs, we extracted the significantly upregulated miRNAs in ALDH^-^ and ALDH^+^ BCCs treated with DOX vs. CTL (log2 FC ≥ 0.6). This identified 467 and 254 significantly upregulated miRNAs in ALDH^-^ (Table S7) and ALDH^+^ (Table S8) BCCs upon XIST KD. Meta-analysis of these two sets of miRNAs with XIST target miRNAs (Table S9) predicted by the LNCipedia database (https://lncipedia.org/) revealed 11 potential XIST targeted miRNAs that were significantly upregulated in both ALDH^-^ bulk tumor cells and ALDH^+^ CSCs upon *XIST* KD (Fig. 5A). Interestingly, let-7a-2-3p, a member of let-7 miRNAs, is markedly upregulated in ALDH^-^ and, to a lesser extent, in ALDH^+^ BCCs upon *XIST* KD (Fig. 5B). As let-7 miRNAs including let-7a directly repress IL-6 cytokine production in breast epithelial cells [17], our data suggest a potential activity of XIST by repressing let-7a-2-3p in ALDH^-^ bulk tumor cells to increase IL-6 cytokine production, which in turn promotes ALDH^+^ CSCs. qRT-PCR validation of let-7a-2-3p expression in SUM159, HCC70 and MCF7 BCCs confirmed that let-7a-2-3p is consistently upregulated following DOX-induced *XIST* KD (Fig. 5C), suggesting a role of lncRNA XIST in repressing let-7a-2-3p across different subtypes of BCCs.

In addition to let-7a-2-3p, we also validated 10 other potential miRNA targets of XIST (Fig. 5B). qRT-PCR analyses of DOX-treated SUM159-shXIST vs. SCR cells confirmed that miR-374b-5p, miR-181c-5p and miR-1303 are significantly upregulated, while the rest of miRNAs were not significantly changed upon *XIST* KD (Fig. S7). This suggests that miR-374b-5p, miR-181c-5p and miR-1303 may serve as additional miRNA targets mediating XIST regulation of *IL-6* gene expression and ALDH^+^ CSCs.

We next investigated whether introduction of miRNA antagomirs against let-7a-2-3p, miR-374b-5p, miR-181c-5p and miR-1303 rescues the reduced *IL-6* gene expression in BCCs subjected to DOX-induced *XIST* KD. Transfection of a let-7a-2-3p antagomir vs. a negative control sequence (N.C.) into DOX-treated SUM159-shXIST (Fig. S8A), HCC70-shXIST (Fig. S8B) and MCF7-shXIST (Fig. S8C) BCCs all significantly inhibited let-7a-2-3p expression, leading to significantly increased *IL-6* gene expression in each XIST KD cell line (Fig. 5D). This confirms that let-7a-2-3p serves as a specific miRNA targeted by XIST to promote IL-6 cytokine production in different subtypes of BCCs. Introduction of miRNA antagomirs against miR-374b-5p, miR-181c-5p or miR-1303 vs. N.C. into DOX-treated SUM159-shXIST BCCs significantly inhibited the expression of each corresponding miRNA (Fig. S8D–F). However, these specific miRNA antagomirs failed to rescue the impaired *IL-6* mRNA expression (Fig. S8G, H) and proportion of ALDH^+^ CSCs (Fig. S8I, J). Thus, let-7a-2-3p, but not miR-374b-5p, miR-181c-5p or miR-1303, is the specific miRNA targeted by XIST that drives IL-6 cytokine production.

### Molecular mapping of functional let-7a-2-3p binding sites in XIST

To characterize the specific XIST sequences that interact with let-7a-2-3p, we employed the TargetScan database to search for potential let-7a-2-3p binding sites in XIST, which identified two predicted sites (Site 1 and Site 2) with highest probability of binding let-7a-2-3p (Fig. 5E, F). To determine the functional significance of these two XIST sites for interaction with let-7a-2-3p, we cloned the corresponding *XIST* cDNA fragment containing Site 1 or Site 2 (Table S2) into the PmirGLO dual-luciferase miRNA target expression vector, and subsequently transfected these constructs into SUM159 BCCs to evaluate the capacity of these *XIST* cDNA fragments to suppress luciferase reporter activity when co-transfected with a let-7a-2-3p mimic vs. control RNA (ctrl). Although introduction of let-7a-2-3p mimic vs. ctrl into SUM159 BCCs markedly boosted let-7a-2-3p expression (Fig. 5G), let-7a-2-3p mimic selectively inhibited luciferase reporter activity of SUM159 BCCs expressing the luciferase reporter plasmid containing Site 2 (Fig. 5I), but not Site 1 (Fig. 5H). This suggests that Site 2 serves as a functional region of XIST that interacts with let-7a-2-3p, leading to suppressed luciferase reporter activity. Further site-directed mutagenesis of Site 2 (Fig. 5F, lower panel) abolished let-7a-2-3p mimic induced suppression of luciferase reporter activity (Fig. 5I), confirming that Site 2 of XIST acts as a functional region mediating its repression of let-7a-2-3p, leading to increased IL-6 production in BCCs.

To further demonstrate that increased let-7a-2-3p expression in BCCs with *XIST* KD is responsible for the inhibition of ALDH^+^ CSCs, we transfected DOX-treated SUM159-shXIST, HCC70-shXIST and MCF7-shXIST BCCs with a let-7a-2-3p antagomir vs. N.C. and performed ALDEFLOUR assays three days after let-7a-2-3p antagomir transfection. Introduction of let-7a-2-3p antagomir vs. N.C. significantly increased ALDH^+^ CSCs in MCF7 (Fig. 5J), SUM159 (Fig. 5K) and HCC70 (Fig. S9A) BCCs with *XIST* KD. In contrast to BCCs harboring *XIST* KD, introduction of let-7a-2-3p antagomir vs. N.C. in DOX-treated MCF7 (Fig. S9B) or SUM159 (Fig. S9C) BCCs expressing a SCR sequence failed to significantly increase ALDH^+^ CSCs. This suggests that let-7a-2-3p is repressed by aberrant XIST expression in MCF7 and SUM159 BCCs, rendering them refractory to let-7a-2-3p inhibitor treatment.

As introduction of let-7a-2-3p antagomir into different BCCs harboring *XIST* KD significantly rescues the reduced proportion of ALDH^+^ CSCs (Fig. 5J, K, S9A), we next ask whether let-7a-2-3p antagomir could rescue the reduced CD24^lo^CD44^hi^ M-CSCs in HCC70 basal BCCs subjected to DOX-induced *XIST* KD (Fig. S2D-G). Contrary to significantly increased ALDH^+^ E-CSCs induced by let-7a-2-3p antagomir treatment (Fig. S9A), introduction of let-7a-2-3p antagomir vs. N.C. in DOX-treated HCC70-shXIST cells failed to increase CD24^lo^CD44^hi^ M-CSCs (Fig. S9D). This suggests that XIST-mediated repression of let-7a-2-3p specifically regulates ALDH^+^ E- but not CD24^lo^CD44^hi^ M-CSCs in TNBC. Together, our studies demonstrate a specific role of aberrant XIST expression in luminal and TN BCCs to repress let-7a-2-3p, leading to increased IL-6 cytokine production to promote ALDH^+^ E- but not CD24^lo^CD44^hi^ M-CSCs.

### XIST promotes IL-6 driven STAT3 activation and expression of c-MYC, KLF4 and SOX9

**Fig. 6:**
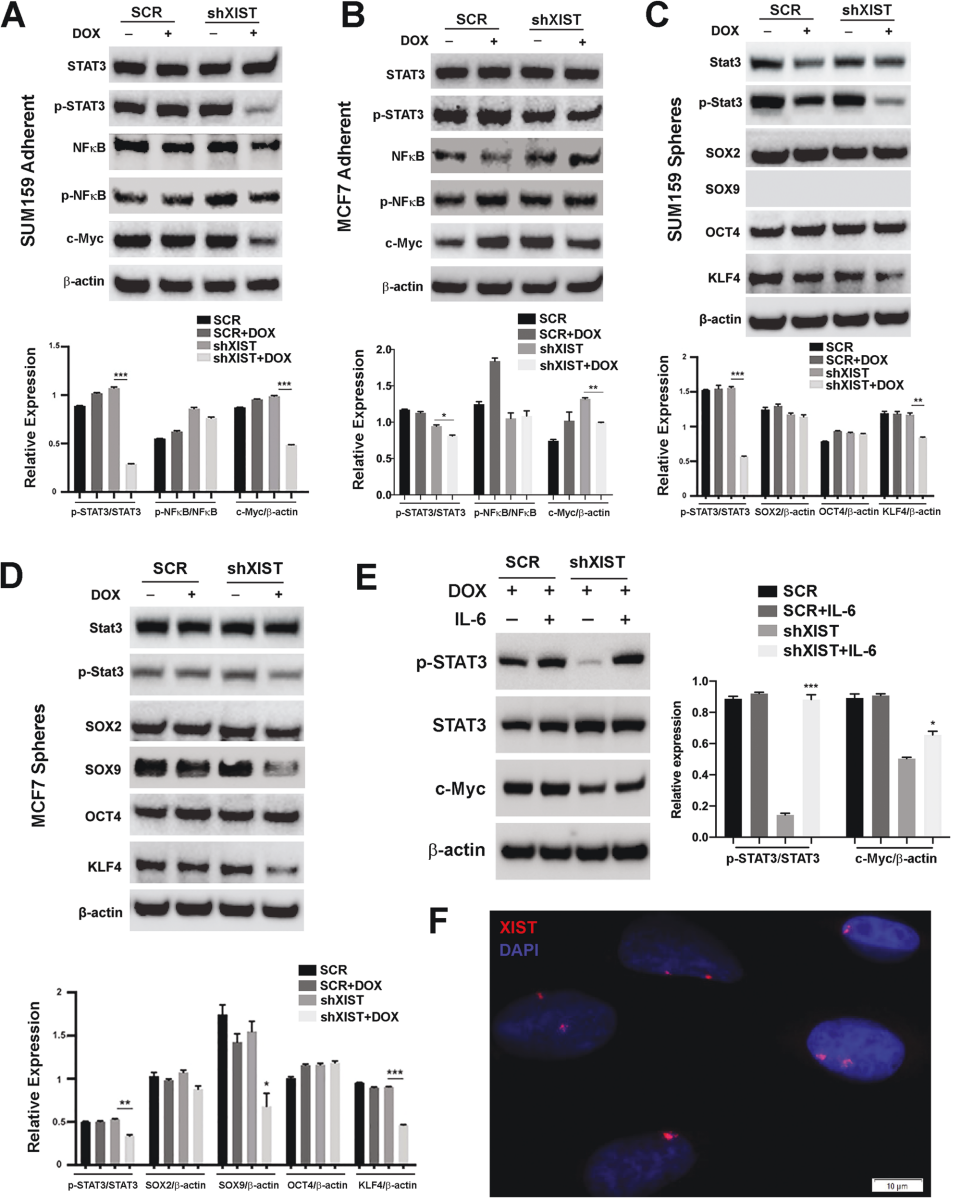
Aberrant *XIST* expression promotes IL-6/STAT3 signaling and expression of c-MYC, KLF4 and SOX9. **A**, **B** SUM159 (**A**) and MCF7 (**B**) BCCs expressing shXIST vs. a SCR sequence were treated with or without DOX (1 µg/ml) for 3 days under 2D adherent culture conditions and subjected to immunoblotting analysis to examine STAT3 and NFkb activation as well as c-MYC expression. Densitometry analysis was done with Image J to calculate the ratios of p-STAT3/STAT3, p-NFkb/NFkb, and c-MYC/β-actin. **C**, **D** Tumorsphere lysates derived from SUM159-shXIST (**C**) or MCF7-shXIST (**D**) BCCs treated with or without DOX (1 µg/ml) for 14 days were examined by immunoblotting with antibodies against STAT3 and p-STAT3 as well as CSC regulatory factors including SOX2, SOX9, OCT4, and KLF4, followed by densitometry analysis to determine the ratios of p-STAT3/STAT3 and SOX2, SOX9, OCT4 and KLF4 against β-actin respectively. **E** DOX-treated SUM159 BCCs expressing shXIST vs. SCR sequence were cultured in DOX-containing media supplemented with or without human IL-6 (50 ng/mL) for 3 days, and cell lysates were subjected to immunoblotting to examine STAT3 activation and C-MYC expression. **F** SUM159 BCCs grown on glass cover slips were subjected to RNA *FISH* using Stellaris® human *XIST FISH* probes to examine XIST subcellular localization. Bar: 10 µM. Data are presented as mean ± SD and statistical significance are determined by a two-tailed unpaired t-test. *p < 0.05, **p < 0.01, ***p < 0.001, ***p < 0.0001, respectively. All data shown are representative of three independent experiments.

To investigate how XIST-driven IL-6 production promotes ALDH^+^ CSCs, we next examined whether DOX-induced *XIST* KD in TN vs. luminal BCCs affects the activation of NFkB/STAT3 signaling pathways downstream of IL-6, leading to suppressed expression of key CSC regulatory proteins. Compared to SCR control cells, SUM159-shXIST BCCs treated with DOX for 3 days display markedly reduced phosphorylation of STAT3 at Tyr705 (p-STAT3), while total STAT3 protein expression is not significantly changed (Fig. 6A, upper panel), leading to significantly decreased p-STAT3/STAT3 ratio (Fig. 6A, lower panel). In contrast to markedly reduced STAT3 activation, the activation of p65 NFκB, indicated by the ratio of phospho-NFκB p65 (Ser536) to p65 NFκB, is not significantly affected following *XIST* KD. We also observed that c-MYC, a key CSC regulatory protein and transcriptional factor implicated in promoting tumor growth and cancer stemness, is markedly reduced in SUM159-shXIST but not SCR cells following DOX treatment (Fig. 6A). Similar studies were performed in MCF7 luminal BCCs (Fig. 6B), which indicated that DOX-induced *XIST* KD slightly (but significantly) reduces STAT3 activation (indicated by p-STAT3/STAT3 ratio) and c-MYC expression (indicated by c-MYC/β-Actin ratio). This reduced effect of *XIST* KD on p-STAT3 and c-MYC expression in MCF7 may reflect that fact that luminal BCCs (i.e., MCF7 and T47D) harbor very low proportion of ALDH^+^ CSCs (< 1%) as compared to TN BCCs (i.e., SUM159 and HCC70). These studies suggest that DOX-induced *XIST* KD decreases tumor growth and CSC activity by impairing STAT3 activation and c-MYC expression.

To further explore potential changes in the expression of other CSC-regulatory proteins following DOX induced *XIST* KD, we cultivated tumorspheres using SUM159-shXIST and MCF7-shXIST as well as corresponding SCR cells in the absence or presence of DOX for 14 days. Consistent with markedly reduced STAT3 activation in DOX-treated SUM159-shXIST cells under 2D adherent conditions (Fig. 6A), significantly reduced STAT3 activation was also observed in tumorsphere lysates derived from DOX-treated SUM159-shXIST (Fig. 6C) and MCF7-shXIST (Fig. 6D) cells. Further immunoblotting with specific antibodies against CSC regulatory factors including SOX2, SOX9, OCT4 and KLF4 revealed that KLF4 expression is consistently suppressed in DOX-treated SUM159-shXIST (Fig. 6C) and MCF7-shXIST (Fig. 6D) spheroid cells, whereas SOX2 and OCT4 expression are not significantly changed. We also found that SOX9, which is specifically expressed in luminal MCF7 but not mesenchymal SUM159 BCCs, is markedly suppressed in MCF7 tumorspheres upon DOX-induced *XIST* KD (Fig. 6D). Therefore, DOX-induced XIST KD in TN and luminal BCCs blunts IL-6 mediated STAT3 activation, leading to impaired expression of key CSC regulatory factors including c-MYC, KLF4 and SOX9.

To further determine the role of IL-6 in mediating the effect of *XIST* KD on STAT3 activation, we added exogenous IL-6 (50 ng/ml) to SUM159-shXIST and SCR cells that were pretreated with DOX for 3 days and examined whether IL-6 treatment for 3 days rescues the reduced STAT3 activation and C-MYC expression in SUM159 *XIST* KD cells. This revealed that exogenous IL-6 significantly rescued p-STAT3 and C-MYC expression in SUM159 BCCs subjected to *XIST* KD (Fig. 6E). In contrast to *XIST* KD cells, IL-6 treatment of SUM159-SCR cells pretreated with DOX failed to increase p-STAT3 and C-MYC expression significantly (Fig. 6E). This suggests that XIST-driven IL-6 production in SUM159-SCR cells sufficiently maintains STAT3 activation and downstream C-MYC expression.

In normal human mammary epithelial cells (HMEC), lncRNA XIST associates with Xi to form a nuclear cloud called Barr body, which is frequently lost in BC cells [24, 25]. To examine the subcellular localization of aberrant *XIST* expression in SUM159 BCCs, we employed RNA *FISH* (fluorescence in situ hybridization) on SUM159 BCCs using Stellaris® human *XIST FISH* probes. This revealed that SUM159 BCCs possess one or two *XIST* RNA domains localized in the nucleus (Fig. 6F). Thus, aberrant *XIST* expression in SUM159 BCCs may serve as nuclear sinks to sequester/antagonize tumor suppressive miRNAs such as let-7a-2-3p to regulate tumor proinflammatory (i.e., IL-6) signaling.

### *XIST* expression in ALDH^−^ bulk tumor cells drives paracrine IL-6 signaling to regulate ALDH^+^ CSCs

**Fig. 7:**
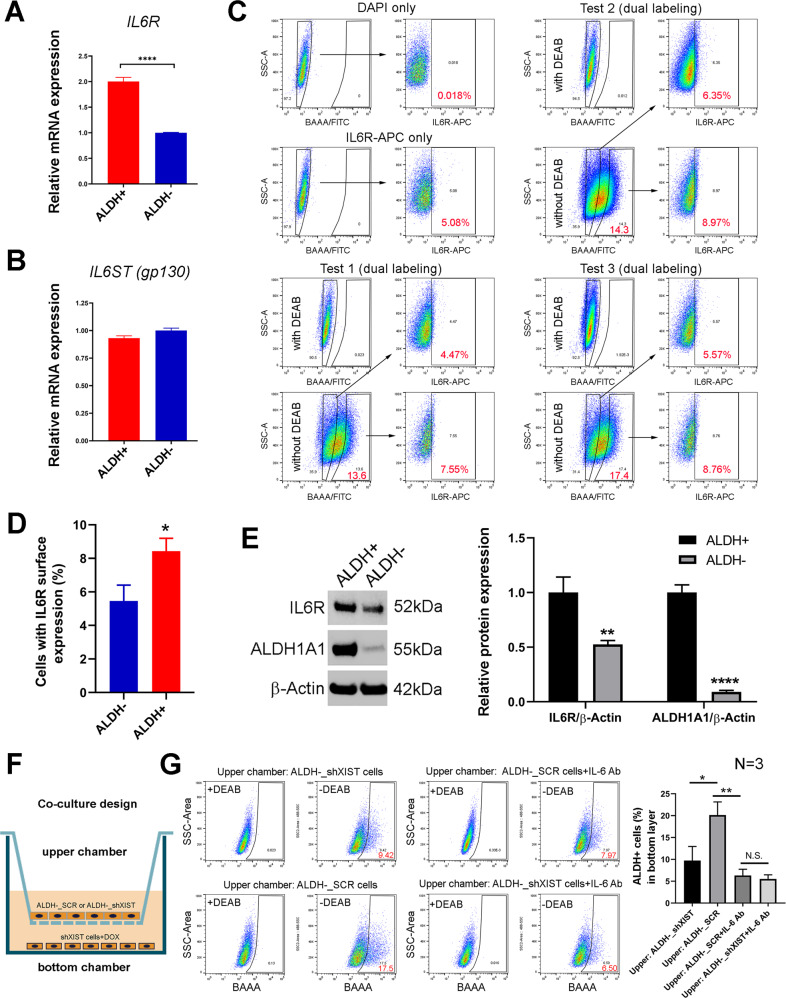
*XIST* expression in ALDH^−^ bulk tumor cells drives paracrine IL-6 signaling to promote ALDH^+^ CSCs. **A**, **B** SUM159 ALDH^−^ bulk tumor cells and ALDH^+^ CSCs were FACS sorted and subjected to qRT-PCR analysis (*n* = 3) to examine the relative expression of *IL6R* (**A**) and *IL6ST* (**B**). **C**, **D** SUM159 BCCs were labeled with APC-conjugated antibodies against human IL6R and then subjected to ALDEFLOUR assay and flow cytometry analysis as gated in (**C**) to determine the percentages of cells with cell surface IL6R expression between ALDH^−^ bulk tumor cell and ALDH^+^ CSC populations (**D**, *n* = 3). **E** SUM159 ALDH^−^ bulk tumor cells and ALDH^+^ CSCs were FACS sorted and subjected to immunoblotting with antibodies against IL6R, ALDH1A1 and β-actin, followed by densitometry analysis to determine the ratios of IL6R and ALDH1A1 expression against β-actin respectively (*n* = 2). **F**, **G** DOX-treated SUM159-shXIST cells were co-cultured with ALDH^−^ cells presorted from SUM159-shXIST or SCR cell lines in the lower and upper chambers respectively at the presence or absence of IL-6 neutralizing antibody (**F**), and SUM159-shXIST cells grown in the bottom chambers in the carrier plate were harvested and analyzed for ALDH^+^ cell content by ALDEFLUOR assay. Data were plotted based on the results of three biological repeats (*n* = 3). Statistical significance was determined by a two-tailed unpaired t-test. *p < 0.05, **p < 0.01, ****p < 0.0001, respectively. N.S.: no statistical significance.

Our RNAseq analyses indicated *IL-6* as the gene most significantly inhibited in ALDH^−^ bulk tumor cells upon *XIST* KD, although this gene is also downregulated in ALDH^+^ CSCs (Fig. 4B). In parallel with the most significant inhibition of *IL-6* expression in ALDH^−^ bulk tumor cells, miRNA array analysis unveiled that let-7a-2-3p is more robustly upregulated in ALDH^−^ BCCs vs. ALDH^+^ CSCs following *XIST* KD (Fig. 5B). Based on these findings we hypothesized that ALDH^+^ CSCs have preferential responses to IL-6 due to their elevated expression of IL-6 receptor (IL6R) as compared to bulk ALDH^−^ BCCs. To test this, we sorted ALDH^+^ CSCs and ALDH^−^ bulk BCCs from SUM159 and performed qRT-PCR analysis to determine their expression of *IL6R* and *IL6ST*, the latter of which encodes the IL-6 cytokine family signal transducer gp130. Indeed, ALDH^+^ CSCs express significantly higher levels of *IL6R* compared to ALDH^−^ BCCs (Fig. 7A). In contrast to *IL6R*, *IL6ST* expression is not significantly different between ALDH^−^ and ALDH^+^ BCCs (Fig. 7B). Using fluorophore-labeled antibodies, we further examined cell surface expression of IL6R between ALDH^+^ and ALDH^−^ BCCs (Fig. 7C). This revealed that, compared to ALDH^−^ BCCs, ALDH^+^ CSCs contain significantly higher proportion of cells expressing cell surface IL6R (Fig. 7D). This high IL6R expression of ALDH^+^ CSCs is further confirmed by immunoblotting with antibodies against human IL6R (Fig. 7E). Using antibody against human ALDH1A1, the major ALDH isoform expressed in SUM159 BCCs, we further confirmed that ALDH^+^ CSCs enriched by ALDEFLOUR assay express markedly higher level of ALDH1A1 relative to ALDH^−^ bulk BCCs (Fig. 7E).

Lastly, to investigate if XIST-driven IL-6 cytokine production from bulk ALDH^−^ BCCs promotes ALDH^+^ CSCs in a paracrine fashion, we employed ALDH^−^ BCCs sorted from SUM159-shXIST or SCR cell lines by flow cytometry to co-culture with *XIST* KD cells (SUM159-shXIST cells pretreated with DOX for 3 days, bottom chamber) in DOX-containing medium (Fig. 7F). After 4 days of co-culture with or without IL-6 neutralizing antibody, we harvested SUM159 *XIST* KD cells from the bottom chamber and measured the proportion of ALDH^+^ CSCs in each condition (Fig. 7G, left panels). This revealed that SUM159 *XIST* KD cells cocultured with ALDH^−^ BCCs from the SCR vs. shXIST cell line had significantly elevated proportion of ALDH^+^ CSCs (Fig. 7G, right panel). Interestingly, this increased proportion of ALDH^+^ CSCs was completely blocked by IL-6 neutralizing antibody, while addition of IL-6 neutralizing antibody to SUM159-shXIST cells cocultured with ALDH^−^ cells from the same cell line failed to significantly reduce the proportion of ALDH^+^ CSCs (Fig. 7G, right panel). This study strongly supports a model in which XIST-driven IL-6 cytokine production from ALDH^−^ bulk tumor cells promotes ALDH^+^ CSCs in a paracrine fashion.

**Fig. 8:**
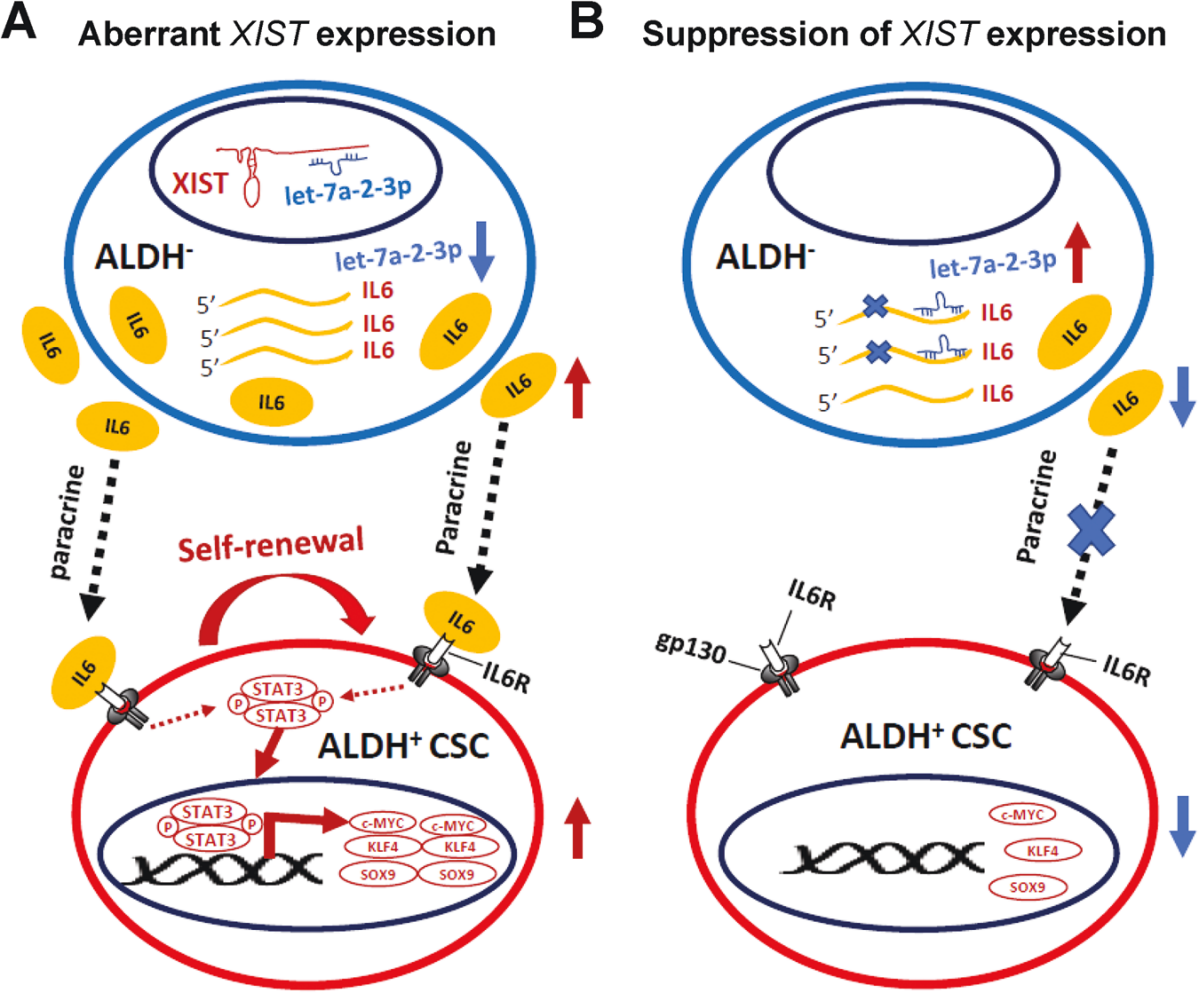
A model illustrating the mechanism of XIST in regulating ALDH^+^ CSCs. **A** Deregulated *XIST* expression in BCCs functions as a molecular sponge in the nucleus to sequester or antagonize let-7a-2-3p, which derepresses let-7a-2-3p mediated inhibition of *IL-6* expression from ALDH^−^ bulk tumor cells. This activated IL-6 cytokine production preferentially binds to IL6R highly expressed on ALDH^+^ CSCs, which in turn drives STAT3 activation and expression of key CSC factors including c-MYC, KLF4 and SOX9, promoting self-renewal of ALDH^+^ CSCs. **B** When aberrant *XIST* expression is suppressed in BCCs, XIST-mediated suppression of let-7a-2-3p is impaired, leading to decreased IL-6 production from ALDH^−^ bulk tumor cells and blockade of paracrine stimulation on ALDH^+^ CSCs.

Based on these findings, we present a diagram to illustrate the mechanism of XIST regulating ALDH^+^ CSCs in BC. In this model, aberrantly expressed XIST in ALDH^−^ bulk tumor cells sequesters or antagonizes let-7a-2-3p in the nucleus, blocking its repression of IL-6 protein expression. This XIST-driven production of IL-6 from ALDH^−^ bulk tumor cells preferentially binds to IL6R on ALDH^+^ CSCs to drive STAT3 activation and expression of key CSC factors (i.e., c-MYC, KLF4 and SOX9), promoting self-renewal of ALDH^+^ CSCs (Fig. 8A). In contrast, when aberrant *XIST* expression (from Xa) is suppressed in BCCs, XIST-driven IL-6 production and paracrine activation of ALDH^+^ CSCs are inhibited (Fig. 8B). Although IL-6 produced from ALDH^−^ bulk tumor cells promotes ALDH^+^ CSCs in a paracrine fashion, downregulation of *IL-6* (Fig. 4B) and upregulation of let-7a-2-3p (Fig. 5B) expression, despite to a lesser extent, were also detected in ALDH^+^ CSCs upon XIST KD. As let-7 miRNAs are mainly expressed in bulk tumor cells but not breast CSCs [34], we propose that XIST-driven IL-6 production from ALDH^−^ bulk tumor cells plays a major role in maintaining ALDH^+^ CSCs via paracrine IL-6 signaling.

## Discussion

As one of the best studied lncRNAs, XIST functions as a master regulator of XCI during early embryogenesis in female mammals. However, in post-XCI somatic tissues, dysregulation of XIST has recently been documented to play a role in chronic inflammatory diseases such as atherosclerosis [45], coronary artery disease [46], myocardial infarction [47], Alzheimer’s disease [48], Parkinson disease [49], among others. Aberrant expression of XIST also promotes inflammatory responses during tissue injury such as cerebral ischemia/reperfusion injury [50], lipopolysaccharide (LPS)-induced acute lung injury (ALI) [51], and sepsis-induced acute liver injury [52]. In parallel with dysregulated XIST expression in post-XCI somatic tissues, aberrant expression of XIST in post-XCI BCCs is a common phenomenon implicated in therapeutic resistance by regulating breast CSCs [30–32]. Despite previous studies demonstrating a link of aberrant XIST expression to inflammation and cancer stemness, the mechanisms involved remain elusive.

Using unbiased RNAseq analysis, we discovered that DOX-inducible *XIST* KD in SUM159 ALDH^−^ bulk tumor cells and ALDH^+^ CSCs most significantly altered the cytokine-cytokine receptor interaction pathways in each cell population, leading to pronounced suppression of proinflammatory cytokines IL-6 and IL-8. As IL-6 and IL-8 act as key regulators of tumor progression and CSC activity [16, 17, 19], we focused on IL-6 and IL-8 in this study to determine their roles in mediating XIST regulation of ALDH^+^ E-CSCs. Although both *IL-6* and *IL-8* gene expression were consistently downregulated in luminal MCF7 as well as SUM159 and HCC70 TN BCCs following DOX-induced *XIST* KD, we found IL-6, but not IL-8, plays a dominant role in mediating XIST regulation of ALDH^+^ E-CSCs. We further demonstrated that XIST promotes the proliferative, ALDH^+^ E-CSCs in luminal and TN BCCs by derepressing let-7 controlled paracrine IL-6/STAT3 signaling to increase the expression of CSC-associated factors including c-MYC, KLF4 and SOX9, promoting self-renewal of ALDH^+^ E-CSCs.

In addition to suppressing the expression of tumor supportive cytokines such as IL-6, IL-8, IL1A/B, LIF, G/M-CSF, DOX-induced KD of *XIST* also enhances the expression of tumor suppressive cytokines such as IL-7, IL-15, IL-18, etc., suggesting that XIST functions as a master regulator of cytokine-cytokine receptor interactions, leading to increased tumor growth and CSC activity. Future studies will be necessary to define the functional significance and underlying mechanisms of other tumor supportive cytokines/chemokines (i.e., IL1A/B, LIF, G/M-CSF and CXCL2/3) and inflammatory proteins (i.e., S100P and S100A9) in mediating XIST regulation of tumor growth and CSC activity. Similarly, the functional relevance and mechanisms of XIST in inhibiting tumor suppressive cytokine/chemokines (i.e., IL-7, IL-15, and IL-18) need to be determined.

In this study, we elucidated the mechanisms of XIST regulating IL-6 expression and ALDH^+^ CSCs, focusing on potential miRNAs targeted by XIST via its molecular sponge function. Through unbiased miRNA array analysis, we discovered that let-7a-2-3p, a member of let-7 tumor suppressor miRNAs known to repress IL-6 cytokine production [17, 33], is markedly upregulated in ALDH^−^ bulk tumor cells upon DOX-induced XIST KD. We systematically validated this upregulated let-7a-2-3p expression in SUM159, HCC70 and MCF7 BCCs following XIST KD, and subsequently verified the functional relevance of this upregulated let-7a-2-3p in suppressing *IL-6* gene expression and maintenance of ALDH^+^ CSCs. Lastly, we utilized luciferase reporter assay to define the regions of XIST that specifically interact with let-7a-2-3p, leading to activation of IL-6 protein expression.

In addition to acting as a major molecular sponge to antagonize many different regulatory ncRNAs, XIST also functions as a macromolecular scaffold for protein recruitment and acts as cis regulatory elements to modulate gene transcription, RNA splicing and post-transcriptional modification [53–56]. For instance, XIST mediates the recruitment of polycomb repressive complex (PRC) 1 and 2 to participate in XCI, leading to chromosome-wide gene silencing [57, 58]. In this study, we discovered that DOX-induced KD of XIST in HCC70 and SUM159 basal/mesenchymal, but not MCF7 luminal BCCs, significantly abrogated CD24^lo^CD44^hi^ M-CSCs by promoting CD24 epithelial marker expression (Fig. S2). However, this function of XIST in regulating CD24^lo^CD44^hi^ M-CSCs of TNBC is independent of its regulation of IL-6 cytokine expression via let-7a-2-3p. Future studies will be necessary to clarify if and how XIST functions to recruit PRC1/2 and other epigenetic modifiers to silence CD24 expression and maintain CD24^lo^CD44^hi^ M-CSCs in TNBC.

One intriguing phenomena for XIST is that its expression is markedly variable across different BC cells, even those derived from the same subtype (i.e., basal/luminal) of BC. This suggests that XIST expression in BC is regulated in a context-dependent manner. Intriguingly, it was reported that the Xi is lost in all examined BC cell lines regardless of *BRCA1* status, and that XIST in BCCs may be transcribed from the Xa, which exist in more than one copy [24]. Therefore, aberrant *XIST* expression in BCCs is likely to play functional roles totally unrelated to XCI. Indeed, by examining 816 X-lined protein coding genes, we found that DOX-induced *XIST* KD in SUM159 BCCs did not significantly upregulate the expression of most X-lined protein coding genes (Fig.S6E). As abnormal expression of *XIST* has been shown to associate with poor prognosis in patients with different cancer types, including BC [59, 60], pancreatic cancer [61], colorectal cancer [62] and brain cancer [63], this pathological roles of XIST in driving tumor growth and progression may relate to its multifaceted functions by acting as a molecular sponge to repress a number of tumor suppressor ncRNAs, and by recruiting epigenetic modifiers to alter chromatin structure and accessibility [21].

The mechanisms underlying dysregulation of XIST in BC and other malignancies remain elusive. Emerging evidence suggests that XIST and STAT3, by mutually regulating each other, form a double positive feedback loop that promotes inflammation and cancer development. XIST, by sponging/antagonizing miR-124, a STAT3 targeting miRNA, enhances STAT3 expression in retinoblastoma [64]. In LPS-induced ALI, XIST functions as a molecular sponge of miR-146a-5p positively regulating STAT3, which is then recruited to the promoter region of XIST to accelerate its transcription, thereby constituting a positive feedback loop that promotes inflammatory responses in ALI [51]. We suggest that this double positive feedback loop of XIST and STAT3 may contribute to the dysregulation of XIST in BC and other tumor types. This mutual regulation of XIST and STAT3 further supports a role of XIST in promoting cancer stemness.

In summary, our study unveiled a novel role of XIST in promoting ALDH^+^ CSCs in luminal and TN BC by antagonizing let-7a-2-3p in bulk tumor cells to enhance IL-6 production, which preferentially binds to IL6R on ALDH^+^ CSCs to drive STAT3 activation and c-MYC, KLF4 and SOX9 expression, promoting self-renewal of ALDH^+^ CSCs (Fig. 8). In addition to a universal role in promoting ALDH^+^ E-CSCs, XIST is also required to maintain CD24^-/lo^CD44^+/hi^ M-CSCs in basal/mesenchymal BCCs by inhibiting luminal differentiation, and this function is independent of its suppression of let-7a-2-3p. As DOX-induced *XIST* KD markedly abrogates tumor growth and CSC activities across different subtypes of BC, our study also identifies XIST as a potential therapeutic target for CSCs in BC and other malignancies.

## Materials and methods

### Cell culture

SUM159 and SUM149 BC cells were cultured in Ham’s F-12 (ThermoFisher Scientific) supplemented with 5% FBS (ThermoFisher Scientific), 5 µg/mL insulin (Sigma-Aldrich, St. Louis, MO), 1 µg/mL hydrocortisone (Sigma-Aldrich), and 1x antibiotic-antimycotic (ThermoFisher Scientific, 100x). MCF7 cells were grown in EMEM medium (ATCC) supplemented with 10% FBS, 1x antibiotic-antimycotic, and 10 µg/mL insulin (Sigma-Aldrich). BT20, MDA-MB-231, MDA-MB-468, MDA-MB-157 and SKBR3 were cultured in DMEM-high glucose (Gibco) supplemented with 10% FBS and 1x antibiotic-antimycotic. Vari068, HCC38, HCC70, HCC1937, HCC1954, T47D, ZR-75-1 and BT474 were maintained in RPMI1640 medium (ThermoFisher Scientific) supplemented with 10% FBS and 1x antibiotic-antimycotic. MCF10A is cultured in DMEM/F12 media (50:50, ThermoFisher Scientific) supplemented with 5% horse serum, 1x HEPES, 20 ng/ml EGF, 0.5 mg/ml hydrocortisone, 100 ng/ml cholera toxin, 10 µg/ml insulin and 1x antibiotic-antimycotic. All the cell lines are cultured at 37 °C under 5% CO_2_ in a humidified chamber and are mycoplasma-free.

### ALDEFLUOR assay, cell labeling and flow cytometry

The ALDEFLUOR™ (STEMCELL Technologies Inc, Vancouver, Canada) kit was used to detect aldehyde dehydrogenase (ALDH) enzymatic activity following manufacturer’s instructions. Briefly, single cells suspended at 1 × 10^6^ cells/mL in Aldefluor buffer are incubated with 5uL of BODIPY-aminoacetaldehyde (BAAA) for 40 min at 37 °C, and 5 uL of diethylaminobenzaldehyde (DEAB) was added along with BAAA as negative control. Aldefluor-labeled cells were resuspended in Aldefluor buffer containing 1 µg/mL of 4′, 6-diamidino-2-phenylindole (DAPI, Sigma) to discriminate live from dead cells. To detect M-CSC-like cells in DOX-treated MCF7, HCC70 and SUM159 BCCs expressing shXIST hairpin or SCR sequence, antibodies against human CD24 (BV421-conjugated, 1:50, BD Biosciences) and CD44 (APC-conjugated, 1:200, BD Biosciences) were used to label the cells in cold room for 30 minutes and then washed with 1xHBSS buffer supplemented with 2% FBS. To detect IL-6 receptor (IL6R) cell surface expression on ALDH^+^ vs. ALDH^−^ cells, SUM159 BCCs were first labelled with APC-conjugated antibodies against human IL6R (1:50, Biolegend) and subsequently labelled with ALDEFLUOR assay. A MoFlo Astrios Cell Sorter (Beckman Coulter) equipped with six lasers (354 nm, 405 nm, 488 nm, 561 nm, 594 nm, and 640 nm) and twenty-five fluorescent detectors was used for FACS analysis and sorting at the Flow Cytometry Core Facility of the University of Michigan.

### RNA extraction and quantitative Real-Time PCR (qRT-PCR) assays

Total RNA and miRNA were extracted using RNeasy and miRNeasy Mini kit (Qiagen) respectively. Stem-loop qRT-PCR analysis for miRNA expression was performed with Taqman miRNA assay using probe ID listed in Table S1. RNU24 was used as endogenous control to normalize miRNA expression. Thermal cycling conditions for Taqman miRNA assay include an enzyme activation step (95 °C for 10 min) and 40 cycles of amplification at 95 °C for 15 s followed by 60 °C for 1 min.

For mRNA/lncRNA expression, cDNA synthesis was performed with total RNA (10 ng-1 µg) using the High-Capacity cDNA Reverse Transcription Kit (ThermoFisher Scientific). cDNA samples were analyzed using TaqMan Universal Master Mix (for TaqMan probes) or Power SYBR® Green PCR Master Mix (for SYBR green primers) on an ABI PRISM 7900HT Real-Time PCR system (Applied Biosystems) according to manufacturer’s instructions. Commercial sources and sequences of primers used for qRT-PCR of mRNA/lncRNA expression are listed in Table S1. Gene expression of lncRNA/mRNAs was normalized to GAPDH.

### shRNA clones and lentiviral infection

DOX-inducible lentiviral shRNA clones against human XIST (GE Dharmacon, V2THS_92229, V3SH11258_245457769, V3SH11258_245651017, V3SH11258_ 245601352) or a scrambled sequence (SCR) were packaged at the University of Michigan Vector Core. SUM159, HCC70 and MCF7 BC cells were infected with lentiviruses in the presence of polybrene (8 μg/mL, Millipore) and the medium containing lentiviruses was replaced with fresh medium after 20 h of lentiviral infection. Puromycin (Invitrogen) selection was performed at a final concentration of 1.0 µg/mL (for MCF7) or 2.5 µg/mL (for HCC70 and SUM159) for 2 weeks to establish DOX-inducible shXIST or SCR cell lines.

### MTT assay

SCR and shXIST expressing cells were seeded at a density of 2000 cells per well in 96-well plates for overnight and cultured with or without DOX (1 µg/mL, Sigma-Aldrich) for 2, 4, or 6 days. After DOX treatment, MTT solution was added to each well and incubated for 3 h. After removing supernatant, cells in each well were solubilized by adding 150 µL of DMSO. OD absorbance of each condition at 590 nm was measured with a plate reader, and cell growth rate was plotted.

### 3D Soft agar assay

5% agarose gel (0.25 g Ultrapure agarose in 5 ml 1xHBSS) was melted in a microwave and cooled to 42 ˚C in water bath. 5 mL of 5% agarose gel was then mixed with 20 ml of prewarmed cell culture medium to make 1% agarose gel. The 1% agarose gel was diluted with cell culture medium at 1:1 ratio to make a 0.5% base gel, which was poured into 6-well plates at 2 mL per well. The 1% agarose gel was diluted with cell culture medium at 1:2 ratio to make a 0.33% agarose top gel, and 2 × 10^4^ pTripz-shXIST-MCF7 cells or 7.5 × 10^3^ pTripz-shXIST-SUM159 cells were mixed with 2 mL of top gel and poured on the top of the base gel per well into 6-well plates. Cells embedded in 0.33% agarose gel were incubated at 37 ˚C for 2–4 weeks and fed with 2 mL of completed medium per well containing with or without 1 µg/mL of DOX twice a week. Plates were stained with 0.005% Crystal Violet for 1 hour, and then washed with dH_2_O. Colonies were imaged with a dissection microscope and counted with ImageJ.

### Tumorsphere formation assay

SUM159 (10 cells/well) or MCF7 (20 cells/well) BCCs expressing shXIST or SCR sequence were sorted into 96-well ultra-low attachment plate (Corning) containing 120 µL/well of completed human MammoCult medium (StemCell Technologies) supplemented with DOX (1 µg/mL), 4 μg/mL heparin (StemCell Technologies), 1 μg/mL hydrocortisone (Sigma-Aldrich) and 1x antibiotic-antimycotic (Thermo Fisher Scientific) and cultured at 37 °C under 5% CO_2_ for 14 days. For tumorsphere rescue assays, SUM159/MCF7-shXIST or SCR cells cultured in completed MammoCult medium containing DOX (1 µg/mL) for 3 days in 96-well ultra-low attachment plates were supplemented with 50 ng/mL of exogenous IL-6 or IL-8 (BioLegend) and continued to cultivate for 11 days. Tumorspheres with diameter ≥ 40 µm were counted and photographed using an optical microscope with a 10x optical lens.

### Cell Co-culture assay

Co-culture of DOX-treated SUM159-shXIST cells with ALDH^−^ cells presorted from SUM159-shXIST or SCR cell lines in lower and upper chambers was conducted with Thermo Scientific™ Nunc™ cell culture inserts in carrier plate, 6 well format (Product code: 3491). Briefly, SUM159-shXIST cells pretreated with DOX (1 µg/mL) for 3 days were plated at 1 × 10^5^/well in 6-well carrier plate 2 h before placing the Nunc™ cell culture inserts loaded with 4 × 10^5^/well of ALDH^−^ cells sorted from SUM159-shXIST or SCR cells. After incubation with DOX (1 µg/mL) containing media supplemented with or without IL-6 neutralizing antibody (final concentration at 200 ng/mL, Proteintech, Catalog No. 69001-1-Ig) for 4 days, SUM159-shXIST cells in each well of the carrier plate were harvested and analyzed for ALDH^+^ cell content using ALDEFLUOR Assay.

### Western Immunoblotting

Total protein from cells or spheroids was extracted with 1xRIPA buffer (Cell Signaling Technology) supplemented with protease and phosphatase inhibitor cocktail (ThermoFisher Scientific). Cell lysates (20 ug per lane) were leaded and subjected to SDS-PAGE with Bolt^TM^ 4–12% Bis-Tris Plus Gel, blotted onto PVDF Membrane (ThermoFisher Scientific), and then blocked with 2% BSA in TBST buffer for 1 h before incubation with primary antibodies against STAT3 (9139 S), pSTAT3 (9145 S), NFκB p65 (8242 S), pNFκB p65 (3033 S), c-MYC (18583 S), SOX2 (3579 S), SOX9 (82630 S), OCT4 (2750 S) and KLF4 (12173 S), all from Cell Signaling Technology. IL-6 receptor (CD126), ALDH1A1, and β-actin were probed with antibodies obtained from Invitrogen (Catalog No. PA5-79506), BD Biosciences (Catalog No. 611194), and Sigma-Aldrich (A5316) respectively. Membrane was rinsed three times in TBST for 5 min each time and incubated with HRP-conjugated rabbit or mouse secondary antibodies (Cell Signaling Technology). Protein bands were visualized using WesternBright Sirius Chemiluminescent Detection Kit (Advansta).

### Next-generation RNA sequencing and gene expression profiling

ALDH^−^ and ALDH^+^ cells isolated from DOX-untreated SUM159-shXIST BCCs were treated with or without DOX (1 µg/mL) for 3 days, and total RNA from each cell sample was extracted and subjected to next generation RNA sequencing (RNAseq) at the University of Michigan DNA Sequencing Core. RNA abundance and integrity were determined by a Nanodrop-ND-1000 spectrophotometer (ThermoFisher Scientific) and an Agilent 2100 Bioanalyzer (Agilent Technologies), respectively. Only samples of total RNA with an RNA integrity number (RIN) > 9 were used for RNAseq. Sequencing read quality was assessed utilizing FastQC. Reads were aligned against the human reference genome (GRCh37) to generate spliced alignments. We conducted differential expression testing on the assigned read counts per gene utilizing edgeR. To reduce the dispersion of the dataset due to lowly expressed genes, genes with a mean aligned read count less than five across all samples were excluded from analysis. Normalized counts per million were estimated utilizing the “cpm” function in edgeR, and differences in expression of genes were estimated using the generalized linear modeling function glmLRT. Genes were considered differentially expressed between cell populations at a false discovery rate (FDR) with adjusted *p*-value < 0.05. Differences in gene expression and signaling pathways were visualized by volcano plotting and pathway interaction mapping was developed by iPathwayGuide (https://advaitabio.com/ipathwayguide/).

### MicroRNA array analysis

ALDH^−^ and ALDH^+^ cells isolated from DOX-untreated SUM159-shXIST cells were treated with or without (CTL) DOX at 1 µg/ml for 3 days, miRNAs from each sample were extracted using miRNeasy Mini kit (Qiagen) and analyzed by GeneChip™ miRNA 4.0 Array (ThermoFisher Scientific) according to manufacturer’s instructions. miRNA-containing total RNA (300 ng) was biotin-labeled using the FlashTag Biotin RNA Labeling kit (Afymetrix, USA) and hybridized in the GeneChip Hybridization Oven 640 (Affymetrix, USA) at 48 °C for overnight. After washed and stained in the GeneChip Fluidics Station 450 (Afymetrix, USA), arrays of different samples were scanned with a GeneChip Scanner 3000 7 G (Afymetrix, USA) and signal strength was evaluated using the Expression Console Software (EC) v1.2 (ThermoFisher Scientific). To identify differentially expressed miRNAs in ALDH^−^ and ALDH^+^ cells treated with or without DOX, acquired array data were analyzed using Multi Experiment Viewer (MeV v4.9.0; The Institute for Genomic Research) and miRNAs with an absolute value of fold change ≥ 0.6 were identified as potential miRNAs significantly changed upon DOX-induced XIST KD.

### RNA Fluorescence in Situ Hybridization (*FISH*) for *XIST*

Stellaris® human *XIST FISH* Probes were obtained from BIOSEARCH Technologies (Catalog No. SMF-2038-1) and *XIST FISH* experiment was conducted following supplier’s instructions. Briefly, SUM159 BC cells gown on glass coverslip were fixed in 3.7% formaldehyde in 1x PBS for 10 min at room temperature, and permeabilized in 70% ethanol for 1 hour at 4 °C. After incubation with wash buffer (final composition is 10% formamide in 2X SSC) for 5 min at room temperature, cells were incubated with Stellaris® *XIST FISH* Probes diluted in 1x hybridization buffer (final composition of 100 mg/mL dextran sulfate and 10% formamide in 2X SSC) for 16 h at 37 °C in the dark in a humidified chamber. Following hybridization, cells were incubated with wash buffer for 30 min, counterstained with DAPI for 30 min, and mounted with Vectashield® Mounting Medium (Vector Labs, catalog #: H1000).

### miRNA Mimic/Antagomir/DNA transfection and luciferase reporter assay

hsa-let-7a-2-3p mimic (ID: MC11174) vs. control (Cat#: 4464058), and miRNA antagomirs against human let-7a-2-3p (ID: AM11174), miR-374b-5p (ID: AM11339), miR-181c-5p (ID: AM10181), and miR1303 (ID: AM13799) vs. negative control (Cat#: AM17010) were obtained from ThermoFisher Scientific. Wild-type XIST cDNA fragments containing let-7a-2-3p binding sites 1 or 2 were generated by PCR from SUM159 BCC cDNAs, which is generated with the High-Capacity cDNA Reverse Transcription Kit (Life Technologies) and were inserted into the PmirGLO Dual-Luciferase miRNA Target Expression Vector (Promega, E1330). The mutagenesis of seed region in Site 2 was completed by overlap extension PCR. Overlap extension primer are (the underlined is mutant site of seed region): Overlap forward (F1) 5’-GAATAAAACTTTCTGTCGCAGCAGTATTGTCTCTACAAAATTC-3’; Overlap reverse (R1) 5’-GAATTTTGTAGAGACAATACTGCTGCGACAGAAAGTTTTATTC-3’. Sequences of the cDNA fragments containing WT site 1 and 2 as well as mutated site 2 are shown in Table S2. For luciferase reporter assay, SUM159 cells were seeded into 96-well plates overnight and then transfected with the luciferase reporter plasmid plus *let-7a-2* mimic or control using Lipofectamine 3000 (ThermoFisher Scientific). 48 h post transfection, luciferase activity was measured with the Dual-Luciferase Reporter Assay Kit (Promega, E2920). Firefly luciferase activity was normalized to Renilla to rule out the differences in transfection efficiency. Data were shown as fold change over control samples.

### Tumor growth in mammary Xenograft model of NOD/SCID mice

NOD/SCID mice were bred in-house and housed in pathogen-free rodent facilities at the University of Michigan. All supplies (cages, chow, and sterile water) were autoclaved, and all experiments were conducted according to standard protocol approved by the University Committee on the Use and Care of Animals. 5 × 10^5^ MCF7 or 5 × 10^4^ SUM159 cells were injected into the 4th mammary fat pads of 6-week-old female NOD/SCID mice. At the following day, mice were randomly selected to the Control or DOX cohort (*n* = 6). For mice transplanted with MCF7 cells, 17β-estradiol pellet (Cat# SE-121, 60-day release, 0.18 mg/pellet, Innovative Research of America) was implanted on the lateral side of neck between the ear and the shoulder of the mice on the day before tumor cell transplantation. Water containing DOX [2 mg/mL in 5% sucrose (w/v), Sigma-Aldrich] or Control (5% sucrose (w/v), Sigma-Aldrich) was administrated to each mouse cohort via bottled water supply at day 1 after tumor cell implantation. Tumor size was measured once a week with a caliper and calculated as tumor volume = Length × Width^2^/2.

### Bioluminescence imaging, tumor cell dissociation and limiting dilution transplantation

Tumor bearing mice in each cohort (*n* = 5) were anesthetized with isoflurane gas and given a single i.p. dose of 150 mg/kg D-luciferin (Promega) in PBS. For photon flux counting of bioluminescence, we used the IVIS Spectrum In Vivo Imaging system (PerkinElmer, Waltham, WA) coupled with a nosecone isoflurane delivery system. Results were analyzed using Living Image software provided with the IVIS imaging system. For dissociation of tumor cells grown as mouse xenografts, tumors were minced and digested with 1x collagenase/hyaluronidase (StemCell Technologies) in medium 199 (ThermoFisher Scientific), and cells were sieved sequentially through a 40 μm cell strainer (BD Falcon, USA) to obtain single cell suspension. Mouse cells were labeled with antibody against H2Kd (PE conjugated, 1:100, BD Biosciences) and gated out by flow cytometry to analyze ALDH^+^ CSCs in H2Kd^-^ tumor cells of human origin. For secondary transplantation, live (DAPI^-^) H2Kd^-^ SUM159 tumor cells sorted by FACS were prepared in 30% Matrigel (BD Biosciences) with 3 different dilutions (2500, 250, or 25 cells in 50 µl of volume for each site of injection) and injected bilaterally into the mammary fat pad (MFP) of 6-week-old female NOD/SCID mice (*n* = 3 for each dilution). Tumor appearance was monitored for 3 months and frequency of tumor initiating cells following transplantation was calculated using the ELDA software (Walter + Eliza Hall Bioinformatics, Institute of Medical research).

### Statistical analysis

For animal studies, power analysis was used to determine the minimum number of mice required to achieve robust and unbiased results for each study. All in vitro assays were repeated 2–3 times independently and representative data are shown with the number of biological repeats indicated in the figure legends. GraphPad Prism 8.0 was used to analyze and graph data, and Image J was used for image quantification. Results are plotted as the mean ± standard deviation (SD). To evaluate between group differences for continuous variables, two-tailed unpaired student’s t-test was used. To determine significant differences in studies with more than two groups, two-tailed one-way ANOVA was used. Estimation of variation within each group was conducted before comparation. The variance similar between groups was statistically compared. All *p*-values were two-sided and were considered statistically significant if *p*-value less than 0.05. No blind analysis was performed in this study.

## Supplementary information

Supplemental Materials

## Data Availability

RNAseq data are deposited in the NCBI Sequence Read Archive (SRA) with accession code PRJNA924707 and miRNA array data are deposited in the GEO database with accession code GSE222742. Other data and materials presented in this paper are available upon request.
