# Supplementary material for: LncRNA XIST regulates breast cancer stem cells by activating proinflammatory IL-6/STAT3 signaling
Source: Oncogene. 2023 Mar 15;42(18):1419–37. doi: 10.1038/s41388-023-02652-3 (PMC10154203; doi:10.1038/s41388-023-02652-3)
Supplement: Supplementary file 1 — Supplemental Materials [file 41388_2023_2652_MOESM1_ESM.pdf]

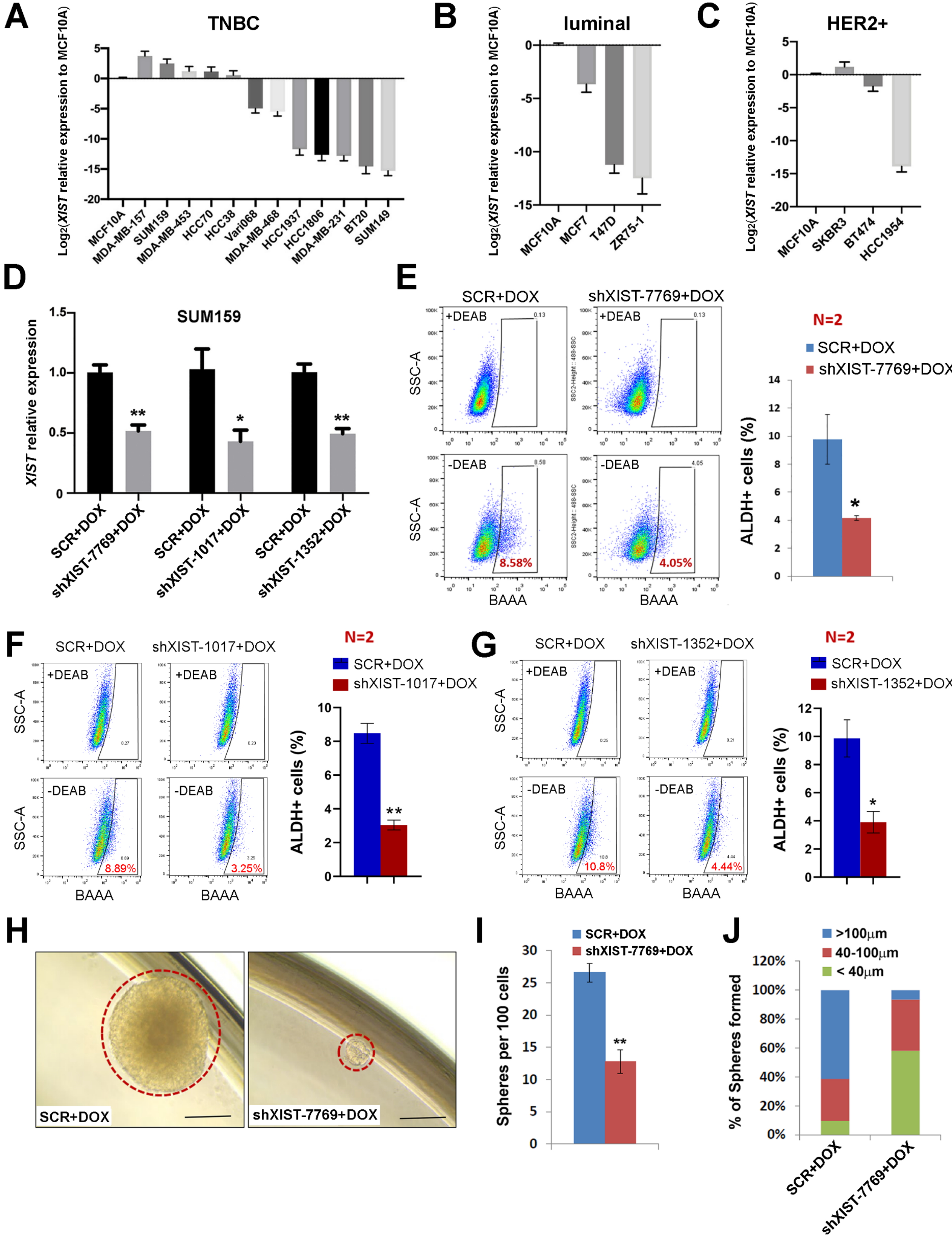
A
TNBC
Log2(XIST relative expression to MCF10A)
MCF10A
MDA-MB-157
SUM159
MDA-MB-453
HCC70
HCC38
Vari068
MDA-MB-468
HCC1937
HCC1806
MDA-MB-231
BT20
SUM149
B
luminal
MCF10A
MCF7
T47D
ZR75-1
C
HER2+
MCF10A
SKBR3
BT474
HCC1954
D
SUM159
XIST relative expression
SCR+DOX
shXIST-7769+DOX
shXIST-1017+DOX
shXIST-1352+DOX
E
SCR+DOX
shXIST-7769+DOX
+DEAB
-DEAB
SSC-A
BAAA
8.58%
4.05%
N=2
ALDH+ cells (%)
F
SCR+DOX
shXIST-1017+DOX
8.89%
3.25%
G
SCR+DOX
shXIST-1352+DOX
10.8%
4.44%
H
SCR+DOX
shXIST-7769+DOX
I
Spheres per 100 cells
J
>100μm
40-100μm
< 40μm
% of Spheres formed

Fig. S1. Relative expression of XIST in distinct subtypes of BCCs vs. non-tumorigenic breast epithelial cells and impact of DOX-induced *XIST* KD with different shXIST hairpins on ALDH$^+$ CSCs and tumorsphere formation. (A-C) Relative expression of XIST in TNBC (A), luminal (B) and HER2+ (C) BCCs relative to the non-tumorigenic breast epithelial cells MCF10A. (D) Validation of *XIST* knockdown efficiency in SUM159 BCCs expressing three additional DOX-inducible shXIST hairpins (shXIST-7769, shXIST-1017, shXIST-1352). (E-G) DOX-treated SUM159 cells expressing shXIST-7769 (E), shXIST-1017 (F) and shXIST-1352 (G) vs. SCR sequence exhibited significantly reduced proportion of ALDH$^+$ CSCs (N=2). (H-J) DOX-treated SUM159 cells expressing shXIST-7769 vs. SCR sequence exhibited impaired tumorsphere formation (H), exemplified by significantly decreased numbers of spheroids formed (I) and smaller spheroid sizes (J). *,**: $p<0.05$ or 0.01, respectively.

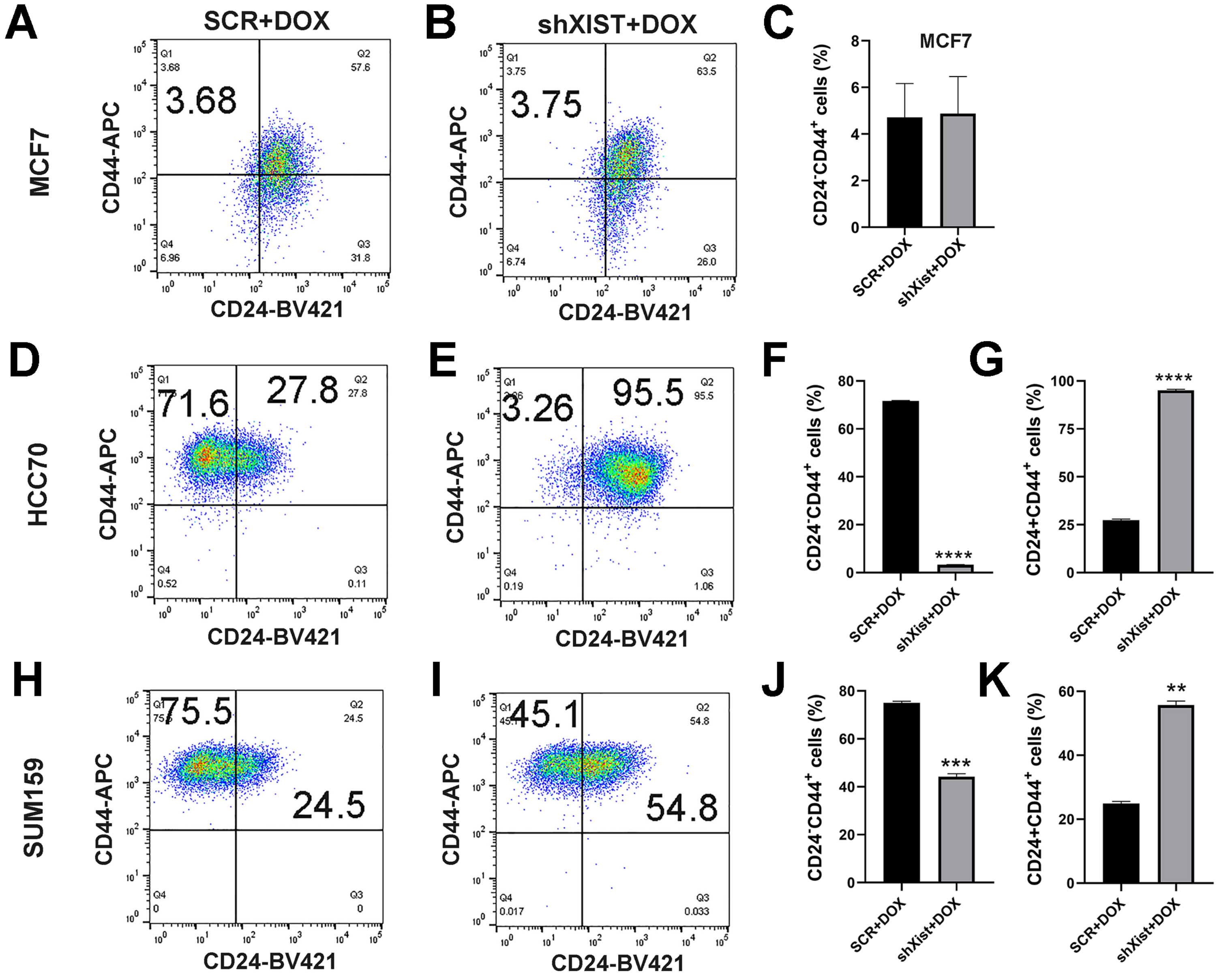

Fig. S2. Impact of DOX-induced *XIST* KD on $CD24^{-/lo}CD44^{+/hi}$ M-CSCs in luminal and TN BCCs. (A-C) Compared to DOX-treated cells expressing a SCR sequence, DOX-induced *XIST* KD in MCF7 luminal BCCs did not significantly affect the percentage of $CD24^{-/lo}CD44^{+/hi}$ M-like CSCs. (D-K) DOX-induced *XIST* KD in HCC70 (D-G) and SUM159 (H-K) TNBC cells significantly decreased the percentages of $CD24^{-/lo}CD44^{+/hi}$ M-CSC-like cells while increasing $CD24^{+}CD44^{+}$ cells that express luminal epithelial marker CD24. **,***,****: p<0.01, 0.001 and 0.0001, respectively.

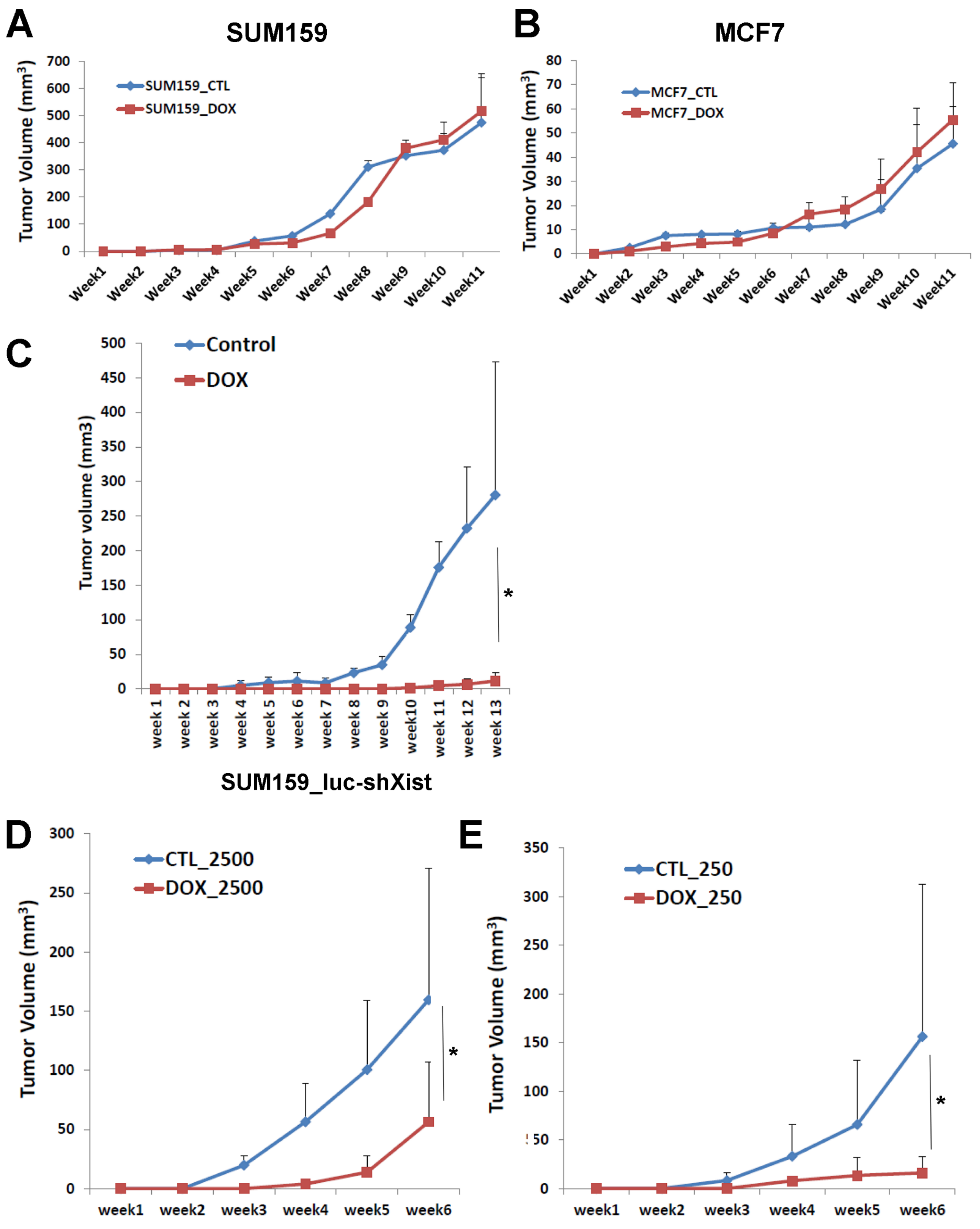

A
SUM159
Tumor Volume (mm³)
SUM159_CTL
SUM159_DOX
700
600
500
400
300
200
100
0
Week1
Week2
Week3
Week4
Week5
Week6
Week7
Week8
Week9
Week10
Week11
B
MCF7
Tumor Volume (mm³)
MCF7_CTL
MCF7_DOX
80
70
60
50
40
30
20
10
0
Week1
Week2
Week3
Week4
Week5
Week6
Week7
Week8
Week9
Week10
Week11
C
Control
DOX
Tumor volume (mm3)
500
450
400
350
300
250
200
150
100
50
0
week 1
week 2
week 3
week 4
week 5
week 6
week 7
week 8
week 9
week10
week 11
week 12
week 13
*
SUM159_luc-shXist
D
CTL_2500
DOX_2500
Tumor Volume (mm³)
300
250
200
150
100
50
0
week1
week2
week3
week4
week5
week6
*
E
CTL_250
DOX_250
Tumor Volume (mm³)
350
300
250
200
150
100
50
0
week1
week2
week3
week4
week5
week6
*

Fig. S3. Effects of DOX treatment on the growth of parental SUM159 and MCF7 BCCs, as well as DOX-induced *XIST* KD of SUM159 BCCs on primary and secondary tumor growth potential when implanted in MFPs of NOD/SCID mice. (A and B) DOX treatment did not significantly affect tumor growth of parental SUM159 (A) and MCF7 (B) BCCs injected to the #4 mammary fat pads of NOD/SCID mice. (C) Growth curve of SUM159-shXIST cells in NOD/SCID mice treated with or without DOX. (D and E) Growth curve of 2500 (D) or 250 (E) SUM159-shXIST_luc tumor cells dissociated from primary tumor xenografts of mice treated with or without DOX after limiting dilution transplantation in secondary NOD/SCID mice.

A

ALDH-_shXist vs ALDH-_CTL

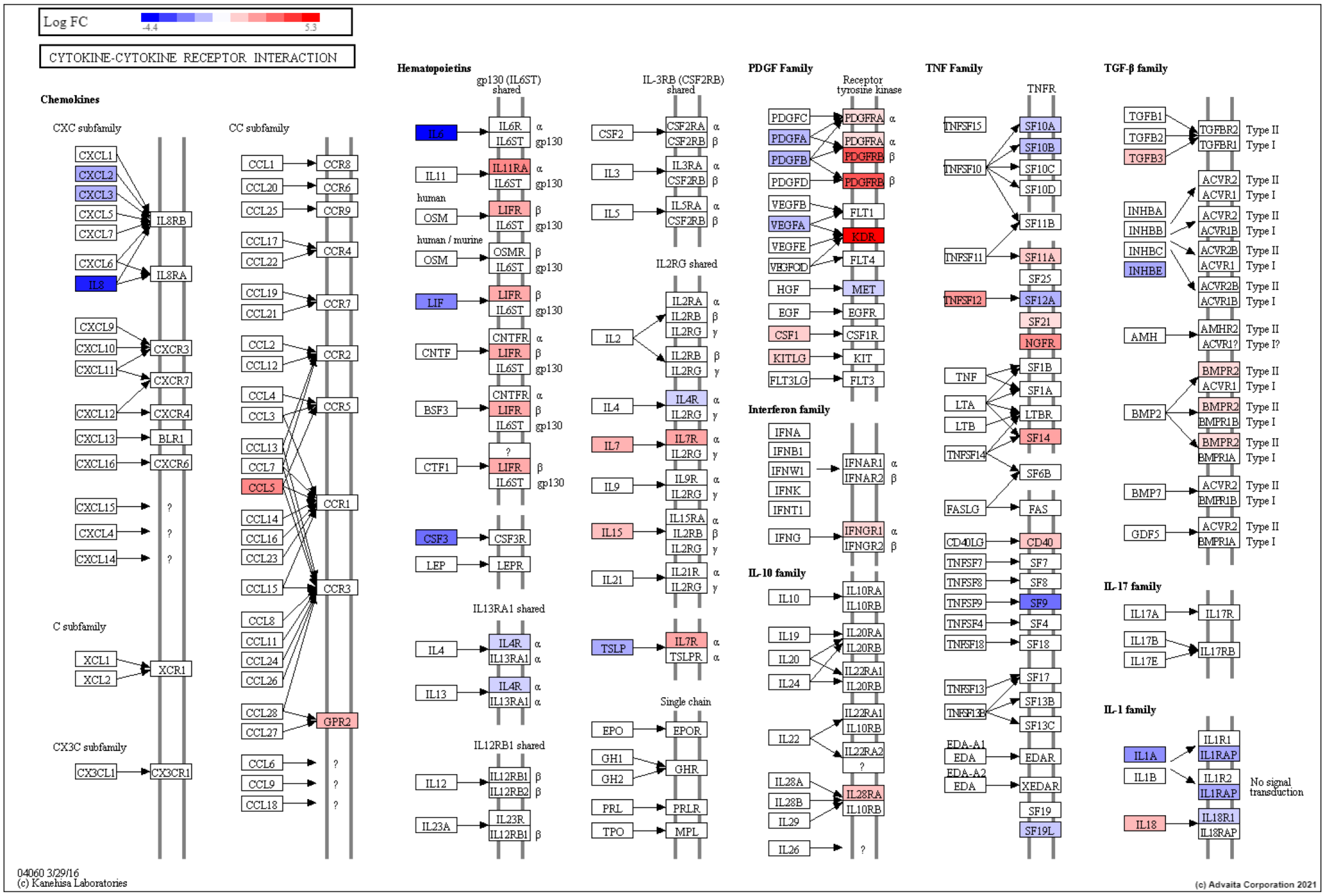

B

ALDH+_shXist vs ALDH+_CTL

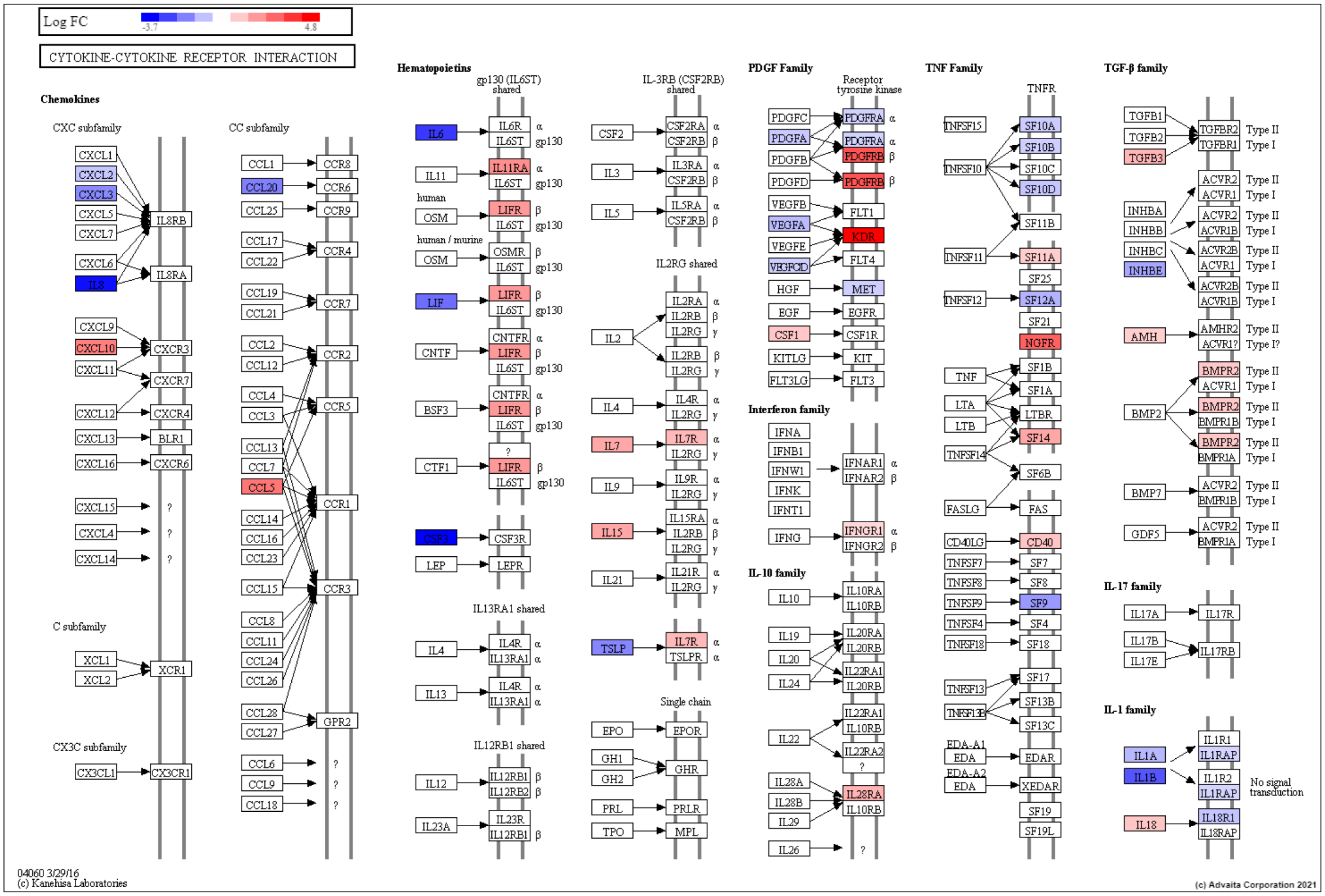

Fig. S4. XIST acts as a master regulator of cytokine-cytokine receptor interactions in breast cancer. (A and B) Heat map analysis and mapping of the significantly changed genes involved in cytokine-cytokine receptor interactions through the CXC and CC chemokine subfamilies, gp130 (IL6ST) or IL-3RB (CSFRB) shared hematopoietins, PDGF, TNF, and TGFβ families in SUM159 $ALDH^-$ (A) and $ALDH^+$ (B) cells upon DOX-induced XIST KD.

A

Pathway name	ALDH-_shXIST vs. ALDH-_CTL	ALDH+_shXIST vs. ALDH+_CTL
	PV	PV
Cytokine-cytokine receptor interaction	8.019e-8	3.953e-8
Chemical carcinogenesis	0.002	0.042
Neuroactive ligand-receptor interaction	0.005	0.003
Selenocompound metabolism	0.006	0.041
Malaria	0.010	0.036
Hippo signaling pathway -multiple species	0.011	0.004
Dorso-ventral axis formation	0.020	0.039
Wnt signaling pathway	0.021	0.018
Leishmaniasis	0.032	0.030
NOD-like receptor signaling pathway	0.033	0.010
Hematopoietic cell lineage	0.034	0.030
Non-alcoholic fatty liver disease (NAFLD)	0.037	0.039
Rheumatoid arthritis	0.040	7.387e-4

B

Gene symbol	ALDH-_shXIST vs. ALDH-_CTL		ALDH+_shXIST vs. ALDH+_CTL	
	LogFC	p-value	LogFC	p-value
S100P			-5.468	1.566e-4
S100A9			-4.839	0.043
LCN2			-4.425	0.006
PI3			-3.746	0.016
LIPG			-3.202	1.566e-4
CES1P2			-2.968	0.022
VEPH1			-2.953	0.007
IL1B			-2.489	1.566e-4
MEFV			-2.453	1.566e-4
SMTN			-1.910	1.566e-4
CCL20			-1.860	8.222e-4
CD14			-1.855	0.004
CA2			-1.798	3.011e-4
GNG11			-1.661	1.566e-4
SLPI			-1.637	1.566e-4
TMEM255B			-1.554	0.007
MYZAP			-1.546	0.044
FOXA2			-1.544	1.566e-4
GPSM3			-1.501	0.015
FLJ23867			-1.470	0.014
RFESD			-1.396	4.376e-4
PRSS3			-1.352	1.566e-4
HSD11B1			-1.285	0.005
MSC			-1.276	1.566e-4
ATP5EP2			-1.213	0.048

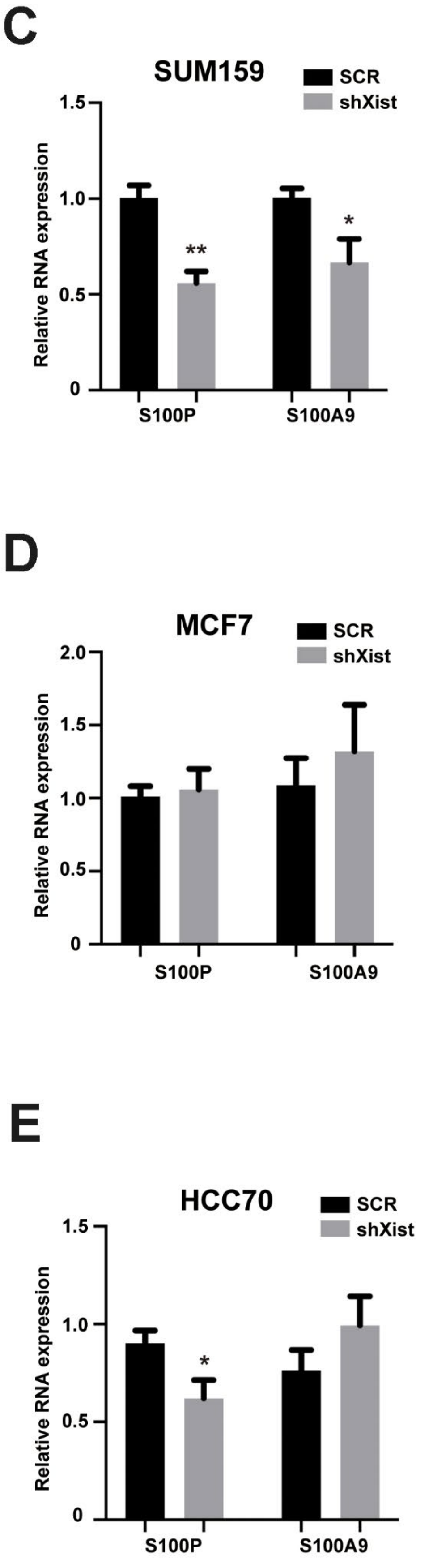

Fig. S5. *S100P* and *S100A9* as the most significantly downregulated genes in SUM159 ALDH$^+$ but not ALDH$^-$ BCCs upon DOX-induced *XIST* KD. (A) cytokine-cytokine receptor interaction remains as the most significantly changed pathway in both ALDH$^-$ and ALDH$^+$ cells upon *XIST* KD. (B) *S100P* and *S100A9* are the top two genes most significantly inhibited in ALDH$^+$ CSCs but not ALDH$^-$ bulk tumor cells upon *XIST* KD. (C-E) Downregulation of *S100P/A9* gene expression is found in SUM159 (C) but not MCF7 (D), while *S100P* but not S100A9 is inhibited in HCC70 (E) BCCs following DOX-induced *XIST* KD.

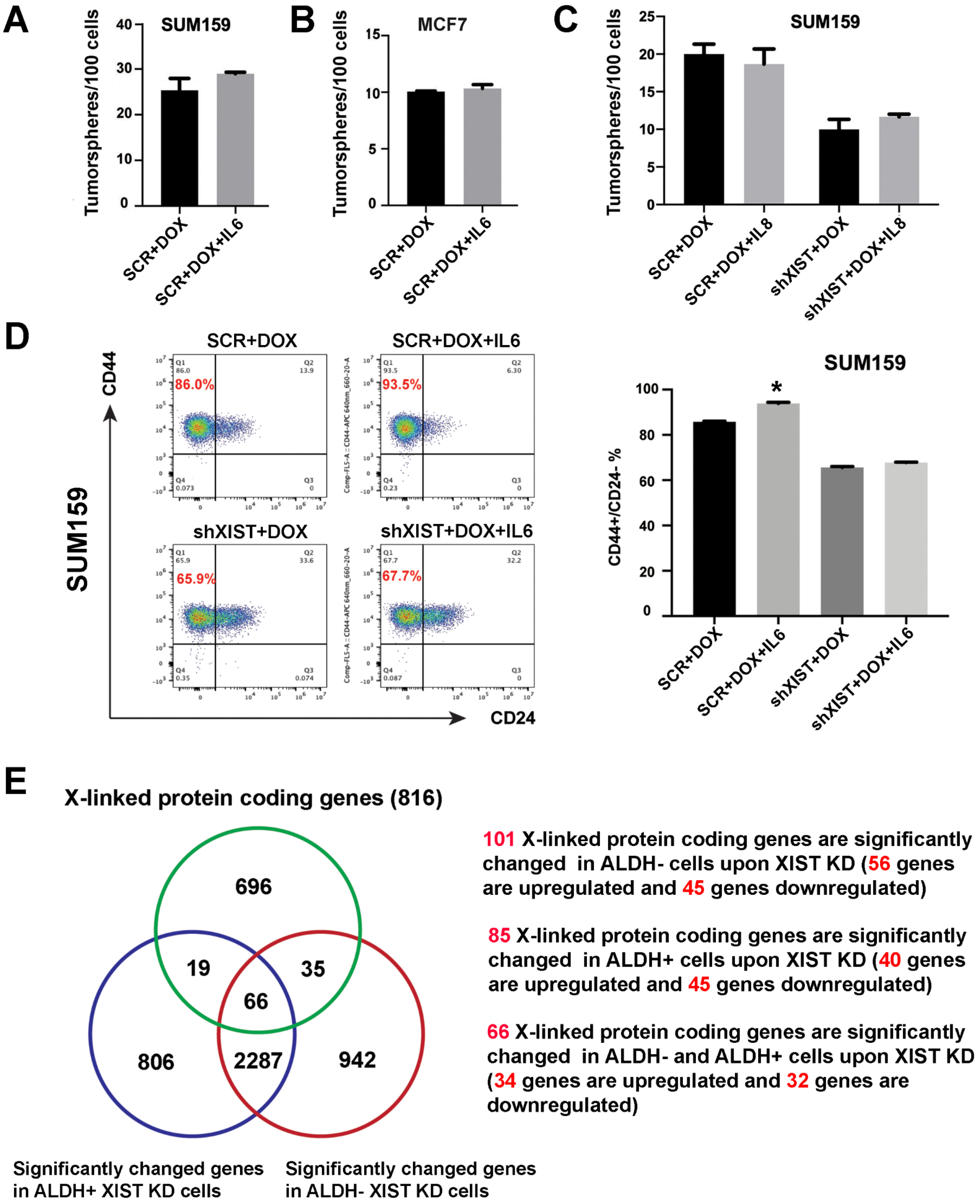
A
SUM159
Tumorspheres/100 cells
0
10
20
30
40
SCR+DOX
SCR+DOX+IL6
B
MCF7
Tumorspheres/100 cells
0
5
10
15
SCR+DOX
SCR+DOX+IL6
C
SUM159
Tumorspheres/100 cells
0
5
10
15
20
25
SCR+DOX
SCR+DOX+IL8
shXIST+DOX
shXIST+DOX+IL8
D
SUM159
CD44
CD24
SCR+DOX
86.0%
SCR+DOX+IL6
93.5%
shXIST+DOX
65.9%
shXIST+DOX+IL6
67.7%
SUM159
CD44+/CD24- %
0
20
40
60
80
100
*
SCR+DOX
SCR+DOX+IL6
shXIST+DOX
shXIST+DOX+IL6
E
X-linked protein coding genes (816)
696
19
35
66
806
2287
942
Significantly changed genes in ALDH+ XIST KD cells
Significantly changed genes in ALDH- XIST KD cells
101 X-linked protein coding genes are significantly changed in ALDH- cells upon XIST KD (56 genes are upregulated and 45 genes downregulated)
85 X-linked protein coding genes are significantly changed in ALDH+ cells upon XIST KD (40 genes are upregulated and 45 genes downregulated)
66 X-linked protein coding genes are significantly changed in ALDH- and ALDH+ cells upon XIST KD (34 genes are upregulated and 32 genes are downregulated)

Fig. S6. Roles of IL-6 vs. IL-8 in mediating XIST regulation of CSC activity and possibility of XIST in regulation of CSCs via its potential XCI function. (A and B) Supplementation of IL-6 at 50ng/ml had no significant impact on spheroid-forming capacity of DOX-treated SUM159 (A) and MCF7 (B) cells expressing a SCR sequence. (C) Addition of IL-8 at 50ng/ml failed to significantly rescue spheroid-forming capacity of SUM159 BCCs with XIST KD. (D) Supplementation of IL-6 to SUM159 BCCs with DOX-induced XIST KD failed to rescue the reduced $CD24^{lo}CD44^{hi}$ M-like CSCs. (E) Effects of DOX-induced *XIST* KD on the expression of 816 X-linked protein coding genes in SUM159 $ALDH^{-}$ and $ALDH^{+}$ cell populations.

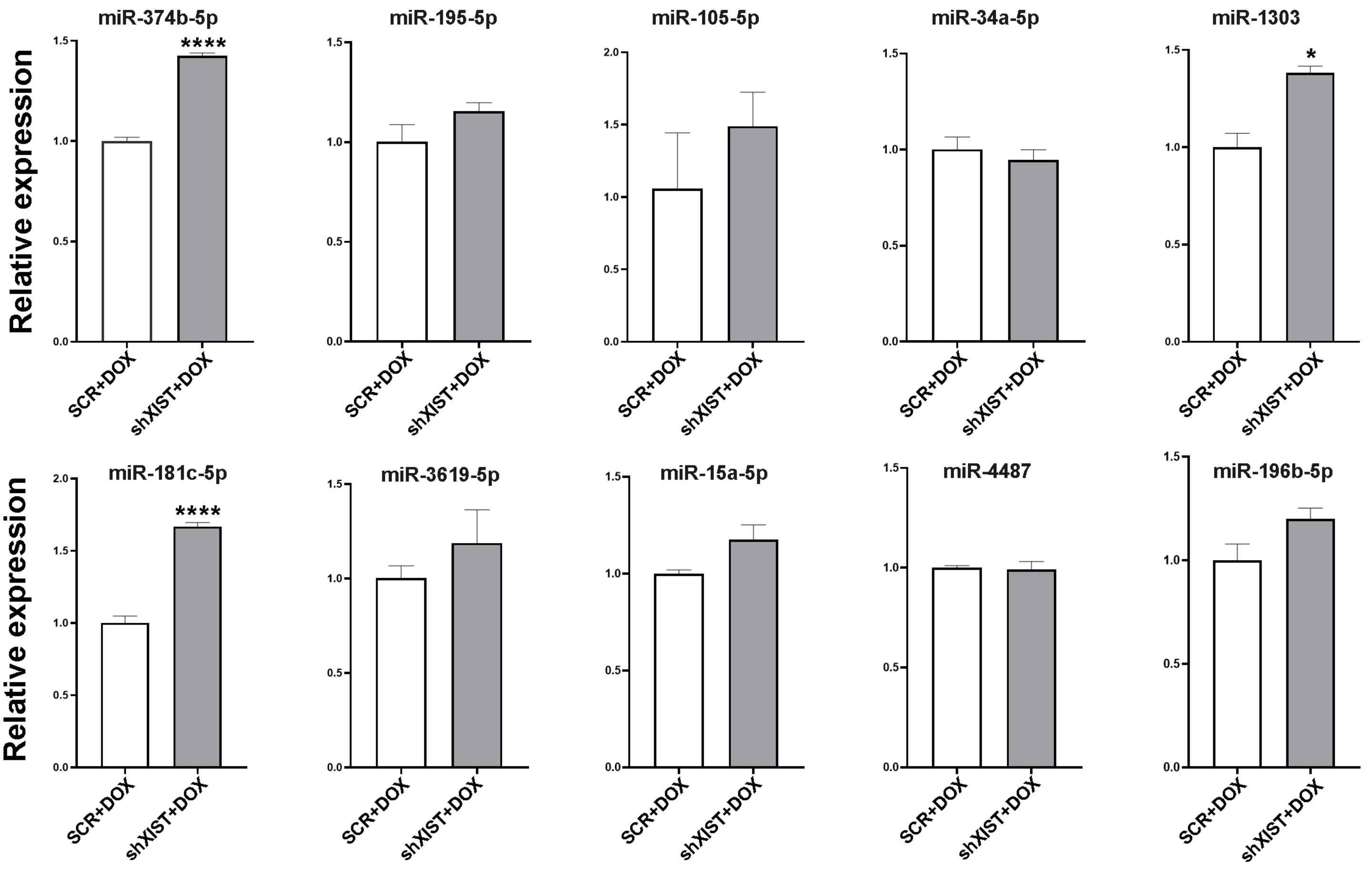

Fig. S7. qRT-PCR validation of 10 other upregulated miRNAs identified by miRNA array analysis in DOX-treated SUM159 BCCs expressing shXIST vs. a SCR sequence.

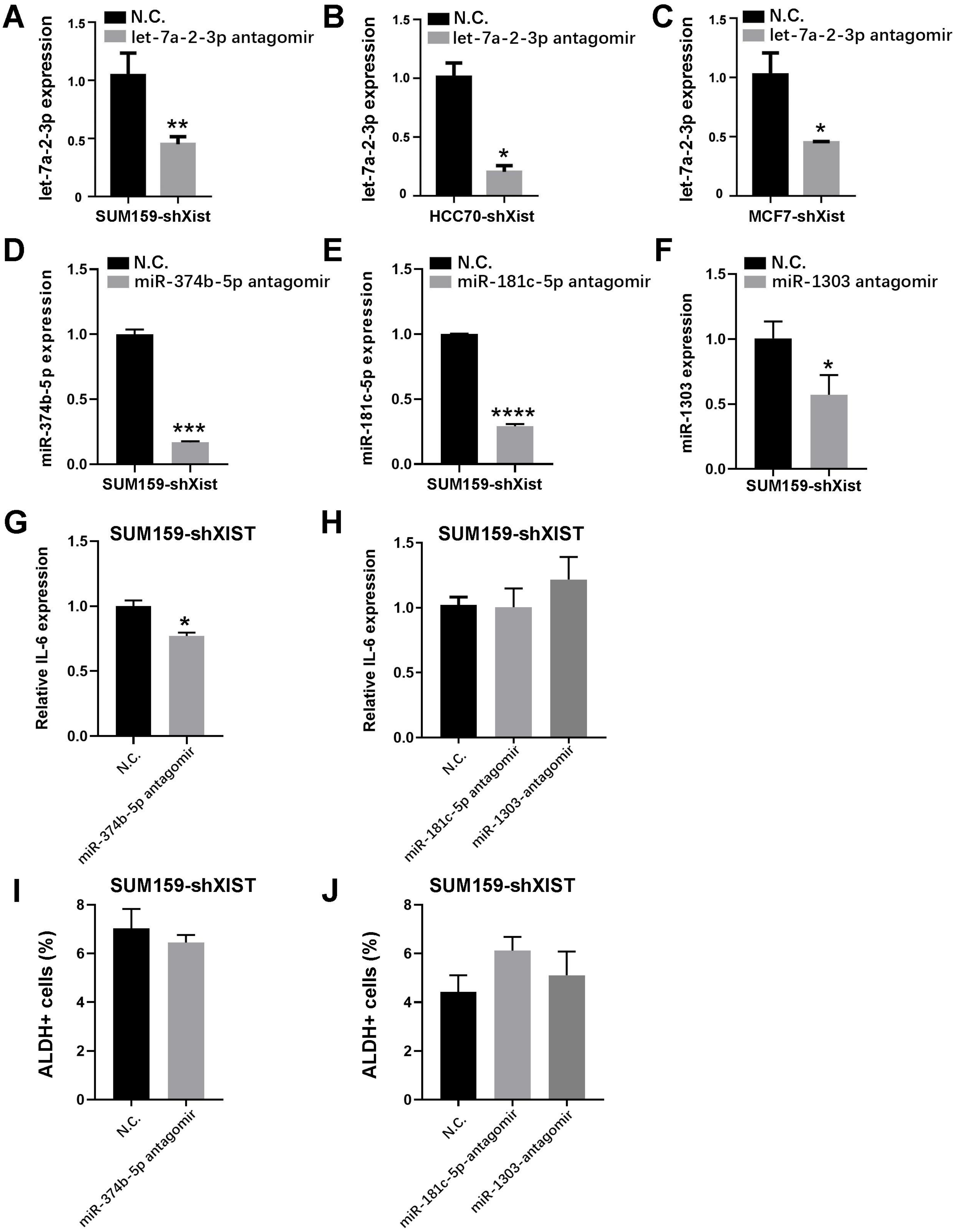
A
N.C.
let-7a-2-3p antagomir
let-7a-2-3p expression
**
SUM159-shXist
B
N.C.
let-7a-2-3p antagomir
let-7a-2-3p expression
*
HCC70-shXist
C
N.C.
let-7a-2-3p antagomir
let-7a-2-3p expression
*
MCF7-shXist
D
N.C.
miR-374b-5p antagomir
miR-374b-5p expression
***
SUM159-shXist
E
N.C.
miR-181c-5p antagomir
miR-181c-5p expression
****
SUM159-shXist
F
N.C.
miR-1303 antagomir
miR-1303 expression
*
SUM159-shXist
G
SUM159-shXIST
Relative IL-6 expression
*
N.C.
miR-374b-5p antagomir
H
SUM159-shXIST
Relative IL-6 expression
N.C.
miR-181c-5p antagomir
miR-1303-antagomir
I
SUM159-shXIST
ALDH+ cells (%)
N.C.
miR-374b-5p antagomir
J
SUM159-shXIST
ALDH+ cells (%)
N.C.
miR-181c-5p-antagomir
miR-1303-antagomir

Fig. S8. Efficiency of let-7a-23p antagomir in suppressing let-7a-23p expression and potential of miR-374b-5p, miR-181c-5p and miR-1303 in mediating XIST regulation of IL-6 expression and ALDH⁺ CSCs. (A-C) Introduction of a let-7a-2-3p antagomir but not negative control sequence (N.C.) into DOX-treated SUM159-shXIST (A), HCC70-shXIST (B) and MCF7-shXIST (C) BCCs significantly inhibited let-7a-2-3p expression in each cell line. (D-F) Introduction of miRNA antagomirs against miR-374b-5p (D), miR-181c-5p (E) or miR-1303 (F) but not N.C. into DOX-treated SUM159-shXIST BCCs significantly inhibited the expression of each corresponding miRNA. (G and H) Use of miRNA antagomirs against miR-374b-5p (G), miR-181c-5p or miR-1303 (H) vs. N.C failed to significantly increase *IL-6* gene expression in DOX-treated SUM159-shXIST BCCs. (I and J) Use of miRNA antagomirs against miR-374b-5p (I) and miR-181c-5p or miR-1303 (J) vs. N.C failed to significantly rescue the decreased proportion of ALDH⁺ CSCs in DOX-treated SUM159-shXIST BCCs.

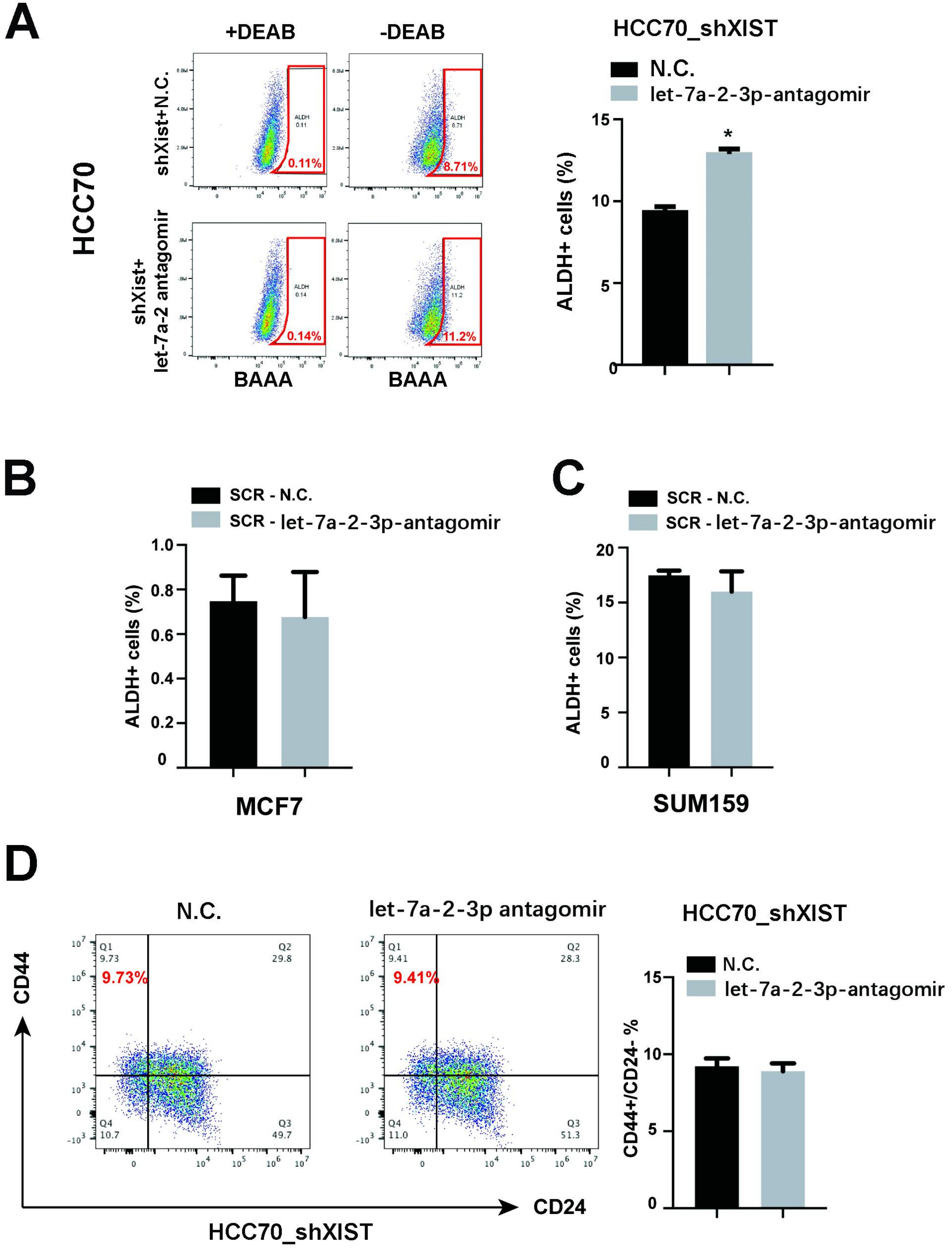
A
+DEAB
-DEAB
HCC70
shXist+N.C.
shXist+
let-7a-2 antagomir
0.11%
8.71%
0.14%
11.2%
BAAA
BAAA
HCC70_shXIST
N.C.
let-7a-2-3p-antagomir
ALDH+ cells (%)
*
B
SCR - N.C.
SCR - let-7a-2-3p-antagomir
ALDH+ cells (%)
MCF7
C
SCR - N.C.
SCR - let-7a-2-3p-antagomir
ALDH+ cells (%)
SUM159
D
N.C.
let-7a-2-3p antagomir
CD44
CD24
9.73%
9.41%
HCC70_shXIST
HCC70_shXIST
N.C.
let-7a-2-3p-antagomir
CD44+/CD24- %

Fig. S9. Potentials of let-7a-23p antagomir to rescue the reduced ALDH$^+$ E and CD24^{lo}CD44^{hi} M-CSCs in TN BCCs with XIST KD. (A) Introduction of a let-7a-2-3p antagomir but not N.C. into DOX-treated HCC70-shXIST BCCs significantly rescued the reduced proportion of ALDH$^+$ E-CSCs. (B and C) Introduction of a let-7a-2-3p antagomir into MCF7 (B) and SUM159 (C) BCCs expressing a SCR sequence failed to significantly increase ALDH$^+$ E-CSCs compared to the cells transfected with a N.C. RNA sequence. (E) Introduction of a let-7a-2-3p antagomir into HCC70 cells subjected to XIST KD failed to significantly rescue the reduced CD24^{lo}CD44^{hi} M-CSCs compared to XIST KD cells transfected with a N.C. RNA sequence.

Table S1. Information for the primers/probes used for qRT-PCR analysis

Assay name	Primer type	Assay ID	Vender	sequence 1 (5' to 3')	sequence 2 (5' to 3')
hsa-miR-374b-5p	Taqman	001319	ThermoFisher	na	na
hsa-let-7a-2-3p	Taqman	121214_mat	ThermoFisher	na	na
hsa-miR-195-5p	Taqman	000494	ThermoFisher	na	na
hsa-miR-105-5p	Taqman	002167	ThermoFisher	na	na
hsa-miR-34a-5p	Taqman	000426	ThermoFisher	na	na
hsa-miR-1303	Taqman	002792	ThermoFisher	na	na
hsa-miR-181c-5p	Taqman	000482	ThermoFisher	na	na
hsa-miR-3619-5p	Taqman	464898_mat	ThermoFisher	na	na
hsa-miR-15a-5p	Taqman	000389	ThermoFisher	na	na
hsa-miR-4487	Taqman	462492_mat	ThermoFisher	na	na
hsa-miR-196b-5p	Taqman	000496	ThermoFisher	na	na
RNU24	Taqman	001001	ThermoFisher	na	na
IL6	Taqman	Hs00985639_m1	ThermoFisher	na	na
IL6R	Taqman	Hs01075664_m1	ThermoFisher	na	na
IL6ST	Taqman	Hs00174360_m1	ThermoFisher	na	na
GAPDH	Taqman	Hs02758991_g1	ThermoFisher	na	na
IL8	SYBR Green	NM_000584	KiCqStart Primers	na	na
XIST	SYBR Green	na	IDT	ACCTACCGCTTTGGCAGAGA	GCCGAGTTATGCGGCAAGTC
S100P	SYBR Green	na	IDT	AAGGGGGAGCTCAAGGTGCTGA	ATCTGTGACATCTCCAGGGCATC
S100A9	SYBR Green	na	IDT	GCTGGAACGCAACATAGAG	GGTCCTCCATGATGTGTTC
GAPDH	SYBR Green	na	IDT	CCACTCCTCCACCTTTGACG	AGTGGTCGTTGAGGGCAATG

Table S2. XIST cDNA fragments harboring WT Site 1/2 and mutated Site 2

Cloned cDNA fragment containing XIST Site 1	GACTTCTCAGCATCATAGAAGGGGGTACTGATT TCCTAAAGTCTTTCTTGAATTTCTATTATGCAAA ATTGCCCTGAGGCCGGGTGTGGTGGCTCACAC CTGTAATCCCAGCACTTTGGGAGGCTGAGGTG GGAAGATCCCTTACTGCCAGGAGTTTGAGACCA GCCTGGCCAACATTAAAAAAAAAAAAAAGTAA GACAATTGCCCTGGAATCCCATCCCCCTCACACC TCCTTGGCAAAGCAGCAGGAGTGCTAACTAGCT AGTGCTTCTTCTCTTATACTGCTTAAATGCGCAT AATTAGCAGTAGTTGATGTGCCCCTATGTTAGA GTAGAATCCCGCTTCCTTGCTCCATTTGC
Cloned cDNA fragment containing XIST Site 2	GAGCTAGTATGAGGAAATGAAGTAATAGATGT GAGATCCAGACCGAAAGTCACTTAATTCAGCTT GCGAATGTGCTTTCTAAATTATAAAGCACTTGT AAATGAAAAATTTGATGCTTTCTGTATGAATAA AACTTTCTGTAAGCTAGGTATTGTCTCTACAAAA TTCTCATTGTATAGTTAAACCACAGTGAGAAGG GTTCTATAAGTAGTTATACAAACCAAGGGTTTA AATACCTGTTAAATAGATCAATTTTGATTGCCTA CTATGTGAACTCACTGTTAAAGGCACTGAAAAT TTATCATATTTCATTTAGCCACAGCC
Cloned cDNA fragment containing XIST Site 2 with mutation	GAGCTAGTATGAGGAAATGAAGTAATAGATGT GAGATCCAGACCGAAAGTCACTTAATTCAGCTT GCGAATGTGCTTTCTAAATTATAAAGCACTTGT AAATGAAAAATTTGATGCTTTCTGTATGAATAA AACTTTCTGTCGCAGCAGTATTGTCTCTACAAAA TTCTCATTGTATAGTTAAACCACAGTGAGAAGG GTTCTATAAGTAGTTATACAAACCAAGGGTTTA AATACCTGTTAAATAGATCAATTTTGATTGCCTA CTATGTGAACTCACTGTTAAAGGCACTGAAAAT TTATCATATTTCATTTAGCCACAGCC

Table S3. X-linked protein coding genes

	Gene name	X chromosome location	Gene location
1	ABCB7	Xq13.3	75051048..75156283
2	ABCD1	Xq28	153724856..153744755
3	ACE2	Xp22.2	15518197..15607211
4	ACOT9	Xp22.11	23701055..23743276
5	ACSL4	Xq23	109641335..109733257
6	ACTRT1	Xq25	128050962..128052403
7	ADGRG2	Xp22.13	18989307..19122956
8	ADGRG4	Xq26.3	136300963..136416890
9	AFF2	Xq28	148500617..149000663
10	AGTR2	Xq23	116170744..116174974
11	AIFM1	Xq26.1	130129362..130165841
12	AKAP14	Xq24	119895893..119920716
13	AKAP17A	X;Y	X:1591604..1602520;Y:1591604..1602520
14	AKAP4	Xp11.22	50190777..50201007
15	ALAS2	Xp11.21	55009055..55030977
16	ALG13	Xq23	111681170..111760649
17	AMELX	Xp22.2	11293413..11309588
18	AMER1	Xq11.2	64185117..64205708
19	AMMECR1	Xq23	110194186..110440233
20	AMOT	Xq23	112774877..112840831
21	ANOS1	Xp22.31	8528874..8732137
22	AP1S2	Xp22.2	15825806..15854813
23	APEX2	Xp11.21	55000363..55009057
24	APLN	Xq26.1	129645259..129654956
25	APOO	Xp22.11	23833353..23907938
26	APOOL	Xq21.1	85003877..85093315
27	AR	Xq12	67544021..67730619
28	ARAF	Xp11.3	47561205..47571908
29	ARHGAP36	Xq26.1	131058346..131089885
30	ARHGAP4	Xq28	153907378..153926264
31	ARHGAP6	Xp22.2	11137544..11665920
32	ARHGEF6	Xq26.3	136665550..136780932
33	ARHGEF9	Xq11.1	63634967..63785214
34	ARL13A	Xq22.1	100969708..100990831
35	ARMCX1	Xq22.1	101550547..101554700
36	ARMCX2	Xq22.1	101655281..101659850
37	ARMCX3	Xq22.1	101623151..101627843
38	ARMCX4	Xq22.1	101418278..101535988
39	ARMCX5	Xq22.1	102599206..102604159

40	ARMCX6	Xq22.1	101615125..101618000
41	ARR3	Xq13.1	70268334..70281883
42	ARSD	Xp22.33	2903972..2929339
43	ARSF	Xp22.33	3040234..3112727
44	ARSH	Xp22.33	3006546..3034111
45	ARSL	Xp22.33	2934521..2968245
46	ARX	Xp21.3	25003694..25015965
47	ASB11	Xp22.2	15281697..15315640
48	ASB12	Xq11.2	64224194..64230607
49	ASB9	Xp22.2	15243987..15270467
50	ASMT	X;Y	X:1595455..1643081;Y:1595455..1643081
51	ASMTL	X;Y	X:1403139..1453756;Y:1403139..1453756
52	ATG4A	Xq22.3	108088829..108154671
53	ATP11C	Xq27.1	139726348..139936903
54	ATP1B4	Xq24	120362089..120383237
55	ATP2B3	Xq28	153517642..153582929
56	ATP6AP1	Xq28	154428677..154436516
57	ATP6AP2	Xp11.4	40580970..40606848
58	ATP7A	Xq21.1	77910693..78050395
59	ATRX	Xq21.1	77504880..77786216
60	ATXN3L	Xp22.2	13318647..13320053
61	AVPR2	Xq28	153902625..153907166
62	AWAT1	Xq13.1	70234655..70240659
63	AWAT2	Xq13.1	70040542..70049938
64	BCAP31	Xq28	153700492..153724387
65	BCLAF3	Xp22.12	19912860..19991061
66	BCOR	Xp11.4	40051246..40177329
67	BCORL1	Xq26.1	129980313..130058071
68	BEND2	Xp22.13	18162931..18220886
69	BEX1	Xq22	103062651..103064171
70	BEX2	Xq22.2	103309346..103310990
71	BEX3	Xq22.2	103376323..103378164
72	BEX4	Xq22.1	103215092..103217246
73	BEX5	Xq22.1	102153712..102155977
74	BGN	Xq28	153494980..153509546
75	BMP15	Xp11.22	50910735..50916641
76	BMX	Xp22.2	15500807..15556519
77	BRCC3	Xq28	155071508..155123077
78	BRS3	Xq26.3	136487947..136493780
79	BRWD3	Xq21.1	80669503..80809877
80	BTK	Xq22.1	101349450..101390796

81	C1GALT1C1	Xq24	120625674..120630054
82	CA5B	Xp22.2	15738270..15788411
83	CACNA1F	Xp11.23	49205063..49233340
84	CAPN6	Xq23	111245099..111270483
85	CASK	Xp11.4	41514934..41923554
86	CBLL2	Xp22.11	22272913..22274461
87	CCDC120	Xp11.23	49053572..49069858
88	CCDC160	Xq26.2	134237182..134246842
89	CCDC22	Xp11.23	49235470..49250520
90	CCNB3	Xp11.22	50202713..50351914
91	CCNQ	Xq28	153587925..153599139
92	CD40LG	Xq26.3	136648158..136660390
93	CD99	X;Y	X:2691295..2741309;Y:2691295..2741309
94	CD99L2	Xq28	150766336..150898668
95	CDK16	Xp11.3	47218303..47229997
96	CDKL5	Xp22.13	18425608..18653629
97	CDX4	Xq13.2	73447053..73455171
98	CENPI	Xq22.1	101098204..101181859
99	CENPVL1	Xp11.22	51710512..51712131
100	CENPVL2	Xp11.22	51681212..51682831
101	CENPVL3	Xp11.22	51617020..51618912
102	CETN2	Xq28	152826994..152830757
103	CFAP47	Xp21.1	35919734..36385317
104	CFP	Xp11.23	47623282..47630305
105	CHIC1	Xq13.2	73563148..73687111
106	CHM	Xq21.2	85861180..86047558
107	CHRDL1	Xq23	110673856..110795817
108	CHST7	Xp11.3	46573765..46598496
109	CITED1	Xq13.1	72301646..72307157
110	CLCN4	Xp22.2	10156975..10237660
111	CLCN5	Xp11.23	49922596..50099230
112	CLDN2	Xq22.3	106900164..106930861
113	CLDN34	Xp22.2	9967358..9968352
114	CLIC2	Xq28	155276211..155334614
115	CLTRN	Xp22.2	15627318..15675644
116	CMC4	Xq28	155061622..155071136
117	CNGA2	Xq28	151734746..151745564
118	CNKSR2	Xp22.12	21374418..21654689
119	COL4A5	Xq22.3	108439838..108697545
120	COL4A6	Xq22.3	108155614..108439458
121	COX7B	Xq21.1	77899468..77907376

122	CPXCR1	Xq21.31	88747225..88754781
123	CRLF2	X;Y	X:1190490..1212649;Y:1190490..1212649
124	CSAG1	Xq28	152727484..152733721
125	CSF2RA	X;Y	X:1268814..1325218;Y:1268814..1325218
126	CSTF2	Xq22.1	100820391..100841520
127	CT45A1	Xq26.3	135708398..135723228
128	CT45A10	Xq26.3	135881065..135893444
129	CT45A2	Xq26.3	135811668..135820066
130	CT45A3	Xq26.3	135760044..135768191
131	CT45A5	Xq26.3	135775852..135785503
132	CT45A6	Xq26.3	135794685..135802787
133	CT45A7	Xq26.3	135829229..135837319
134	CT45A8	Xq26.3	135846497..135854592
135	CT45A9	Xq26.3	135863418..135871812
136	CT47A1	Xq24	120982476..120985760
137	CT47A10	Xq24	120938701..120942023
138	CT47A11	Xq24	120933840..120937158
139	CT47A12	Xq24	120877490..120932301
140	CT47A2	Xq24	120977606..120980928
141	CT47A3	Xq24	120972746..120976068
142	CT47A4	Xq24	120967886..120971208
143	CT47A5	Xq24	120963026..120966348
144	CT47A6	Xq24	120958165..120961487
145	CT47A7	Xq24	120953282..120956600
146	CT47A8	Xq24	120948422..120951744
147	CT47A9	Xq24	120943561..120946883
148	CT47B1	Xq24	120872607..120875866
149	CT55	Xq26.3	135156536..135171827
150	CT83	Xq23	116461686..116462976
151	CTAG1A	Xq28	154585133..154586816
152	CTAG1B	Xq28	154617609..154619282
153	CTAG2	Xq28	154651977..154653579
154	CTPS2	Xp22.2	16587999..16712910
155	CUL4B	Xq24	120523858..120575532
156	CXCR3	Xq13.1	71615919..71618511
157	CXorf38	Xp11.4	40626921..40647561
158	CXorf49	Xq13.1	71714371..71718285
159	CXorf49B	Xq13.1	71763349..71767204
160	CXorf51A	Xq27.3	146814106..146814744
161	CXorf51B	Xq27.3	146809771..146810411
162	CXorf58	Xp22.11	23907932..23939509

163	CXorf65	Xq13.1	71103889..71106740
164	CXorf66	Xq27.1	139955728..139965521
165	CYBB	Xp21.1-p11.4	37780059..37813461
166	CYLC1	Xq21.1	83861146..83886698
167	CYSLTR1	Xq21.1	78271468..78327611
168	DACH2	Xq21.1	86148451..86832602
169	DCAF12L1	Xq25	126549383..126552814
170	DCAF12L2	Xq25	126163499..126166289
171	DCAF8L1	Xp21.3	27977992..27981449
172	DCAF8L2	Xp21.3	27468941..27749942
173	DCX	Xq23	111293779..111412192
174	DDX3X	Xp11.4	41333308..41364472
175	DDX53	Xp22.11	22999960..23003589
176	DGAT2L6	Xq13.1	70177483..70205704
177	DGKK	Xp11.22	50365409..50470825
178	DHRSX	Xp22.33 and Yp11.2	X:2219506..2500976;Y:2219506..2500976
179	DIAPH2	Xq21.33	96684842..97604997
180	DIPK2B	Xp11.3	45148373..45200876
181	DKC1	Xq28	154762864..154777689
182	DLG3	Xq13.1	70444835..70505490
183	DMD	Xp21.2-p21.1	31119222..33339388
184	DMRTC1	Xq13.1	72872025..72943837
185	DMRTC1B	Xq13.1	72776890..72848803
186	DNAAF6	Xq22.3	107206611..107244247
187	DNASE1L1	Xq28	154401236..154412101
188	DOCK11	Xq24	118495815..118686147
189	DRP2	Xq22.1	101219786..101264502
190	DUSP21	Xp11.3	44844021..44844888
191	DUSP9	Xq28	153642441..153651326
192	DYNLT3	Xp11.4	37838836..37847571
193	EBP	Xp11.23	48521808..48528716
194	EDA	Xq13.1	69616113..70039472
195	EDA2R	Xq12	66595637..66639269
196	EFHC2	Xp11.3	44147872..44343672
197	EFNB1	Xq13.1	68829021..68842160
198	EGFL6	Xp22.2	13569601..13633575
199	EIF1AX	Xp22.12	20124525..20141838
200	EIF2S3	Xp22.11	24054956..24078810
201	ELF4	Xq26.1	130063955..130111927
202	ELK1	Xp11.23	47635520..47650604
203	EMD	Xq28	154379295..154381523

204	ENOX2	Xq26.1	130622325..130903209
205	EOLA1	Xq28	149540601..149555345
206	EOLA2	Xq28	149929532..149938491
207	ERAS	Xp11.23	48826513..48829869
208	ERCC6L	Xq13.1	72204665..72239027
209	ESX1	Xq22.2	104250038..104254933
210	ETDA	Xq26.3	135251861..135253584
211	ETDB	Xq26.3	135118955..135120526
212	ETDC	Xq26.3	135309480..135309659
213	EZHIP	Xp11.22	51406948..51408843
214	F8	Xq28	154835792..155022723
215	F8A1	Xq28	154886355..154888061
216	F8A2	Xq28	155382095..155383801
217	F8A3	Xq28	155456914..155458620
218	F9	Xq27.1	139530739..139563459
219	FAAH2	Xp11.21	57121591..57489196
220	FAM104B	Xp11.21	55143102..5516118
221	FAM120C	Xp11.21	54068324..54183254
222	FAM133A	Xq21.32	93673681..93712265
223	FAM156A	Xp11.21	52947254..52995472
224	FAM156B	Xp11.21	52891592..52908560
225	FAM199X	Xq22.2	104157066..104195902
226	FAM236A	Xq13.1	72938206..72938958
227	FAM236B	Xq13.1	72781865..72782660
228	FAM236C	Xq13.1	72912615..72913401
229	FAM236D	Xq13.1	72807425..72808210
230	FAM3A	Xq28	154506171..154516232
231	FAM47A	Xp21.1	34129752..34132314
232	FAM47B	Xp21.1	34942796..34944915
233	FAM47C	Xp21.1	37008366..37011664
234	FAM50A	Xq28	154444141..154450654
235	FAM9A	Xp22.31	8790795..8801383
236	FAM9B	Xp22.31	9024232..9034127
237	FAM9C	Xp22.2	13035617..13044620
238	FANCB	Xp22.2	14689524..14873069
239	FATE1	Xq28	151716035..151723194
240	FGD1	Xp11.22	54445454..54496234
241	FGF13	Xq26.3-q27.1	138614727..139205023
242	FGF16	Xq21.1	77447389..77457278
243	FHL1	Xq26.3	136146702..136211359
244	FLNA	Xq28	154348531..154374634

245	FMR1	Xq27.3	147911919..147951125
246	FMR1NB	Xq27.3-q28	147981337..148026665
247	FOXO4	Xq13.1	71095851..71103532
248	FOXP3	Xp11.23	49250438..49264710
249	FOXR2	Xp11.21	55623400..55626192
250	FRMD7	Xq26.2	132076990..132128020
251	FRMPD3	Xq22.3	107449652..107605251
252	FRMPD4	Xp22.2	11822439..12724523
253	FTHL17	Xp21.2	31071233..31072041
254	FTSJ1	Xp11.23	48476021..48486364
255	FUNDC1	Xp11.3	44523639..44542859
256	FUNDC2	Xq28	155026844..155060304
257	G6PD	Xq28	154531390..154547569
258	GAB3	Xq28	154675249..154751566
259	GABRA3	Xq28	152166234..152451315
260	GABRE	Xq28	151953124..151974676
261	GABRQ	Xq28	152637895..152658965
262	GAGE1	Xp11.23	49599013..49608538
263	GAGE10	Xp11.23	49303646..49319844
264	GAGE12C	Xp11.23	49532177..49539538
265	GAGE12D	Xp11.23	49541733..49549094
266	GAGE12E	Xp11.23	49551289..49558645
267	GAGE12F	Xp11.23	49560842..49568205
268	GAGE12G	Xp11.23	49570397..49577757
269	GAGE12H	Xp11.23	49579949..49587301
270	GAGE12J	Xp11.23	49322037..49329384
271	GAGE13	Xp11.23	49331582..49338949
272	GAGE2A	Xp11.23	49589496..49596824
273	GATA1	Xp11.23	48786590..48794311
274	GCNA	Xq13.1	71578437..71613583
275	GDI1	Xq28	154437154..154443467
276	GDPD2	Xq13.1	70423314..70433381
277	GEMIN8	Xp22.2	13984185..14029892
278	GJB1	Xq13.1	71215239..71225516
279	GK	Xp21.2	30653423..30731462
280	GLA	Xq22.1	101397803..101407925
281	GLOD5	Xp11.23	48761747..48773648
282	GLRA2	Xp22.2	14448779..14731812
283	GLUD2	Xq24	121047610..121050094
284	GNL3L	Xp11.22	54530219..54645854
285	GPC3	Xq26.2	133535745..133985594

286	GPC4	Xq26.2	133300103..133415489
287	GPKOW	Xp11.23	49113407..49123735
288	GPM6B	Xp22.2	13770939..13938638
289	GPR101	Xq26.3	137023929..137033995
290	GPR119	Xq26.1	130379449..130385674
291	GPR143	Xp22.2	9725346..9778602
292	GPR173	Xp11.22	53048789..53080615
293	GPR174	Xq21.1	79144688..79175318
294	GPR34	Xp11.4	41688973..41697275
295	GPR50	Xq28	151176584..151182856
296	GPR82	Xp11.4	41724181..41730130
297	GPRASP1	Xq22.1	102651483..102659083
298	GRIA3	Xq25	123184278..123490915
299	GRIPAP1	Xp11.23	48973723..49002264
300	GRPR	Xp22.2	16123565..16153518
301	GSPT2	Xp11.22	51743442..51746232
302	GTPBP6	X;Y	X:304759..318796;Y:304759..318796
303	GUCY2F	Xq22.3-q23	109372906..109482086
304	GYG2	Xp22.33	2828930..2882818
305	H2AB1	Xq28	154884972..154885558
306	H2AB2	Xq28	155380709..155381299
307	H2AB3	Xq28	155459415..155460005
308	H2AP	Xp11.4	37990779..37991314
309	H2BW1	Xq22.2	104011147..104013708
310	H2BW2	Xq22.2	104039956..104042454
311	HAUS7	Xq28	153447668..153495465
312	HCCS	Xp22.2	11111332..11123086
313	HCFC1	Xq28	153947557..153971818
314	HDAC6	Xp11.23	48801398..48824982
315	HDAC8	Xq13.1	72329516..72572843
316	HDX	Xq21.1	84317878..84502453
317	HEPH	Xq12	66162671..66268863
318	HMGB3	Xq28	150980507..150990771
319	HMGN5	Xq21.1	81113699..81201913
320	HNRNPH2	Xq22.1	101408222..101414133
321	HPRT1	Xq26.2-q26.3	134460165..134500668
322	HS6ST2	Xq26.2	132626015..132961370
323	HSD17B10	Xp11.22	53431258..53434376
324	HSFX1	Xq28	149774068..149776867
325	HSFX2	Xq28	149592512..149595314
326	HSFX3	Xq28	149548210..149549924

327	HSFX4	Xq28	149929573..149931287
328	HTATSF1	Xq26.3	136497229..136512346
329	HTR2C	Xq23	114584086..114910061
330	HUWE1	Xp11.22	53532096..53686719
331	IDH3G	Xq28	153785768..153794375
332	IDS	Xq28	149476988..149505306
333	IGBP1	Xq13.1	70133447..70166324
334	IGSF1	Xq26.1	131273506..131289464
335	IKBKG	Xq28	154541238..154565046
336	IL13RA1	Xq24	118727606..118805228
337	IL13RA2	Xq23	115003982..115017616
338	IL1RAPL1	Xp21.3-p21.2	28587446..29956718
339	IL1RAPL2	Xq22.3	104566199..105767829
340	IL2RG	Xq13.1	71107404..71111577
341	IL3RA	X;Y	X:1336785..1382689;Y:1336785..1382689
342	IL9R	Xq28 and Yq12	X:155997696..156013020;Y:57184072..57197
343	INTS6L	Xq26.3	135520660..135582510
344	IQSEC2	Xp11.22	53225813..53321350
345	IRAK1	Xq28	154010507..154019902
346	IRS4	Xq22.3	108719946..108736563
347	ITGB1BP2	Xq13.1	71301750..71305371
348	ITIH6	Xp11.22	54748918..54798255
349	ITM2A	Xq21.1	79360384..79367334
350	JADE3	Xp11.3	46912301..47061242
351	KCND1	Xp11.23	48961380..48971844
352	KCNE5	Xq23	109623700..109625172
353	KDM5C	Xp11.22	53176277..53225207
354	KDM6A	Xp11.3	44873188..45112779
355	KIAA1210	Xq24	119078635..119151130
356	KIF4A	Xq13.1	70290104..70420886
357	KLF8	Xp11.21	55908123..56291531
358	KLHL13	Xq24	117897813..118131050
359	KLHL15	Xp22.11	23983720..24027186
360	KLHL34	Xp22.12	21654690..21658330
361	KLHL4	Xq21.31	87517802..87670050
362	KRBOX4	Xp11.3	46447297..46474639
363	L1CAM	Xq28	153861514..153886173
364	LAGE3	Xq28	154477775..154479281
365	LAMP2	Xq24	120426148..120469349
366	LANCL3	Xp21.1	37571661..37684463
367	LAS1L	Xq12	65512582..65534787

368	LDOC1	Xq27.1	141173235..141177129
369	LHFPL1	Xq23	112630648..112679938
370	LOC101059915	Xq13.1	71667546..71671527
371	LONRF3	Xq24	118974614..119018355
372	LPAR4	Xq21.1	78747720..78758714
373	LRCH2	Xq23	115110616..115234096
374	LUZP4	Xq23	115289715..115307563
375	MAGEA1	Xq28	153179284..153183880
376	MAGEA10	Xq28	152133310..152138577
377	MAGEA11	Xq28	149688193..149717268
378	MAGEA12	Xq28	152733757..152737669
379	MAGEA2	Xq28	152749863..152753884
380	MAGEA2B	Xq28	152714586..152718607
381	MAGEA3	Xq28	152698794..152702347
382	MAGEA4	Xq28	151912474..151925170
383	MAGEA6	Xq28	152766136..152769689
384	MAGEA8	Xq28	149881183..149885825
385	MAGEA9	Xq28	149781877..149787737
386	MAGEA9B	Xq28	149581653..149587511
387	MAGEB1	Xp21.2	30243731..30252040
388	MAGEB10	Xp21.3	27807990..27823014
389	MAGEB16	Xp21.1	35798342..35803772
390	MAGEB17	Xp22.2	16167481..16171464
391	MAGEB18	Xp21.3	26138343..26140736
392	MAGEB2	Xp21.2	30215563..30220089
393	MAGEB3	Xp21.2	30230657..30237495
394	MAGEB4	Xp21.2	30242000..30244187
395	MAGEB5	Xp21.3	26216169..26218270
396	MAGEB6	Xp21.3	26192440..26195646
397	MAGEC1	Xq27.2	141903894..141909374
398	MAGEC2	Xq27.2	142202342..142205290
399	MAGEC3	Xq27.2	141838316..141897832
400	MAGED1	Xp11.22	51803076..51902354
401	MAGED2	Xp11.21	54807745..54816015
402	MAGED4	Xp11.22	52184888..52192268
403	MAGED4B	Xp11.22	52061827..52069272
404	MAGEE1	Xq13.3	76427710..76431342
405	MAGEE2	Xq13.3	75782987..75785254
406	MAGEH1	Xp11.21	55452127..55453566
407	MAGIX	Xp11.23	49162665..49168774
408	MAGT1	Xq21.1	77825747..77895568

409	MAMLD1	Xq28	150361572..150514173
410	MAOA	Xp11.3	43655006..43746817
411	MAOB	Xp11.3	43766610..43882450
412	MAP3K15	Xp22.12	19360059..19515508
413	MAP7D2	Xp22.12	20006713..20116907
414	MAP7D3	Xq26.3	136213220..136256482
415	MBNL3	Xq26.2	132369320..132490035
416	MBTPS2	Xp22.12	21839617..21885423
417	MCF2	Xq27.1	139581770..139708167
418	MCTS1	Xq24	120604101..120621159
419	MECP2	Xq28	154021573..154097717
420	MED12	Xq13.1	71118596..71142450
421	MED14	Xp11.4	40648305..40736159
422	MID1	Xp22.2	10445310..10833683
423	MID1IP1	Xp11.4	38801459..38806532
424	MID2	Xq22.3	107825735..107931637
425	MMGT1	Xq26.3	135960588..135973988
426	MORC4	Xq22.3	106940738..107000212
427	MORF4L2	Xq22.2	103675498..103688047
428	MOSPD1	Xq26.3	134887632..134915257
429	MOSPD2	Xp22.2	14873421..14922327
430	MPC1L	Xp11.4	40623428..40624136
431	MPP1	Xq28	154778684..154805485
432	MSL3	Xp22.2	11758159..11775772
433	MSN	Xq12	65588377..65741931
434	MTCP1	Xq28	155064034..155071136
435	MTM1	Xq28	150562653..150673143
436	MTMR1	Xq28	150692962..150765108
437	MTMR8	Xq11.2	64268081..64395452
438	MXRA5	Xp22.33	3308565..3346652
439	NAA10	Xq28	153929225..153935037
440	NALF2	Xq13.1	69504326..69532508
441	NAP1L2	Xq13.2	73212299..73214851
442	NAP1L3	Xq21.32	93670930..93673578
443	NBDY	Xp11.21	56729243..56819179
444	NCBP2L	Xq22.3	107777733..107795829
445	NDP	Xp11.3	43948776..43973390
446	NDUFA1	Xq24	119871832..119876662
447	NDUFB11	Xp11.3	47142216..47145491
448	NEXMIF	Xq13.3	74732856..74925452
449	NHS	Xp22.2-p22.13	17375200..17735994

450	NHSL2	Xq13.1	71910845..72153286
451	NKAP	Xq24	119920672..119943751
452	NLGN3	Xq13.1	71144841..71175307
453	NLGN4X	Xp22.32-p22.31	5890042..6228867
454	NLRP2B	Xp11.21	57677067..57680260
455	NONO	Xq13.1	71283635..71301168
456	NOX1	Xq22.1	100843324..100874359
457	NR0B1	Xp21.2	30304206..30309390
458	NRK	Xq22.3	105821786..105958610
459	NSDHL	Xq28	152831063..152869729
460	NUDT10	Xp11.22	51332231..51337525
461	NUDT11	Xp11.22	51490011..51496592
462	NUP62CL	Xq22.3	107123427..107206433
463	NXF2	Xq22.1	102247167..102326722
464	NXF2B	Xq22.1	102360395..102440008
465	NXF3	Xq22.1	103075810..103093143
466	NXT2	Xq23	109535824..109544698
467	NYX	Xp11.4	41447343..41475652
468	OCRL	Xq26.1	129540259..129592556
469	OFD1	Xp22.2	13714505..13773738
470	OGT	Xq13.1	71533104..71575892
471	OPHN1	Xq12	68042344..68433841
472	OPN1LW	Xq28	154144243..154159032
473	OPN1MW	Xq28	154182596..154196861
474	OPN1MW2	Xq28	154219756..154233286
475	OPN1MW3	Xq28	154257582..154271090
476	OR13H1	Xq26.2	131544074..131545000
477	OTC	Xp11.4	38352604..38421446
478	OTUD5	Xp11.23	48922024..48958381
479	OTUD6A	Xq13.1	70062457..70064179
480	P2RY10	Xq21.1	78945391..78963727
481	P2RY4	Xq13.1	70258166..70260204
482	P2RY8	X;Y	X:1462581..1537185;Y:1462581..1537185
483	PABIR2	Xq26.3	134769566..134797205
484	PABIR3	Xq26.3	134796364..134864771
485	PABPC1L2A	Xq13.2	73077276..73079512
486	PABPC1L2B	Xq13.2	73002939..73006106
487	PABPC5	Xq21.31	91434839..91438584
488	PAGE1	Xp11.23	49687447..49695984
489	PAGE2	Xp11.21	55089018..55092842
490	PAGE2B	Xp11.21	55028117..55078909

491	PAGE3	Xp11.21	55258415..55264916
492	PAGE4	Xp11.23	49829260..49834264
493	PAGE5	Xp11.21	55220346..55224108
494	PAK3	Xq23	110944397..111227361
495	PASD1	Xq28	151563675..151676739
496	PBDC1	Xq13.3	76173062..76178314
497	PCDH11X	Xq21.31	91779375..92623230
498	PCDH19	Xq22.1	100291644..100410273
499	PCSK1N	Xp11.23	48831096..48835610
500	PCYT1B	Xp22.11	24558087..24672887
501	PDHA1	Xp22.12	19343927..19361718
502	PDK3	Xp22.11	24465286..24550466
503	PDZD11	Xq13.1	70286595..70289927
504	PDZD4	Xq28	153802166..153830544
505	PFKFB1	Xp11.21	54932961..54998789
506	PGAM4	Xq21.1	77967949..77969638
507	PGK1	Xq21.1	78104248..78129295
508	PGRMC1	Xq24	119236285..119244466
509	PHEX	Xp22.11	22032325..22251310
510	PHF6	Xq26.2	134373312..134428790
511	PHF8	Xp11.22	53936680..54048936
512	PHKA1	Xq13.1	72578814..72714306
513	PHKA2	Xp22.13	18892298..18984114
514	PIGA	Xp22.2	15319451..15335554
515	PIM2	Xp11.23	48913182..48919024
516	PIN4	Xq13.1	72181676..72263964
517	PIR	Xp22.2	15384799..15493333
518	PJA1	Xq13.1	69160746..69165453
519	PLAC1	Xq26.3	134565838..134764322
520	PLCXD1	X;Y	X:276356..303356;Y:276356..303356
521	PLP1	Xq22.2	103776506..103792619
522	PLP2	Xp11.23	49171898..49175235
523	PLS3	Xq23	115561174..115650861
524	PLXNA3	Xq28	154458281..154477779
525	PLXNB3	Xq28	153764249..153779341
526	PNCK	Xq28	153669733..153687771
527	PNMA3	Xq28	153056470..153060462
528	PNMA5	Xq28	152988824..152994116
529	PNMA6A	Xq28	153072454..153075019
530	PNMA6E	Xq28	153395639..153414004
531	PNMA6F	Xq28	153317680..153321767

532	PNPLA4	Xp22.31	7898247..7927724
533	POF1B	Xq21.1	85277396..85379665
534	POLA1	Xp22.11-p21.3	24693918..24996986
535	PORCN	Xp11.23	48508992..48520814
536	POU3F4	Xq21.1	83508290..83512127
537	PPEF1	Xp22.13	18675067..18827917
538	PPP1R2C	Xp11.3	42777366..42778163
539	PPP1R3F	Xp11.23	49269793..49301469
540	PPP2R3B	X;Y	X:333933..386907;Y:333933..386907
541	PPP4R3C	Xp21.3	27460207..27463341
542	PQBP1	Xp11.23	48897930..48903143
543	PRAF2	Xp11.23	49071161..49074002
544	PRDX4	Xp22.11	23667493..23686397
545	PRICKLE3	Xp11.23	49174802..49186373
546	PRKX	Xp22.33	3604340..3713649
547	PRPS1	Xq22.3	107628510..107651026
548	PRPS2	Xp22.2	12791412..12824222
549	PRR32	Xq25	126819729..126821786
550	PRRG1	Xp21.1	37349364..37457291
551	PRRG3	Xq28	151694607..151705924
552	PSMD10	Xq22.3	108084207..108091542
553	PTCHD1	Xp22.11	23334396..23404374
554	PUDP	Xp22.31	6705838..7148153
555	PWWP3B	Xq22.3	106168305..106208956
556	RAB33A	Xq26.1	130110623..130184870
557	RAB39B	Xq28	155258235..155264491
558	RAB40A	Xq22.2	103493266..103519489
559	RAB40AL	Xq22.1	102937272..102938300
560	RAB41	Xq13.1	70282172..70285002
561	RAB9A	Xp22.2	13689128..13710504
562	RAB9B	Xp22.2	103776324..103832257
563	RADX	Xp22.3	106611978..106679439
564	RAI2	Xp22.13	17800049..17861298
565	RAP2C	Xq26.2	132203025..132219447
566	RBBP7	Xp22.2	16844341..16870362
567	RBM10	Xp11.3	47145221..47186813
568	RBM3	Xp11.23	48574484..48581162
569	RBM41	Xq22.3	107052102..107118822
570	RBMX	Xq26.3	136869192..136880725
571	RBMX2	Xq26.1	130401987..130413656
572	RBMXL3	Xq23	115189410..115192868

573	RENBP	Xq28	153935269..153944643
574	REPS2	Xp22.2	16946658..17196655
575	RGN	Xp11.3	47078443..47093313
576	RHOXF1	Xq24	120109051..120120438
577	RHOXF2	Xq24	120158613..120165630
578	RHOXF2B	Xq24	120072264..120077690
579	RIBC1	Xp11.22	53422873..53431115
580	RIPPLY1	Xq22.3	106900063..106903341
581	RLIM	Xq13.2	74582976..74614624
582	RNF113A	Xq24	119870475..119871733
583	RNF128	Xq22.3	106693838..106797016
584	RP2	Xp11.3	46837043..46882358
585	RPA4	Xq21.33	96883908..96885467
586	RPGR	Xp11.4	38269163..38327509
587	RPL10	Xq28	154398065..154402339
588	RPL36A	Xq22.1	101391011..101396155
589	RPL39	Xq24	119786504..119791630
590	RPS4X	Xq13.1	72272042..72277248
591	RPS6KA3	Xp22.12	20149911..20267097
592	RPS6KA6	Xq21.1	84058346..84188499
593	RRAGB	Xp11.21	55717749..55758774
594	RS1	Xp22.13	18639688..18672108
595	RTL3	Xq21.1	78656068..78659328
596	RTL4	Xq23	112083013..112457514
597	RTL5	Xq13.1	72127110..72131915
598	RTL8A	Xq26.3	135050932..135052135
599	RTL8B	Xq26.3	135020513..135022542
600	RTL8C	Xq26.3	135032355..135033546
601	RTL9	Xq23	110358848..110456334
602	S100G	Xp22.2	16650158..16654670
603	SAGE1	Xq26.3	135893716..135913062
604	SASH3	Xq26.1	129779949..129795201
605	SAT1	Xp22.11	23783173..23786210
606	SATL1	Xq21.1	85092284..85243779
607	SCML1	Xp22.13	17737128..17754985
608	SCML2	Xp22.13	18239313..18355118
609	SEPTIN6	Xq24	119615724..119693168
610	SERPINA7	Xq22.3	106032435..106038727
611	SH2D1A	Xq25	124346563..124373160
612	SH3BGRL	Xq21.1	81202102..81298547
613	SH3KBP1	Xp22.12	19533977..19887600

614	SHOX	X;Y	X:624344..659411;Y:624344..659411
615	SHROOM2	Xp22.2	9786429..9949443
616	SHROOM4	Xp11.22	50575534..50814194
617	SLC10A3	Xq28	154487311..154490629
618	SLC16A2	Xq13.2	74421493..74533916
619	SLC25A14	Xq26.1	130339919..130373357
620	SLC25A43	Xq24	119399336..119454478
621	SLC25A5	Xq24	119468444..119471396
622	SLC25A53	Xq22.2	104099214..104157009
623	SLC25A6	X;Y	X:1386152..1392113;Y:1386152..1392113
624	SLC35A2	Xp11.23	48903183..48911958
625	SLC38A5	Xp11.23	48458544..48470260
626	SLC6A14	Xq23	116436606..116461458
627	SLC6A8	Xq28	153687926..153696593
628	SLC7A3	Xq13.1	70925579..70931096
629	SLC9A6	Xq26.3	135973837..136047269
630	SLC9A7	Xp11.3	46599251..46759118
631	SLITRK2	Xq27.3	145817829..145829856
632	SLITRK4	Xq27.3	143622790..143636101
633	SMARCA1	Xq25-q26.1	129446506..129523490
634	SMC1A	Xp11.22	53374149..53422728
635	SMIM10	Xq26.3	134990991..134992473
636	SMIM10L2B	Xq26.3	135094985..135098712
637	SMIM9	Xq28	154823348..154834662
638	SMPX	Xp22.12	21705978..21758116
639	SMS	Xp22.12	21940709..21994837
640	SNX12	Xq13.1	71059247..71073426
641	SOWAHD	Xq24	119758588..119760202
642	SOX3	Xq27.1	140502985..140505069
643	SPACA5	Xp11.23	48004336..48009733
644	SPACA5B	Xp11.23	48130626..48132620
645	SPANXA1	Xq27.2	141583674..141584738
646	SPANXA2	Xq27.2	141589708..141590762
647	SPANXB1	Xq27.1	141002594..141003706
648	SPANXC	Xq27.2	141241463..141242517
649	SPANXD	Xq27.2	141697411..141698739
650	SPANXN1	Xq27.3	145247503..145256208
651	SPANXN2	Xq27.3	143711955..143720752
652	SPANXN3	Xq27.3	143508735..143517475
653	SPANXN4	Xq27.3	143025928..143038637
654	SPANXN5	Xp11.22	52796144..52797427

655	SPIN2A	Xp11.21	57134265..57147180
656	SPIN2B	Xp11.21	57119682..57121548
657	SPIN3	Xp11.21	56975120..56995541
658	SPIN4	Xq11.1	63347228..63351332
659	SPRY3	Xq28 and Yq12	X:155612586..155782459;Y:56954316..56968
660	SRPK3	Xq28	153781041..153785730
661	SRPX	Xp11.4	38149339..38220871
662	SRPX2	Xq22.1	100644199..100675788
663	SSR4	Xq28	153794159..153798512
664	SSX1	Xp11.23	48255392..48267444
665	SSX2	Xp11.22	52696896..52707227
666	SSX2B	Xp11.22	52751252..52761536
667	SSX3	Xp11.23	48346427..48356703
668	SSX4	Xp11.23	48383539..48393347
669	SSX4B	Xp11.23	48402082..48411960
670	SSX5	Xp11.23	48186220..48196795
671	SSX7	Xp11.22	52644061..52654900
672	STAG2	Xq25	123960560..124102656
673	STARD8	Xq13.1	68647666..68725836
674	STEEP1	Xq24	119538149..119565409
675	STK26	Xq26.2	132023302..132075943
676	STS	Xp22.31	7147290..7354641
677	SUV39H1	Xp11.23	48695554..48709016
678	SYAP1	Xp22.2	16719612..16765340
679	SYN1	Xp11.3-p11.23	47571901..47619857
680	SYP	Xp11.23	49187815..49200193
681	SYTL4	Xq22.1	100674491..100732121
682	SYTL5	Xp11.4	37888915..38128820
683	TAB3	Xp21.2	30827442..30889254
684	TAF1	Xq13.1	71366357..71530525
685	TAF7L	Xq22.1	101268257..101293083
686	TAF9B	Xq21.1	78129748..78139650
687	TAFAZZIN	Xq28	154411539..154421726
688	TASL	Xp21.2	30558809..30577766
689	TBC1D25	Xp11.23	48539714..48562609
690	TBC1D8B	Xq22.3	106802673..106876150
691	TBL1X	Xp22.31-p22.2	9463295..9719740
692	TBX22	Xq21.1	80014753..80031774
693	TCEAL1	Xq22.2	103628716..103630953
694	TCEAL2	Xq22.1	102125679..102127712
695	TCEAL3	Xq22.2	103607963..103609927

696	TCEAL4	Xq22.2	103576231..103587729
697	TCEAL5	Xq22.1	103273691..103276750
698	TCEAL6	Xq22.1	102138502..102142481
699	TCEAL7	Xq22.2	103330239..103332326
700	TCEAL8	Xq22.1	103252995..103255155
701	TCEAL9	Xq22.2	103356506..103358462
702	TCEANC	Xq22.2	13653141..13665409
703	TCP11X2	Xq22.1	102460315..102471812
704	TENM1	Xq25	124375903..125204312
705	TENT5D	Xq21.1	80335504..80445309
706	TEX11	Xq13.1	70511227..70908711
707	TEX13A	Xq22.3	105218929..105220694
708	TEX13B	Xq22.3	107980864..107982370
709	TEX13C	Xq25	125319871..125325214
710	TEX13D	Xq25	124332660..124336863
711	TEX28	Xq28	154271265..154295211
712	TFDP3	Xq26.2	133216662..133218354
713	TFE3	Xp11.23	49028726..49043357
714	TGIF2LX	Xq21.31	89921908..89922883
715	THOC2	Xq25	123600569..123733052
716	TIMM17B	Xp11.23	48893447..48898143
717	TIMM8A	Xq22.1	101345661..101348742
718	TIMP1	Xp11.3	47582436..47586789
719	TKTL1	Xq28	154295795..154330350
720	TLR7	Xp22.2	12867072..12890361
721	TLR8	Xp22.2	12906620..12923169
722	TMEM164	Xq23	110002369..110184251
723	TMEM185A	Xq28	149596556..149631792
724	TMEM187	Xq28	153972754..153983194
725	TMEM255A	Xq24	120251433..120311461
726	TMEM31	Xq22.2	103710909..103714032
727	TMEM35A	Xq22.1	101078879..101096367
728	TMEM47	Xp21.1	34627075..34657285
729	TMLHE	Xq28	155489011..155612952
730	TMSB15A	Xq22.1	102513682..102516739
731	TMSB15B	Xq22.2	103919155..103974426
732	TMSB4X	Xq22.2	12975110..12977223
733	TNMD	Xq22.1	100584936..100599885
734	TRAPPC2	Xq22.2	13712245..13734620
735	TREX2	Xq28	153444473..153446056
736	TRMT2B	Xq22.1	100973366..101052111

737	TRO	Xp11.21	54920824..54931431
738	TRPC5	Xq23	111768011..112082776
739	TRPC5OS	Xq23	111876051..111903990
740	TSC22D3	Xq22.3	107713221..107777086
741	TSPAN6	Xq22.1	100627108..100637104
742	TSPAN7	Xp11.4	38561542..38688918
743	TSPYL2	Xp11.22	53082367..53088540
744	TSR2	Xp11.22	54440404..54448032
745	TXLNG	Xp22.2	16786466..16844519
746	UBA1	Xp11.3	47190847..47215128
747	UBE2A	Xq24	119574563..119584423
748	UBL4A	Xq28	154483717..154486615
749	UBQLN2	Xp11.21	56563627..56567868
750	UPF3B	Xq24	119805311..119852963
751	UPRT	Xq13.3	75156369..75304897
752	USP11	Xp11.3	47233009..47248328
753	USP26	Xq26.2	133023168..133097109
754	USP27X	Xp11.23	49879484..49882558
755	USP51	Xp11.21	55484616..55489848
756	USP9X	Xp11.4	41085445..41236579
757	UTP14A	Xq26.1	129906164..129929752
758	UXT	Xp11.23	47651796..47659180
759	VAMP7	Xq28 and Yq12	X:155881345..155943769;Y:57067865..57130
760	VBP1	Xq28	155197007..155239841
761	VCX	Xp22.31	7842262..7844143
762	VCX2	Xp22.31	8169944..8171267
763	VCX3A	Xp22.31	6533618..6535118
764	VCX3B	Xp22.31	8464830..8466510
765	VEGFD	Xp22.2	15345596..15384413
766	VGLL1	Xq26.3	136532215..136556799
767	VMA21	Xq28	151396595..151409364
768	VSIG1	Xq22.3	108018879..108079184
769	VSIG4	Xq12	66021738..66040080
770	WAS	Xp11.23	48676636..48691427
771	WDR13	Xp11.23	48590033..48608869
772	WDR44	Xq24	118346073..118449961
773	WDR45	Xp11.23	49074442..49101178
774	WNK3	Xp11.22	54192823..54358900
775	WWC3	Xp22.2	10015254..10144474
776	XAGE1A	Xp11.22	52495808..52500812
777	XAGE1B	Xp11.22	52512077..52517068

778	XAGE2	Xp11.22	52369021..52375680
779	XAGE3	Xp11.22	52862528..52868083
780	XAGE5	Xp11.22	52811287..52818301
781	XG	Xp22.33	2752040..2816500
782	XIAP	Xq25	123859708..123913972
783	XK	Xp21.1	37685791..37732130
784	XKRX	Xq22.1	100886916..100959343
785	XPNPEP2	Xq26.1	129738979..129769536
786	YIPF6	Xq12-q13.1	68499051..68537282
787	YY2	Xp22.12	21855987..21858740
788	ZBED1	X;Y	X:2486435..2500976;Y:2486435..2500976
789	ZBTB33	Xq24	120250812..120258398
790	ZC3H12B	Xq11.2-q12	65034826..65507887
791	ZC4H2	Xq11.2	64915807..65034741
792	ZCCHC12	Xq24	118823824..118826968
793	ZCCHC13	Xq13.2	74304180..74305034
794	ZCCHC18	Xq22.2	104112523..104115842
795	ZDHHC15	Xq13.3	75368427..75523037
796	ZDHHC9	Xq26.1	129803288..129843886
797	ZFP92	Xq28	153411472..153426481
798	ZFX	Xp22.11	24148982..24216255
799	ZIC3	Xq26.3	137566127..137577691
800	ZMAT1	Xq22.1	101882290..101932027
801	ZMYM3	Xq13.1	71239624..71255290
802	ZNF157	Xp11.3	47370578..47414498
803	ZNF182	Xp11.23	47974851..48003989
804	ZNF185	Xq28	152898067..152973481
805	ZNF275	Xq28	153334153..153352926
806	ZNF280C	Xq26.1	130202707..130268899
807	ZNF41	Xp11.3	47445178..47483222
808	ZNF449	Xq26.3	135344796..135363413
809	ZNF630	Xp11.23	48057516..48071640
810	ZNF674	Xp11.3	46497725..46545421
811	ZNF711	Xq21.1	85243991..85273357
812	ZNF75D	Xq26.3	135248589..135344109
813	ZNF81	Xp11.23	47836902..47925627
814	ZRSR2	Xp22.2	15790484..15823260
815	ZXDA	Xp11.21	57905430..57910458
816	ZXDB	Xp11.21	57592011..57597477

Table S4. X-linked protein coding genes significantly changed in ALDH- cells upon XIST KD

Gene name	entrez	logfc	adjpv
ASB9	140462	10	0.000188482
GPM6B	2824	2.67832	0.000188482
HEPH	9843	2.58564	0.000188482
MAOA	4128	2.54855	0.000188482
NAP1L3	4675	2.47383	0.000188482
MCF2	4168	2.35641	0.000188482
ITM2A	9452	2.32728	0.000356325
APLN	8862	2.01795	0.000188482
POF1B	79983	1.94264	0.000188482
AR	367	1.81086	0.000188482
TSC22D3	1831	1.7164	0.000188482
F8	2157	1.71347	0.000188482
CTPS2	56474	1.51241	0.000188482
TCEAL1	9338	1.4857	0.000188482
MID1	4281	1.46283	0.000188482
PRKX	5613	1.37528	0.000188482
PIR	8544	1.35498	0.000188482
UPRT	139596	1.34233	0.000188482
MAGED1	9500	1.31675	0.000188482
CCNB3	85417	1.29623	0.000188482
SPIN2B	474343	1.25569	0.000356325
AFF2	2334	1.23972	0.000188482
CD99L2	83692	1.16931	0.000188482
ABCD1	215	1.1686	0.000188482
DOCK11	139818	1.16549	0.000188482
MAGEH1	28986	1.12439	0.000188482
RPL10	6134	1.11387	0.000188482
TBC1D8B	54885	1.10823	0.000188482
SLC25A6	293	1.02679	0.000188482
CUL4B	8450	1.02647	0.000188482
RPS4X	6191	1.0033	0.000188482
CHST7	56548	1.00079	0.000188482
NUDT11	55190	0.992049	0.000188482
PLXNA3	55558	0.96293	0.000188482
MAGEA11	4110	0.92749	0.000356325
G6PD	2539	0.886058	0.000188482
SYTL4	94121	0.873008	0.000356325
SRPX	8406	0.836579	0.000188482
NUDT10	170685	0.830389	0.00299777

SH3BGRL	6451	0.814369	0.000188482
MPP1	4354	0.774254	0.000188482
TSPAN6	7105	0.751968	0.000672504
TRAPPC2	6399	0.738444	0.000188482
RBMX	27316	0.728726	0.000188482
PRRG1	5638	0.715346	0.000188482
TSPYL2	64061	0.700279	0.000188482
EIF2S3	1968	0.694268	0.000188482
TCEAL8	90843	0.688691	0.000188482
KLHL13	90293	0.656905	0.000968067
ZNF75D	7626	0.642127	0.0122681
MAGED2	10916	0.619002	0.000188482
PPP2R3B	28227	0.613822	0.000516129
ERCC6L	54821	0.613332	0.0395137
FUNDC1	139341	0.610684	0.00616815
CENPI	2491	0.608364	0.00273805
TMEM185A	84548	0.601614	0.000822387
MAGEA12	4111	-0.616195	0.000188482
XIAP	331	-0.655525	0.000188482
ZXDB	158586	-0.661447	0.000188482
IDS	3423	-0.67062	0.00125696
FUNDC2	65991	-0.682915	0.000188482
SLC25A14	9016	-0.689113	0.00153436
LAS1L	81887	-0.695895	0.000188482
TIMP1	7076	-0.700166	0.000356325
GPKOW	27238	-0.706084	0.000188482
SSR4	6748	-0.720203	0.000188482
CSTF2	1478	-0.721257	0.000188482
DDX3X	1654	-0.744591	0.000188482
YIPF6	286451	-0.750141	0.000188482
ENOX2	10495	-0.760196	0.000188482
PQBP1	10084	-0.775602	0.000188482
HAUS7	55559	-0.793266	0.000188482
ZRSR2	8233	-0.835011	0.000188482
ALG13	79868	-0.836438	0.000188482
CHIC1	53344	-0.849514	0.000188482
PBDC1	51260	-0.854037	0.000188482
MAGT1	84061	-0.864965	0.000188482
APEX2	27301	-0.867123	0.000188482
DKC1	1736	-0.871972	0.000188482
SLC35A2	7355	-0.89384	0.000188482

MMGT1	93380	-0.927019	0.000188482
PJA1	64219	-0.928042	0.000188482
EIF1AX	1964	-0.95435	0.000188482
RLIM	51132	-0.981418	0.000188482
AKAP17A	8227	-1.01045	0.000188482
MAGEA6	4105	-1.01489	0.000188482
GNL3L	54552	-1.04424	0.000188482
RPGR	6103	-1.08697	0.000188482
TFE3	7030	-1.1515	0.000188482
FTSJ1	24140	-1.15211	0.000188482
MAGEA3	4102	-1.15925	0.000188482
IRAK1	3654	-1.16378	0.000188482
TIMM8A	1678	-1.1658	0.000188482
TXLNG	55787	-1.20973	0.000188482
AMMECR1	9949	-1.37305	0.000188482
SH3KBP1	30011	-1.43916	0.000188482
VMA21	203547	-1.47254	0.000188482
RAB39B	116442	-1.51823	0.000188482
CA5B	11238	-1.67752	0.000188482
PGRMC1	10857	-1.70382	0.000188482
SAT1	6303	-2.35889	0.000188482

Table S5. X-linked protein coding genes significantly changed in ALDH+ cells upon XIST KD

Gene name	entrez	logfc	adjpv
ITM2A	9452	3.23395	0.000157
NAP1L3	4675	2.36606	0.000157
AR	367	2.22013	0.000157
AMOT	154796	2.20124	0.000157
HEPH	9843	2.15512	0.000157
GPM6B	2824	2.08925	0.000157
MAOA	4128	2.06063	0.000157
AFF2	2334	1.62891	0.000157
F8	2157	1.57067	0.000157
TSC22D3	1831	1.54094	0.000157
SH3BGRL	6451	1.28568	0.000157
TSPAN7	7102	1.28474	0.001429
PIR	8544	1.24661	0.000157
CCNB3	85417	1.2363	0.000157
MID1	4281	1.23003	0.000157
DOCK11	139818	1.20124	0.000157
ZNF280C	55609	1.10574	0.000157
RBMX	27316	1.08533	0.000157
CUL4B	8450	1.05127	0.000157
TCEAL1	9338	1.03743	0.000157
PRKX	5613	1.01058	0.000157
CD99L2	83692	0.995797	0.000157
CTPS2	56474	0.993131	0.000157
KLF8	11279	0.929498	0.000157
ABCD1	215	0.910092	0.000157
BRWD3	254065	0.843848	0.000157
TBC1D8B	54885	0.843531	0.005051
NUDT11	55190	0.807065	0.000157
SPIN2B	474343	0.791167	0.001667
RPL10	6134	0.770022	0.000157
GABRE	2564	0.764964	0.000157
EIF2S3	1968	0.762917	0.000157
MAGEH1	28986	0.760304	0.000157
TCEAL8	90843	0.722956	0.000157
TRAPPC2	6399	0.715146	0.000157
TAB3	257397	0.710211	0.000157
SLC25A6	293	0.683989	0.000157
FMR1	2332	0.679566	0.000157
RPS4X	6191	0.673412	0.000157

KLHL13	90293	0.671687	0.000157
UPRT	139596	0.648741	0.000301
TAF9B	51616	0.643417	0.000157
SPIN4	139886	0.636596	0.000822
PRPS2	5634	0.622768	0.000157
SRPX	8406	0.609896	0.000157
AMMECR1	9949	-0.62797	0.000157
MCTS1	28985	-0.66387	0.000157
CSTF2	1478	-0.69256	0.000157
MAGT1	84061	-0.69477	0.000157
NDUFA1	4694	-0.73458	0.000157
DKC1	1736	-0.74086	0.000157
MAGEA3	4102	-0.74789	0.000157
TMSB4X	7114	-0.75818	0.000157
RLIM	51132	-0.76102	0.000157
ZRSR2	8233	-0.77743	0.000157
ENOX2	10495	-0.78563	0.000157
CLCN5	1184	-0.78941	0.026434
VMA21	203547	-0.7908	0.000157
ALG13	79868	-0.81934	0.000157
SSR4	6748	-0.82091	0.000157
PQBP1	10084	-0.82327	0.000157
SLC25A14	9016	-0.83293	0.000157
APEX2	27301	-0.83589	0.000157
PBDC1	51260	-0.84829	0.000157
PJA1	64219	-0.85228	0.000157
FUNDC2	65991	-0.85634	0.000157
TIMP1	7076	-0.86282	0.000157
SLC35A2	7355	-0.8735	0.000157
TXLNG	55787	-0.89731	0.000157
GPKOW	27238	-0.92637	0.000157
ARFGEF2	10564	-0.95109	0.000157
MAGEA6	4105	-0.97236	0.000157
PHEX	5251	-0.99305	0.001549
FTSJ1	24140	-1.00648	0.000157
HNRNPH2	3188	-1.01032	0.02926
RPGR	6103	-1.02666	0.000157
HAUS7	55559	-1.02806	0.000157
AKAP17A	8227	-1.04737	0.000157
ARFGAP1	55738	-1.10449	0.000157
TFE3	7030	-1.13605	0.000157

IRAK1	3654	-1.21375	0.000157
RAB39B	116442	-1.2502	0.000157
CA5B	11238	-1.37381	0.000157
PGRMC1	10857	-1.55124	0.000157
SAT1	6303	-2.61113	0.000157

Table S6. 66 X-linked protein coding genes significantly changed in ALDH- & ALDH+ cells upon DOX-induced XIST KD

	ALDH- (DOX vs. CTL)		ALDH+ (DOX vs. CTL)	
Gene name	logfc	adjpv	logfc	adjpv
GPM6B	2.67832	0.000188482	2.08925	0.000156611
HEPH	2.58564	0.000188482	2.15512	0.000156611
MAOA	2.54855	0.000188482	2.06063	0.000156611
NAP1L3	2.47383	0.000188482	2.36606	0.000156611
ITM2A	2.32728	0.000356325	3.23395	0.000156611
AR	1.81086	0.000188482	2.22013	0.000156611
TSC22D3	1.7164	0.000188482	1.54094	0.000156611
F8	1.71347	0.000188482	1.57067	0.000156611
CTPS2	1.51241	0.000188482	0.993131	0.000156611
TCEAL1	1.4857	0.000188482	1.03743	0.000156611
MID1	1.46283	0.000188482	1.23003	0.000156611
PRKX	1.37528	0.000188482	1.01058	0.000156611
PIR	1.35498	0.000188482	1.24661	0.000156611
UPRT	1.34233	0.000188482	0.648741	0.000301055
CCNB3	1.29623	0.000188482	1.2363	0.000156611
SPIN2B	1.25569	0.000356325	0.791167	0.00166695
AFF2	1.23972	0.000188482	1.62891	0.000156611
CD99L2	1.16931	0.000188482	0.995797	0.000156611
ABCD1	1.1686	0.000188482	0.910092	0.000156611
DOCK11	1.16549	0.000188482	1.20124	0.000156611
MAGEH1	1.12439	0.000188482	0.760304	0.000156611
RPL10	1.11387	0.000188482	0.770022	0.000156611
TBC1D8B	1.10823	0.000188482	0.843531	0.00505089
SLC25A6	1.02679	0.000188482	0.683989	0.000156611
CUL4B	1.02647	0.000188482	1.05127	0.000156611
RPS4X	1.0033	0.000188482	0.673412	0.000156611
NUDT11	0.992049	0.000188482	0.807065	0.000156611
SRPX	0.836579	0.000188482	0.609896	0.000156611
SH3BGRL	0.814369	0.000188482	1.28568	0.000156611
TRAPPC2	0.738444	0.000188482	0.715146	0.000156611
RBMX	0.728726	0.000188482	1.08533	0.000156611
EIF2S3	0.694268	0.000188482	0.762917	0.000156611
TCEAL8	0.688691	0.000188482	0.722956	0.000156611
KLHL13	0.656905	0.000968067	0.671687	0.000156611
FUNDC2	-0.682915	0.000188482	-0.856335	0.000156611
SLC25A14	-0.689113	0.00153436	-0.832933	0.000156611
TIMP1	-0.700166	0.000356325	-0.862821	0.000156611

GPKOW	-0.706084	0.000188482	-0.926373	0.000156611
SSR4	-0.720203	0.000188482	-0.820905	0.000156611
CSTF2	-0.721257	0.000188482	-0.692556	0.000156611
ENOX2	-0.760196	0.000188482	-0.785627	0.000156611
PQBP1	-0.775602	0.000188482	-0.823268	0.000156611
HAUS7	-0.793266	0.000188482	-1.02806	0.000156611
ZRSR2	-0.835011	0.000188482	-0.77743	0.000156611
ALG13	-0.836438	0.000188482	-0.81934	0.000156611
PBDC1	-0.854037	0.000188482	-0.848294	0.000156611
MAGT1	-0.864965	0.000188482	-0.694768	0.000156611
APEX2	-0.867123	0.000188482	-0.835889	0.000156611
DKC1	-0.871972	0.000188482	-0.740858	0.000156611
SLC35A2	-0.89384	0.000188482	-0.8735	0.000156611
PJA1	-0.928042	0.000188482	-0.852282	0.000156611
RLIM	-0.981418	0.000188482	-0.761018	0.000156611
AKAP17A	-1.01045	0.000188482	-1.04737	0.000156611
MAGEA6	-1.01489	0.000188482	-0.972359	0.000156611
RPGR	-1.08697	0.000188482	-1.02666	0.000156611
TFE3	-1.1515	0.000188482	-1.13605	0.000156611
FTSJ1	-1.15211	0.000188482	-1.00648	0.000156611
MAGEA3	-1.15925	0.000188482	-0.747893	0.000156611
IRAK1	-1.16378	0.000188482	-1.21375	0.000156611
TXLNG	-1.20973	0.000188482	-0.897308	0.000156611
AMMECR1	-1.37305	0.000188482	-0.627972	0.000156611
VMA21	-1.47254	0.000188482	-0.790797	0.000156611
RAB39B	-1.51823	0.000188482	-1.2502	0.000156611
CA5B	-1.67752	0.000188482	-1.37381	0.000156611
PGRMC1	-1.70382	0.000188482	-1.55124	0.000156611
SAT1	-2.35889	0.000188482	-2.61113	0.000156611

Table S7. Upregulated miRNAs in ALDH- BCCs upon DOX-induced XIST KD

Transcript ID Array Design	Chromosome	Genomic Position	Strand	logFC
U78	chr1	173834760-173834824	-	3.534317
U31	chr11	62620797-62620867	-	3.463941
U56	chr20	2637270-2637340	+	3.424029
hsa-miR-193b-3p	chr16	14397874-14397895	+	3.157176
hsa-miR-324-5p	chr17	7126661-7126683	-	3.140992
U13	chr8	33370992-33371096	+	3.111984
hsa-miR-145-5p	chr5	148810224-148810246	+	3.08599
hsa-miR-3651	chr9	95054743-95054766	-	2.842974
U3-2B	chr17	18967233-18967449	-	2.766613
U3-2	chr17	18965225-18965441	+	2.766613
U3-3	chr17	19093342-19093558	-	2.766613
U3-4	chr17	19015733-19015949	-	2.766613
U31	chr11	62620797-62620867	-	2.644046
hsa-miR-4706	chr14	65511415-65511439	+	2.638897
ACA34	chr12	49048165-49048301	-	2.624172
hsa-miR-30d-5p	chr8	135817162-135817183	-	2.602393
hsa-miR-4445-3p	chr3	109321718-109321738	+	2.591362
hsa-miR-205-5p	chr1	209605511-209605532	+	2.571057
hsa-miR-500a-3p	chrX	49773090-49773111	+	2.485219
hsa-miR-1180-3p	chr17	19247826-19247847	-	2.365863
hsa-miR-1244	chr12	9392068-9392093	-	2.361126
hsa-miR-422a	chr15	64163188-64163209	-	2.34108
hsa-miR-6511b-5p	chr16	2156721-2156744	-	2.316526
hsa-miR-374b-5p	chrX	73438422-73438443	-	2.21853
ACA24	chr4	119200345-119200475	+	2.205028
ENSG00000206903	chr15	65577799-65577929	-	2.205028
ENSG00000207130	chr3	128433414-128433548	-	2.205028
U49A	chr17	16343350-16343420	+	2.169275
ENSG00000265941	chrHG79_PATCH	136225630-136225703	+	2.16202
U36B	chr9	136216949-136217019	+	2.16202
HBII-429	chr6	133137941-133138016	+	2.12631
hsa-miR-3613-3p	chr13	50570562-50570585	-	2.080468
hsa-miR-106b-3p	chr7	99691625-99691646	-	2.074827
mgh28S-2409	chr11	93466394-93466466	-	2.073599
ENSG00000201009	chr7	132437783-132437886	+	2.064015
U46	chr1	45242164-45242261	+	2.064015
hsa-miR-1301-3p	chr2	25551520-25551543	-	2.044556
U49B	chr17	16342823-16342870	+	2.028193
hsa-miR-224-3p	chrX	151127056-151127078	-	2.022298
ACA33	chr6	133138358-133138490	+	2.021606
U83A	chr22	39711218-39711312	-	1.991974
HBII-85-29	chr15	25351667-25351751	+	1.950581
hsa-miR-23b-5p	chr9	97847509-97847530	+	1.927768
hsa-miR-494-3p	chr14	101496018-101496039	+	1.922762
hsa-miR-130a-3p	chr11	57408725-57408746	+	1.921955
hsa-let-7a-2-3p	chr11	122017231-122017252	-	1.921139
hsa-miR-1184	chrX	154115651-154115673	-	1.920432
U56	chr20	2637270-2637340	+	1.88781
hsa-mir-6722	chr9	139641345-139641422	-	1.880618
hsa-miR-619-5p	chr12	109230738-109230759	-	1.867344

U35A	chr19	49994432-49994517	+	1.859163
hsa-miR-6069	chr22	35732761-35732781	-	1.852859
hsa-miR-24-2-5p	chr19	13947140-13947161	-	1.843696
U50B	chr6	86387307-86387377	-	1.822715
U50B	chr6	86387307-86387377	-	1.819104
U49A	chr17	16343350-16343420	+	1.818201
U49B	chr17	16342823-16342870	+	1.818201
SNORD126	chr14	20794609-20794685	-	1.808492
hsa-miR-1247-3p	chr14	102026663-102026686	-	1.806143
hsa-miR-1260a	chr14	77732574-77732591	+	1.794699
hsa-miR-6787-5p	chr17	80194549-80194570	+	1.787455
hsa-miR-195-5p	chr17	6920986-6921006	-	1.753019
U17a	chr1	28833877-28834083	+	1.733552
hsa-miR-660-5p	chrX	49777864-49777885	+	1.72877
U3	chr17	19091329-19091545	+	1.716718
hsa-miR-30a-3p	chr6	72113257-72113278	-	1.705035
hsa-miR-500a-5p	chrX	49773051-49773073	+	1.700147
U18A	chr15	66795583-66795652	-	1.699174
hsa-miR-335-5p	chr7	130135967-130135989	+	1.699065
ACA13	chr5	111497182-111497314	+	1.697716
U101	chr6	133136446-133136518	+	1.657502
hsa-mir-5095	chr1	53400602-53400689	+	1.65255
hsa-miR-105-5p	chrX	151560737-151560759	-	1.639763
hsa-miR-19b-3p	chr13	92003499-92003521	+	1.63682
U105	chr19	10218327-10218411	+	1.631656
hsa-miR-140-5p	chr16	69967006-69967027	+	1.614873
hsa-miR-3128	chr2	178120712-178120734	-	1.603887
ENSG00000263358	chrHSCHR6_MHC_SSTO	31494098-31494173	-	1.597295
ENSG00000263359	chrHSCHR6_MHC_COX	31491609-31491684	-	1.597295
ENSG00000263625	chrHSCHR6_MHC_MCF	31580504-31580579	-	1.597295
ENSG00000265116	chrHSCHR6_MHC_MANN	31543668-31543743	-	1.597295
ENSG00000265849	chrHSCHR6_MHC_APD	31515442-31515517	-	1.597295
ENSG00000266295	chrHSCHR6_MHC_QBL	31494434-31494509	-	1.597295
ENSG00000266419	chrHSCHR6_MHC_DBB	31486334-31486409	-	1.597295
U83	chr6	31504151-31504226	-	1.597295
ACA10	chr16	2012335-2012467	-	1.573891
ENSG00000207187	chr2	30410300-30410432	+	1.573891
ACA40	chr11	93468276-93468402	-	1.567552
U58C	chr18	47015614-47015678	-	1.567301
hsa-miR-486-3p	chr8	41517961-41517981	-	1.559817
U49A	chr17	16343350-16343420	+	1.558777
hsa-miR-652-3p	chrX	109298617-109298637	+	1.554919
hsa-miR-1973	chr4	117220906-117220924	+	1.550804
hsa-miR-6735-5p	chr1	43914215-43914239	+	1.550647
hsa-miR-6741-5p	chr1	226109816-226109837	-	1.548031
hsa-miR-362-5p	chrX	49773576-49773599	+	1.542963
hsa-miR-378a-5p	chr5	149112392-149112413	+	1.532536
snR39B	chr3	186502585-186502654	+	1.5203
hsa-miR-4787-3p	chr3	50712561-50712584	+	1.51761
U38A	chr1	45243514-45243584	+	1.463227
hsa-miR-1910-5p	chr16	85775275-85775295	-	1.450817
hsa-miR-877-5p	chr6	30552109-30552128	+	1.448689

hsa-miR-584-5p	chr5	148441936-148441957	-	1.443523
hsa-miR-181b-5p	chr1	198828054-198828076	-	1.441647
ENSG00000208308	chr2	135894198-135894325	+	1.440656
U18A	chr15	66795583-66795652	-	1.437489
hsa-miR-181d-5p	chr19	13985724-13985746	+	1.427786
hsa-miR-6879-5p	chr11	64785982-64786003	+	1.423406
hsa-miR-501-5p	chrX	49774343-49774364	+	1.415493
hsa-miR-4800-5p	chr4	2251854-2251874	-	1.404276
hsa-miR-125a-3p	chr19	52196559-52196580	+	1.401833
U25	chr11	62623037-62623103	-	1.396992
U59A	chr12	57038811-57038885	-	1.396312
HBII-316	chr2	29136528-29136616	+	1.385685
hsa-miR-4284	chr7	73125657-73125674	+	1.384381
hsa-miR-589-3p	chr7	5535465-5535488	-	1.372104
U44	chr1	173835104-173835166	-	1.371348
hsa-miR-193b-5p	chr16	14397837-14397858	+	1.348932
hsa-miR-874-3p	chr5	136983271-136983292	-	1.348181
ACA57	chr12	6690639-6690775	-	1.346774
hsa-miR-28-3p	chr3	188406622-188406643	+	1.342284
U58C	chr18	47015614-47015678	-	1.34195
ACA41	chr2	207026952-207027083	+	1.335778
U79	chr1	173834486-173834570	-	1.32753
hsa-miR-210-3p	chr11	568112-568133	-	1.324541
U29	chr11	62621376-62621440	-	1.320099
hsa-miR-328-3p	chr16	67236230-67236251	-	1.305605
hsa-miR-6836-5p	chr7	2297186-2297207	-	1.304896
hsa-miR-550a-3p	chr7	29720364-29720385	-	1.300545
hsa-miR-1246	chr2	177465752-177465770	-	1.295942
hsa-miR-4286	chr8	10524498-10524514	+	1.293604
hsa-miR-4299	chr11	11678208-11678225	-	1.290776
hsa-miR-3201	chr22	48670176-48670192	+	1.287406
hsa-miR-339-5p	chr7	1062626-1062648	-	1.286979
hsa-miR-6872-3p	chr3	50310706-50310726	+	1.284731
HBII-85-26	chr15	25344645-25344742	+	1.269637
hsa-miR-935	chr19	54485616-54485638	+	1.261583
U91	chr18	47340731-47340813	+	1.257837
mgU12-22-U4-8	chr18	47340393-47340813	+	1.257837
U55	chr1	45241537-45241610	+	1.247943
hsa-miR-6726-3p	chr1	1231490-1231509	-	1.246579
hsa-miR-5739	chr22	28855913-28855932	+	1.239068
U26	chr11	62622764-62622838	-	1.236404
hsa-miR-342-5p	chr14	100576010-100576030	+	1.235548
hsa-mir-7515	chr2	6790505-6790571	+	1.220845
hsa-miR-199a-5p	chr1	172113732-172113754	-	1.220807
hsa-miR-767-5p	chrX	151561953-151561975	-	1.215125
hsa-miR-34a-5p	chr1	9211794-9211815	-	1.207396
HBII-135	chr17	16344540-16344612	+	1.204935
ACA33	chr6	133138358-133138490	+	1.197077
hsa-miR-6075	chr5	1510882-1510902	-	1.19224
hsa-miR-6127	chr1	22959821-22959839	-	1.186211
hsa-miR-3148	chr8	29814834-29814855	-	1.185769
hsa-mir-4722	chr16	88782686-88782745	-	1.172131

hsa-miR-668-5p	chr14	101521601-101521619	+	1.171976
hsa-miR-769-5p	chr19	46522219-46522240	+	1.171336
hsa-miR-6796-3p	chr19	40875794-40875814	+	1.167355
ENSG00000266284	chrHG79_PATCH	136225991-136226063	+	1.166538
U36A	chr9	136217311-136217382	+	1.166538
HBII-85-15	chr15	25326433-25326526	+	1.161535
hsa-miR-4461	chr5	134263774-134263796	+	1.156336
hsa-miR-1303	chr5	154065387-154065408	+	1.143162
hsa-miR-4423-3p	chr1	85599525-85599545	+	1.140028
hsa-miR-331-3p	chr12	95702256-95702276	+	1.137342
hsa-miR-222-5p	chrX	45606479-45606500	-	1.136117
hsa-miR-6086	chrX	13608412-13608431	+	1.12869
hsa-miR-4444	chr2	178077496-178077513	+	1.120055
hsa-miR-1269a	chr4	67142608-67142629	+	1.114157
hsa-mir-6886	chr19	11224150-11224210	+	1.112268
hsa-miR-8089	chr5	180470413-180470436	-	1.106533
HBII-202	chr16	89627838-89627909	+	1.106412
hsa-miR-181c-5p	chr19	13985539-13985560	+	1.100485
hsa-miR-19a-3p	chr13	92003193-92003215	+	1.097915
hsa-miR-6809-3p	chr2	218765236-218765256	-	1.097766
ENSG00000265706	chr2	29150849-29150926	+	1.094172
hsa-miR-214-5p	chr1	172107997-172108018	-	1.093054
U105B	chr19	10220433-10220511	+	1.09238
hsa-miR-6757-5p	chr12	53450733-53450754	+	1.088374
ACA3	chr11	8705774-8705903	+	1.085319
hsa-miR-3687	chr21	9826237-9826260	+	1.08025
hsa-miR-3682-3p	chr2	54076273-54076293	-	1.079759
SNORA38B	chr17	65736785-65736915	+	1.078657
hsa-mir-4449	chr4	53578849-53578914	+	1.076773
hsa-mir-6511a-1	chr16	15019794-15019860	+	1.07612
hsa-mir-6511b-1	chr16	2156670-2156754	-	1.07612
hsa-mir-6511b-2	chr16	15227932-15228002	-	1.07612
hsa-mir-6511a-2	chr16	16418445-16418511	+	1.07612
hsa-mir-6511a-3	chr16	16462733-16462799	+	1.07612
hsa-mir-6511a-4	chr16	18437870-18437936	-	1.07612
ACA41	chr2	207026952-207027083	+	1.071135
hsa-mir-345	chr14	100774196-100774293	+	1.063304
HBII-289	chr2	101889398-101889511	-	1.063257
U32A	chr19	49993225-49993301	+	1.056433
hsa-miR-4467	chr7	102111919-102111940	+	1.0482
HBI-6	chr4	53579416-53579537	+	1.046553
hsa-miR-941	chr20	62550872-62550894	+	1.041934
hsa-mir-6891	chr6	31323001-31323093	-	1.03178
ENSG00000252277	chr15	25353415-25353499	+	1.029118
hsa-miR-6801-3p	chr19	52725327-52725346	+	1.027367
hsa-mir-500b	chrX	49775280-49775358	+	1.021134
hsa-miR-6085	chr15	62635310-62635328	+	1.016861
ENSG00000207118	chr11	122929617-122929703	-	1.009574
hsa-mir-486-2	chr8	41517962-41518025	+	1.008621
HBII-85-26	chr15	25344645-25344742	+	1.006266
hsa-miR-125b-2-3p	chr21	17962610-17962631	+	1.005839
hsa-mir-6165	chr17	47588179-47588262	+	0.999567

ACA8	chr11	93465527-93465665	-	0.987527
hsa-mir-4446	chr3	113313723-113313789	+	0.986598
hsa-mir-3653	chr22	29729147-29729256	-	0.983466
SNORD125	chr22	29729152-29729247	-	0.983466
hsa-mir-6872	chr3	50310667-50310728	+	0.980945
hsa-miR-596	chr8	1765412-1765432	+	0.978845
hsa-miR-6835-3p	chr6	34208491-34208513	+	0.977076
hsa-miR-4485	chr11	10529824-10529843	-	0.97704
hsa-miR-17-3p	chr13	92002909-92002930	+	0.976792
ACA11	chr4	1976363-1976487	+	0.972186
hsa-miR-199a-3p	chr1	172113694-172113715	-	0.971538
hsa-miR-199b-3p	chr9	131007024-131007045	-	0.971538
ACA57	chr12	6690639-6690775	-	0.947874
U47	chr1	173833508-173833572	-	0.941785
hsa-miR-1269b	chr17	12820630-12820651	-	0.941189
spike_in-control-36				0.941155
hsa-miR-7109-3p	chr22	32017459-32017480	-	0.940204
U14B	chr11	17096200-17096291	-	0.937038
U82	chr2	232325079-232325153	-	0.936967
hsa-miR-3194-5p	chr20	50069485-50069505	-	0.932764
hsa-miR-3935	chr16	56279489-56279510	+	0.930661
hsa-miR-4646-5p	chr6	31668847-31668868	-	0.930567
ENSG00000252349	chr17	19565313-19565403	+	0.930345
U17b	chr1	28835070-28835274	+	0.928806
hsa-mir-3128	chr2	178120673-178120738	-	0.928411
ENSG00000252921	chr18	23879079-23879219	-	0.92729
hsa-miR-8075	chr13	113917280-113917303	+	0.923771
U81	chr1	173833284-173833360	-	0.922608
hsa-miR-4269	chr2	240227167-240227187	+	0.921262
hsa-mir-4298	chr11	1880694-1880766	-	0.919725
ENSG00000252921	chr18	23879079-23879219	-	0.912615
U38B	chr1	45244062-45244130	+	0.911159
hsa-miR-3619-5p	chr22	46486939-46486960	+	0.911051
hsa-miR-6511a-3p	chr16	15019837-15019858	+	0.909405
hsa-miR-7975	chr19	55634593-55634610	-	0.909079
ENSG00000222345	chr3	52725394-52725469	+	0.908393
ENSG00000221252	chr6	42379938-42380134	-	0.907479
hsa-miR-3609	chr7	98479323-98479346	+	0.90541
U99	chr11	62432894-62433042	+	0.905352
hsa-miR-29a-3p	chr7	130561507-130561528	-	0.902537
hsa-miR-628-3p	chr15	55665152-55665172	-	0.898075
hsa-miR-15a-5p	chr13	50623303-50623324	-	0.89702
ENSG00000212615	chr14	45557446-45557511	+	0.89657
U71d	chr20	37062505-37062642	-	0.896524
hsa-miR-4529-3p	chr18	53146501-53146521	+	0.888225
hsa-mir-8075	chr13	113917234-113917313	+	0.887603
hsa-miR-4487	chr11	47422574-47422592	+	0.885144
hsa-miR-4649-5p	chr7	44150450-44150473	+	0.88351
ENSG00000239080	chrX	135216476-135216579	-	0.882993
hsa-mir-1229	chr5	179225278-179225346	-	0.880355
HBII-85-28	chr15	25349788-25349880	+	0.875147
U68	chr19	17973397-17973529	+	0.874289

hsa-miR-196b-5p	chr7	27209147-27209168	-	0.869513
ACA61	chr1	28906276-28906405	-	0.864135
hsa-miR-210-5p	chr11	568150-568171	-	0.863128
ENSG00000202252	chr11	122930043-122930130	-	0.861139
hsa-miR-6126	chr16	3535422-3535439	-	0.860094
U50	chr6	86387012-86387086	-	0.852656
hsa-miR-7845-5p	chr2	208031126-208031146	+	0.848493
ENSG00000252277	chr15	25353415-25353499	+	0.848451
ENSG00000238430	chr1	173629236-173629339	-	0.845229
hsa-miR-3187-3p	chr19	813627-813646	+	0.844234
hsa-miR-320a	chr8	22102488-22102509	-	0.842089
ENSG00000212397	chr11	112473077-112473175	-	0.83951
hsa-let-7e-3p	chr19	52196091-52196112	+	0.838957
hsa-miR-212-3p	chr17	1953584-1953604	-	0.838707
hsa-miR-30e-3p	chr1	41220085-41220106	+	0.83543
hsa-mir-4725	chr17	29902288-29902377	+	0.833918
ENSG00000251838	chr11	37723675-37723770	-	0.828828
hsa-mir-6836	chr7	2297150-2297212	-	0.828661
hsa-miR-128-3p	chr2	136423016-136423036	+	0.826084
HBII-251	chr1	31441010-31441084	-	0.825013
hsa-miR-34c-3p	chr11	111384209-111384230	+	0.82481
hsa-miR-4306	chr13	100295377-100295393	+	0.815618
hsa-miR-181a-2-3p	chr9	127454797-127454818	+	0.810896
hsa-mir-3652	chr12	104324203-104324333	+	0.807875
U18C	chr15	66793590-66793656	-	0.805438
hsa-miR-661	chr8	145019374-145019397	-	0.8049
hsa-mir-4423	chr1	85599477-85599556	+	0.793895
hsa-miR-665	chr14	101341412-101341431	+	0.792856
hsa-miR-6779-3p	chr17	37071270-37071290	+	0.788933
hsa-miR-27b-3p	chr9	97847787-97847807	+	0.788759
hsa-miR-1231	chr1	201777743-201777762	+	0.786253
hsa-miR-486-5p	chr8	41518002-41518023	-	0.784538
HBII-99	chr20	47897220-47897309	+	0.784349
SNORA38B	chr17	65736785-65736915	+	0.783994
U77	chr1	173835439-173835508	-	0.782786
HBII-239	chr16	71792305-71792390	-	0.780267
hsa-miR-4750-3p	chr19	50391466-50391487	+	0.779992
hsa-miR-6751-5p	chr11	64897423-64897445	-	0.7792
hsa-mir-1260a	chr14	77732561-77732633	+	0.779067
hsa-mir-6804	chr19	55742253-55742320	-	0.777198
ENSG00000252709	chr11	66762950-66763096	-	0.771681
U34	chr19	49994164-49994229	+	0.771451
U75	chr1	173836017-173836076	-	0.766441
hsa-miR-3124-5p	chr1	249120582-249120602	+	0.766294
ACA43	chr9	139620556-139620691	-	0.765314
hsa-miR-208a-5p	chr14	23857846-23857867	-	0.765054
hsa-miR-6859-3p	chr1	17369-17391	-	0.764657
hsa-mir-6780a	chr17	40860102-40860169	-	0.7644
U45B	chr1	76255162-76255232	+	0.762061
U71a	chr20	37055949-37056086	-	0.753266
hsa-miR-330-3p	chr19	46142267-46142289	-	0.752811
U69	chrX	118921316-118921447	-	0.750585

ENSG00000239128	chr3	47292013-47292116	-	0.749758
hsa-miR-5579-3p	chr11	79133213-79133234	-	0.747391
hsa-mir-8084	chr8	94041979-94042067	+	0.746998
hsa-miR-21-5p	chr17	57918634-57918655	+	0.746406
hsa-mir-1263	chr3	163889259-163889344	-	0.744804
ENSG00000263362	chrHSCHR6_MHC_COX	31792223-31792289	+	0.741207
ENSG00000265156	chrHSCHR6_MHC_QBL	31795112-31795178	+	0.741207
ENSG00000265707	chrHSCHR6_MHC_DBB	31787042-31787108	+	0.741207
ENSG00000266300	chrHSCHR6_MHC_APD	31816211-31816277	+	0.741207
U52	chr6	31804853-31804916	+	0.741207
hsa-miR-570-5p	chr3	195426296-195426317	+	0.732512
hsa-miR-548ai	chr6	99572500-99572521	+	0.732512
hsa-miR-4786-5p	chr2	240882480-240882501	-	0.732167
hsa-miR-6787-3p	chr17	80194583-80194604	+	0.731978
SNORD123	chr5	9548948-9549017	+	0.731297
hsa-miR-16-2-3p	chr3	160122585-160122606	+	0.7302
hsa-miR-8071	chr14	106087488-106087507	+	0.727903
U61	chrX	135961358-135961430	-	0.72784
hsa-miR-6774-5p	chr16	85951958-85951982	+	0.727582
hsa-miR-7113-3p	chr11	67800367-67800389	+	0.727501
hsa-miR-3615	chr17	72744802-72744822	+	0.725845
ACA52	chr11	811681-811814	+	0.725229
hsa-miR-365a-3p	chr16	14403197-14403218	+	0.724007
hsa-miR-365b-3p	chr17	29902497-29902518	+	0.724007
snR38C	chr17	74554872-74554951	+	0.723332
gi:555853				0.72035
hsa-miR-126-3p	chr9	139565105-139565126	+	0.720053
U88	chr2	234197322-234197587	+	0.716064
hsa-miR-193a-5p	chr17	29887035-29887056	+	0.716053
hsa-mir-500b	chrX	49775280-49775358	+	0.715578
hsa-miR-660-3p	chrX	49777900-49777920	+	0.714914
ACA6	chr3	39449882-39450030	+	0.712445
HBII-142	chr3	184043484-184043559	+	0.710236
U17a	chr1	28833877-28834083	+	0.709982
hsa-mir-4656	chr7	4828196-4828270	-	0.708733
hsa-miR-224-5p	chrX	151127103-151127123	-	0.708344
ENSG00000238368	chr2	120674894-120675000	+	0.706841
hsa-miR-1181	chr19	10514181-10514201	-	0.706617
hsa-mir-6869	chr20	1373544-1373605	-	0.706113
hsa-miR-30c-2-3p	chr6	72086667-72086688	-	0.705089
hsa-miR-3180	chr16	2186077-2186095	-	0.704412
hsa-mir-648	chr22	18463634-18463727	-	0.704329
hsa-miR-4454	chr4	164014759-164014778	-	0.703983
U75	chr1	173836017-173836076	-	0.703538
ACA44	chr1	28906893-28907024	-	0.701055
ENSG00000252840	chr1	151500307-151500414	-	0.701055
hsa-miR-4767	chrX	7065910-7065932	+	0.69588
ENSG00000253090	chr6	108306863-108306983	-	0.695851
ENSG00000238934	chr1	161110998-161111126	-	0.695734
U103B	chr1	31421962-31422052	-	0.695497
U103	chr1	31408533-31408623	-	0.695497
hsa-mir-7851	chr12	42717502-42717661	-	0.695061

hsa-mir-1288	chr17	16185328-16185402	+	0.694924
hsa-mir-10b	chr2	177015031-177015140	+	0.69407
U104	chr17	62223438-62223517	+	0.694047
ENSG00000222489	chr14	20791338-20791485	-	0.69389
ACA62	chr17	62223699-62223831	+	0.693649
ENSG00000253013	chr4	187051175-187051309	+	0.693181
U77	chr1	173835439-173835508	-	0.689099
hsa-miR-33b-3p	chr17	17717171-17717192	-	0.688713
U100	chr15	66639544-66639680	-	0.685428
hsa-mir-7846	chr1	12227000-12227095	+	0.68527
hsa-miR-5004-5p	chr6	33406128-33406149	+	0.684494
hsa-mir-517a	chr19	54215522-54215608	+	0.683606
hsa-mir-1254-2	chr10	23682334-23682396	+	0.6819
hsa-miR-6776-5p	chr17	2596190-2596208	-	0.681487
hsa-miR-18b-5p	chrX	133304114-133304136	-	0.680903
HBII-55	chr20	2634858-2634932	+	0.679246
hsa-miR-615-5p	chr12	54427751-54427772	+	0.678676
hsa-miR-29b-2-5p	chr1	207975837-207975858	-	0.678179
HBII-142	chr3	184043484-184043559	+	0.677994
hsa-mir-7151	chr10	69163109-69163168	-	0.675885
ACA67B	chr2	10586840-10586975	-	0.674579
SNORD124	chr17	38183795-38183898	-	0.670642
ACA15	chr7	56128163-56128295	+	0.669689
ENSG00000206785	chr7	65225039-65225173	+	0.669689
ENSG00000207062	chr7	64530916-64531050	+	0.669689
hsa-miR-204-5p	chr9	73424947-73424968	-	0.668264
hsa-miR-365a-5p	chr16	14403157-14403179	+	0.667096
hsa-miR-4313	chr15	76054627-76054646	-	0.665569
hsa-miR-3619-3p	chr22	46486970-46486991	+	0.661867
HBII-438A	chr15	25287121-25287187	+	0.658425
HBII-438B	chr15	25523490-25523556	+	0.658425
ENSG00000238581	chr21	39559551-39559656	-	0.658202
hsa-miR-3177-3p	chr16	1785039-1785059	+	0.656373
hsa-miR-5699-5p	chr10	687688-687709	-	0.654941
hsa-mir-3910-1	chr9	94398533-94398643	+	0.654771
hsa-miR-4730	chr17	78393227-78393249	+	0.654705
hsa-miR-3943	chr7	43190516-43190538	+	0.654286
mgU2-19-30	chr11	93454680-93455032	+	0.653405
ACA64	chr16	2015185-2015311	+	0.652996
hsa-miR-4738-3p	chr17	73780611-73780632	-	0.650649
hsa-mir-4662b	chr8	125834220-125834300	-	0.64764
ACA43	chr9	139620556-139620691	-	0.644872
ENSG00000202389	chr17	26349356-26349490	-	0.644516
hsa-miR-625-3p	chr14	65937871-65937892	+	0.6438
U59B	chr12	57037464-57037538	-	0.642542
hsa-mir-3917	chr1	26232853-26232945	-	0.642264
hsa-miR-5002-3p	chr3	123851835-123851855	+	0.638407
hsa-miR-4653-5p	chr7	100802763-100802784	+	0.638363
hsa-miR-6804-3p	chr19	55742253-55742274	-	0.637745
U15A	chr11	75111435-75111582	+	0.636322
ENSG00000239080	chrX	135216476-135216579	-	0.635776
hsa-miR-6824-5p	chr3	48671105-48671126	-	0.634299

hsa-miR-548ae	chr2	185243744-185243764	+	0.633216
hsa-mir-551a	chr1	3477259-3477354	-	0.630182
hsa-miR-937-5p	chr8	144895180-144895199	-	0.630091
ACA27	chr13	27829538-27829663	+	0.629759
hsa-mir-6819	chr22	36682893-36682953	-	0.628276
U15B	chr11	75115465-75115610	+	0.627342
hsa-mir-30a	chr6	72113254-72113324	-	0.627135
U60	chr16	2205024-2205106	-	0.626662
hsa-miR-548b-3p	chr6	119390227-119390248	-	0.625333
U38B	chr1	45244062-45244130	+	0.624508
hsa-miR-139-5p	chr11	72326146-72326168	-	0.623009
ENSG00000252238	chr2	4874591-4874674	-	0.621739
hsa-mir-922	chr3	197401367-197401447	-	0.62126
14qII-16	chr14	101439932-101440000	+	0.621075
ENSG00000206849	chr15	32220506-32220637	+	0.620099
hsa-miR-520h	chr19	54245820-54245841	+	0.616469
hsa-miR-3942-5p	chr15	35664522-35664543	-	0.616325
U66	chr1	93306276-93306408	+	0.615788
hsa-mir-890	chrX	145075793-145075869	-	0.615614
hsa-miR-1285-3p	chr2	70480065-70480086	-	0.615604
hsa-miR-550a-5p	chr7	30329431-30329453	+	0.612751
ENSG00000267818	chrHG344_PATCH	7076500-7076769	-	0.610893
U89	chr12	7076500-7076769	-	0.610893
mgh18S-121	chr17	27049600-27049671	+	0.610798
hsa-miR-518a-5p	chr19	54234273-54234292	+	0.610665
hsa-miR-527	chr19	54257285-54257304	+	0.610665
hsa-miR-4668-5p	chr9	114694380-114694402	+	0.609787
hsa-miR-615-3p	chr12	54427794-54427815	+	0.60961
hsa-miR-6875-5p	chr7	100465663-100465683	+	0.608044
ENSG00000207410	chr17	65267585-65267723	+	0.607315
hsa-miR-483-3p	chr11	2155372-2155392	-	0.606349
hsa-mir-6729	chr1	12089215-12089279	+	0.605229
hsa-miR-6829-5p	chr3	195609242-195609261	-	0.60412
hsa-miR-3197	chr21	42539491-42539513	+	0.602308
hsa-mir-548j	chr22	26951178-26951289	-	0.601001
hsa-mir-517b	chr19	54224330-54224396	+	0.599336
hsa-mir-663b	chr2	133014539-133014653	-	0.597967
ENSG00000199470	chr7	12740383-12740514	-	0.597558
hsa-mir-663b	chr2	133014539-133014653	-	0.595062
hsa-miR-4793-5p	chr3	48681672-48681695	-	0.594522
hsa-mir-222	chrX	45606421-45606530	-	0.593307
hsa-miR-502-3p	chrX	49779257-49779278	+	0.592897
hsa-miR-6783-5p	chr17	43012023-43012044	-	0.592443
hsa-miR-150-3p	chr19	50004054-50004075	-	0.591935
hsa-miR-30b-5p	chr8	135812813-135812834	-	0.5912
hsa-mir-3910-1	chr9	94398533-94398643	+	0.589926
hsa-miR-1229-5p	chr5	179225323-179225346	-	0.587785
HBII-436	chr15	25227141-25227215	+	0.587119
hsa-mir-130b	chr22	22007593-22007674	+	0.586579
hsa-miR-3195	chr20	60639868-60639884	+	0.586217
hsa-miR-567	chr3	111831663-111831685	+	0.585947
hsa-miR-6746-5p	chr11	61645724-61645745	-	0.585816

hsa-mir-149	chr2	241395418-241395506	+	0.585652

Table S8. Upregulated miRNAs in ALDH+ CSCs upon DOX-induced XIST KD

Transcript ID Array Design	Chromosome	Genomic Position	Strand	logFC
hsa-miR-23b-5p	chr9	97847509-97847530	+	3.94047353
hsa-miR-34a-5p	chr1	9211794-9211815	-	2.83454966
hsa-miR-130a-3p	chr11	57408725-57408746	+	2.33006943
hsa-miR-660-5p	chrX	49777864-49777885	+	1.919895
hsa-miR-4529-3p	chr18	53146501-53146521	+	1.8884633
hsa-miR-1909-3p	chr19	1816168-1816189	-	1.8793515
hsa-miR-196b-5p	chr7	27209147-27209168	-	1.8443965
hsa-miR-378a-5p	chr5	149112392-149112413	+	1.82688204
hsa-miR-30c-2-3p	chr6	72086667-72086688	-	1.8134695
hsa-miR-3607-5p	chr5	85916325-85916346	+	1.75874145
hsa-miR-1973	chr4	117220906-117220924	+	1.75026485
hsa-miR-210-3p	chr11	568112-568133	-	1.66679
hsa-miR-1184	chrX	154115651-154115673	-	1.5908628
U49A	chr17	16343350-16343420	+	1.5693785
U49B	chr17	16342823-16342870	+	1.5693785
hsa-mir-6836	chr7	2297150-2297212	-	1.5690949
hsa-miR-21-5p	chr17	57918634-57918655	+	1.553869
hsa-miR-891a-5p	chrX	145109359-145109380	-	1.54969985
hsa-miR-602	chr9	140732886-140732908	+	1.5444785
hsa-miR-181c-5p	chr19	13985539-13985560	+	1.4990165
hsa-miR-30c-1-3p	chr1	41223011-41223032	+	1.49844838
hsa-miR-342-5p	chr14	100576010-100576030	+	1.4957547
hsa-miR-34c-5p	chr11	111384176-111384198	+	1.49003148
hsa-miR-214-5p	chr1	172107997-172108018	-	1.459387
hsa-miR-1303	chr5	154065387-154065408	+	1.4568095
hsa-miR-4706	chr14	65511415-65511439	+	1.436606
hsa-miR-3180	chr16	2186077-2186095	-	1.3758335
ACA17	chr9	139621199-139621331	-	1.36307812
hsa-miR-4428	chr1	237634464-237634485	+	1.3299835
hsa-miR-1244	chr12	9392068-9392093	-	1.324915
hsa-miR-486-5p	chr8	41518002-41518023	-	1.30246275
hsa-miR-181d-5p	chr19	13985724-13985746	+	1.294253
hsa-miR-6802-5p	chr19	55751320-55751339	-	1.285233
hsa-miR-505-5p	chrX	139006355-139006376	-	1.281245
U56	chr20	2637270-2637340	+	1.2624785
U13	chr8	33370992-33371096	+	1.2406515
hsa-mir-6886	chr19	11224150-11224210	+	1.20610545
hsa-miR-3689a-5p	chr9	137741380-137741401	-	1.20097535
hsa-miR-3689b-5p	chr9	137742052-137742073	-	1.20097535
hsa-miR-3689e	chr9	137742460-137742481	-	1.20097535
hsa-miR-1301-3p	chr2	25551520-25551543	-	1.1963095
hsa-miR-769-3p	chr19	46522258-46522280	+	1.1915849
hsa-miR-760	chr1	94312436-94312455	+	1.1763126
U56	chr20	2637270-2637340	+	1.1630665
U31	chr11	62620797-62620867	-	1.1612055
ENSG00000207118	chr11	122929617-122929703	-	1.145004
hsa-miR-4299	chr11	11678208-11678225	-	1.14135295
hsa-miR-6750-5p	chr11	64665881-64665904	-	1.1357825
hsa-miR-324-3p	chr17	7126627-7126646	-	1.110287
hsa-miR-1587	chrX	39696816-39696835	+	1.1043685

hsa-miR-1180-3p	chr17	19247826-19247847	-	1.095223
hsa-miR-204-5p	chr9	73424947-73424968	-	1.0931597
hsa-miR-194-5p	chr1	220291548-220291569	-	1.0929855
hsa-miR-6848-5p	chr8	145540951-145540973	-	1.0889995
hsa-miR-34c-3p	chr11	111384209-111384230	+	1.08578
hsa-miR-296-3p	chr20	57392681-57392702	-	1.0693835
hsa-miR-8084	chr8	94041989-94042011	+	1.0616715
hsa-miR-767-5p	chrX	151561953-151561975	-	1.0604025
U18A	chr15	66795583-66795652	-	1.05983975
hsa-miR-3147	chr7	57472736-57472759	+	1.0591235
hsa-mir-500b	chrX	49775280-49775358	+	1.0584588
U38B	chr1	45244062-45244130	+	1.0532971
U28	chr11	62622093-62622167	-	1.0521255
hsa-miR-6797-5p	chr19	42373702-42373726	+	1.0496549
hsa-miR-205-3p	chr1	209605548-209605568	+	1.04671098
hsa-miR-4730	chr17	78393227-78393249	+	1.04153285
hsa-miR-1538	chr16	69599711-69599733	-	1.0409855
hsa-miR-4695-3p	chr1	19209698-19209719	-	1.04046805
hsa-miR-584-5p	chr5	148441936-148441957	-	1.0397187
hsa-miR-378h	chr5	154209026-154209046	+	1.03414665
hsa-miR-148b-3p	chr12	54731062-54731083	+	1.0326865
hsa-miR-505-3p	chrX	139006319-139006340	-	1.02907425
U81	chr1	173833284-173833360	-	1.0227555
hsa-miR-3136-5p	chr3	69098155-69098177	-	1.0188406
hsa-miR-500b-3p	chrX	49775330-49775349	+	1.01554345
hsa-miR-2110	chr10	115933910-115933931	-	1.01533275
hsa-miR-4649-5p	chr7	44150450-44150473	+	1.009083
hsa-miR-199a-3p	chr1	172113694-172113715	-	1.006222
hsa-miR-199b-3p	chr9	131007024-131007045	-	1.006222
hsa-miR-6511b-5p	chr16	2156721-2156744	-	1.0032345
hsa-miR-1181	chr19	10514181-10514201	-	0.9934479
hsa-miR-5571-5p	chr22	23228471-23228491	+	0.98400725
hsa-miR-663b	chr2	133014543-133014564	-	0.9685859
hsa-let-7a-2-3p	chr11	122017231-122017252	-	0.9597455
hsa-miR-489-3p	chr7	93113259-93113280	-	0.94957285
U3	chr17	19091329-19091545	+	0.9481365
hsa-miR-8075	chr13	113917280-113917303	+	0.9405205
hsa-miR-140-5p	chr16	69967006-69967027	+	0.9374405
hsa-mir-4701	chr12	49165758-49165820	-	0.9265903
hsa-miR-6133	chr7	132975705-132975723	+	0.9171336
hsa-miR-4778-5p	chr2	66585429-66585450	-	0.91451068
hsa-miR-4492	chr11	118781474-118781490	+	0.91067
hsa-miR-210-5p	chr11	568150-568171	-	0.9097833
U35B	chr19	50000976-50001063	+	0.898345
hsa-miR-4417	chr1	5624142-5624159	+	0.89706845
hsa-miR-6796-3p	chr19	40875794-40875814	+	0.89593395
hsa-miR-3180-3p	chr16	15005138-15005159	+	0.892512
hsa-miR-126-3p	chr9	139565105-139565126	+	0.8901401
hsa-miR-1910-5p	chr16	85775275-85775295	-	0.889184
hsa-miR-105-5p	chrX	151560737-151560759	-	0.87655
hsa-miR-7109-5p	chr22	32017492-32017512	-	0.875079
hsa-miR-7-5p	chr15	89155087-89155109	+	0.8712048

hsa-miR-6752-3p	chr11	67257766-67257786	+	0.85755419
hsa-mir-3653	chr22	29729147-29729256	-	0.8547708
SNORD125	chr22	29729152-29729247	-	0.8547708
hsa-miR-619-5p	chr12	109230738-109230759	-	0.8546185
hsa-miR-6780a-5p	chr17	40860142-40860164	-	0.8530669
ACA24	chr4	119200345-119200475	+	0.851487
ENSG00000206903	chr15	65577799-65577929	-	0.851487
ENSG00000207130	chr3	128433414-128433548	-	0.851487
hsa-miR-23c	chrX	20035219-20035240	-	0.850194
HBII-99	chr20	47897220-47897309	+	0.849244
hsa-miR-3188	chr19	18392939-18392961	+	0.84861775
snR39B	chr3	186502585-186502654	+	0.8475965
hsa-miR-6870-5p	chr20	10630320-10630338	-	0.84733825
hsa-miR-6860	chr11	66813154-66813175	+	0.8471285
hsa-miR-6877-5p	chr9	135927388-135927409	+	0.8432337
hsa-mir-7111	chr6	35438285-35438356	+	0.83231794
hsa-miR-8089	chr5	180470413-180470436	-	0.822348
U30	chr11	62621135-62621204	-	0.82093
hsa-miR-3197	chr21	42539491-42539513	+	0.81629755
hsa-miR-15a-5p	chr13	50623303-50623324	-	0.8111415
hsa-miR-145-5p	chr5	148810224-148810246	+	0.8087257
hsa-miR-149-5p	chr2	241395432-241395454	+	0.805904
U18A	chr15	66795583-66795652	-	0.805305
hsa-miR-550a-3-5p	chr7	29720405-29720424	-	0.8038365
hsa-mir-6768	chr16	2513968-2514039	+	0.78518485
hsa-miR-1913	chr6	166922852-166922873	-	0.78346445
hsa-miR-1231	chr1	201777743-201777762	+	0.780849
hsa-miR-195-5p	chr17	6920986-6921006	-	0.78011555
hsa-miR-224-5p	chrX	151127103-151127123	-	0.7784205
hsa-let-7c-5p	chr21	17912158-17912179	+	0.7760135
hsa-miR-4472	chr12	116866099-116866116	-	0.77420165
hsa-miR-27b-3p	chr9	97847787-97847807	+	0.7738605
hsa-miR-183-3p	chr7	129414768-129414789	-	0.7708
hsa-miR-297	chr4	111781780-111781800	-	0.770062
hsa-miR-6808-5p	chr1	1275062-1275083	-	0.763074
hsa-miR-3613-5p	chr13	50570601-50570622	-	0.7621915
hsa-miR-1269a	chr4	67142608-67142629	+	0.7615525
hsa-miR-615-3p	chr12	54427794-54427815	+	0.7607715
hsa-miR-365a-5p	chr16	14403157-14403179	+	0.7607595
ACA54	chr11	2985001-2985123	-	0.760425
hsa-miR-3183	chr17	925769-925790	-	0.75803685
hsa-miR-6832-3p	chr6	31601616-31601635	+	0.75713085
hsa-mir-4716	chr15	49461267-49461350	-	0.7565686
hsa-miR-193b-3p	chr16	14397874-14397895	+	0.755738
hsa-miR-629-5p	chr15	70371766-70371786	-	0.7475055
SNORD126	chr14	20794609-20794685	-	0.746567
HBII-316	chr2	29136528-29136616	+	0.746419
hsa-mir-1976	chr1	26881033-26881084	+	0.7444849
hsa-miR-129-1-3p	chr7	127847973-127847994	+	0.74438778
hsa-miR-1234-3p	chr8	145625478-145625499	-	0.73773593
hsa-miR-26b-5p	chr2	219267380-219267400	+	0.73625285
hsa-miR-28-5p	chr3	188406582-188406603	+	0.7345915

hsa-mir-4654	chr1	162126897-162126972	+	0.7325557
hsa-miR-4750-5p	chr19	50391434-50391455	+	0.7322515
ACA44	chr1	28906893-28907024	-	0.729728
ENSG00000252840	chr1	151500307-151500414	-	0.729728
hsa-miR-4284	chr7	73125657-73125674	+	0.729715
hsa-miR-3619-5p	chr22	46486939-46486960	+	0.7286625
hsa-miR-205-5p	chr1	209605511-209605532	+	0.7241015
hsa-let-7a-1	chr9	96938239-96938318	+	0.722117
hsa-miR-6779-3p	chr17	37071270-37071290	+	0.71746355
hsa-miR-7111-3p	chr6	35438335-35438356	+	0.715976
hsa-miR-6732-5p	chr1	37945836-37945855	+	0.714099
hsa-mir-4800	chr4	2251804-2251883	-	0.71369591
hsa-miR-27b-5p	chr9	97847745-97847766	+	0.7130925
hsa-miR-6716-3p	chr11	118514766-118514787	+	0.71130145
hsa-miR-5001-3p	chr2	233415203-233415224	-	0.70883335
hsa-miR-6721-5p	chr6	32137861-32137883	-	0.70508225
hsa-miR-6816-5p	chr22	20102249-20102269	-	0.7049155
hsa-mir-500a	chrX	49773039-49773122	+	0.6957655
hsa-mir-6804	chr19	55742253-55742320	-	0.69506535
hsa-miR-4286	chr8	10524498-10524514	+	0.6947875
hsa-miR-7109-3p	chr22	32017459-32017480	-	0.69385915
U49B	chr17	16342823-16342870	+	0.6935085
hsa-miR-6820-5p	chr22	38363575-38363593	+	0.69126385
hsa-miR-642b-3p	chr19	46178199-46178220	-	0.69042945
ACA20	chr6	160201282-160201413	-	0.68642775
HBII-251	chr1	31441010-31441084	-	0.6833945
hsa-miR-501-3p	chrX	49774380-49774401	+	0.681279
hsa-miR-6879-3p	chr11	64786022-64786042	+	0.67978575
hsa-miR-4487	chr11	47422574-47422592	+	0.6786735
hsa-miR-6886-5p	chr19	11224155-11224175	+	0.67769165
hsa-miR-331-3p	chr12	95702256-95702276	+	0.6698195
hsa-miR-6752-5p	chr11	67257721-67257742	+	0.669432
hsa-miR-28-3p	chr3	188406622-188406643	+	0.6692545
hsa-miR-1275	chr6	33967795-33967811	-	0.668866
hsa-miR-874-3p	chr5	136983271-136983292	-	0.6653935
ENSG00000239045	chr7	151765985-151766090	-	0.6649249
ACA33	chr6	133138358-133138490	+	0.6630685
HBII-85-8	chr15	25315578-25315674	+	0.655369
ENSG00000201009	chr7	132437783-132437886	+	0.654543
U46	chr1	45242164-45242261	+	0.654543
hsa-miR-589-5p	chr7	5535504-5535525	-	0.6541896
hsa-miR-4785	chr2	161264330-161264350	-	0.6525702
hsa-mir-4638	chr5	180649566-180649633	-	0.65135065
hsa-mir-663a	chr20	26188822-26188914	-	0.64695355
hsa-miR-6758-5p	chr12	57906476-57906498	+	0.6449945
hsa-miR-6836-5p	chr7	2297186-2297207	-	0.6445365
hsa-mir-6722	chr9	139641345-139641422	-	0.6439325
ACA43	chr9	139620556-139620691	-	0.6406775
hsa-miR-151a-3p	chr8	141742686-141742706	-	0.638764
HBII-436	chr15	25227141-25227215	+	0.63832615
hsa-miR-422a	chr15	64163188-64163209	-	0.637463
hsa-mir-924	chr18	37202087-37202139	-	0.6359209

HBI-115	chr5	76376259-76376396	-	0.6354868
hsa-miR-5688	chr3	85434911-85434932	+	0.63460682
hsa-miR-574-3p	chr4	38869713-38869734	+	0.633656
hsa-mir-323a	chr14	101492069-101492154	+	0.63343894
hsa-miR-6747-5p	chr11	62334515-62334538	-	0.632921
hsa-miR-328-3p	chr16	67236230-67236251	-	0.6328009
ACA57	chr12	6690639-6690775	-	0.6287405
ENSG00000221164	chr10	74885838-74885965	-	0.62794954
hsa-miR-378g	chr1	95211436-95211455	-	0.627934
hsa-miR-193b-5p	chr16	14397837-14397858	+	0.626289
hsa-miR-7112-5p	chr8	145317619-145317640	-	0.6234783
U3-2B	chr17	18967233-18967449	-	0.6229345
U3-2	chr17	18965225-18965441	+	0.6229345
U3-3	chr17	19093342-19093558	-	0.6229345
U3-4	chr17	19015733-19015949	-	0.6229345
hsa-mir-99b	chr19	52195865-52195934	+	0.622093
hsa-miR-1294	chr5	153726713-153726734	+	0.621716
hsa-miR-374b-5p	chrX	73438422-73438443	-	0.6203115
hsa-mir-425	chr3	49057581-49057667	-	0.61924085
U42B	chr17	27047568-27047634	+	0.6174149
hsa-mir-1282	chr15	44085857-44085957	-	0.61634775
hsa-mir-4785	chr2	161264321-161264393	-	0.61408455
hsa-mir-7515	chr2	6790505-6790571	+	0.613875
hsa-miR-6833-3p	chr6	32147633-32147653	+	0.61366947
hsa-miR-6759-5p	chr12	58142439-58142460	-	0.6121942
hsa-miR-3654	chr7	132719620-132719638	-	0.60742362
hsa-miR-184	chr15	79502182-79502203	+	0.60599565
hsa-miR-6085	chr15	62635310-62635328	+	0.605084
hsa-miR-4326	chr20	61918170-61918189	+	0.60425235
hsa-mir-4442	chr3	25706364-25706430	-	0.60188425
ENSG00000239154	chr11	101929036-101929139	-	0.601569
SNORD123	chr5	9548948-9549017	+	0.600807
U59A	chr12	57038811-57038885	-	0.600671
hsa-miR-3126-3p	chr2	69330859-69330880	+	0.59987905
spike_in-control-21				0.5988542
hsa-miR-6780b-5p	chr6	43402285-43402307	+	0.5965144
hsa-miR-3196	chr20	61870140-61870157	+	0.595908
hsa-miR-1228-5p	chr12	57588287-57588307	+	0.594774
hsa-miR-1298-3p	chrX	113949721-113949741	+	0.5934894
hsa-miR-4687-5p	chr11	3877303-3877324	+	0.59333145
U83A	chr22	39711218-39711312	-	0.592009
hsa-miR-4474-3p	chr9	20502275-20502296	-	0.59174046
hsa-miR-1228-3p	chr12	57588338-57588357	+	0.5912948
hsa-miR-147a	chr9	123007263-123007282	-	0.59056383
hsa-miR-486-3p	chr8	41517961-41517981	-	0.589998
hsa-miR-199a-5p	chr1	172113732-172113754	-	0.5870565
U44	chr1	173835104-173835166	-	0.586858
hsa-mir-205	chr1	209605478-209605587	+	0.58591265

Table S9. Predicted miRNAs targeted by XIST

MicroRNA	Target score
hsa-miR-4789-3p	100
hsa-miR-3692-5p	100
hsa-miR-548aj-5p	100
hsa-miR-548g-5p	100
hsa-miR-548x-5p	100
hsa-miR-212-3p	100
hsa-miR-583	100
hsa-miR-132-3p	100
hsa-miR-3662	100
hsa-miR-92a-2-5p	100
hsa-miR-518a-5p	99.99
hsa-miR-527	99.99
hsa-miR-374b-5p	99.99
hsa-miR-4495	99.98
hsa-miR-3646	99.98
hsa-miR-466	99.96
hsa-miR-4729	99.95
hsa-miR-217	99.95
hsa-miR-616-5p	99.94
hsa-miR-373-5p	99.94
hsa-miR-371b-5p	99.93
hsa-miR-3154	99.91
hsa-miR-3202	99.88
hsa-miR-3163	99.83
hsa-miR-4802-5p	99.78
hsa-miR-551b-5p	99.69
hsa-miR-186-5p	99.69
hsa-miR-3065-3p	99.61
hsa-miR-130a-5p	99.59
hsa-miR-4735-3p	99.58
hsa-miR-497-5p	99.57
hsa-miR-888-5p	99.55
hsa-miR-4693-3p	99.54
hsa-miR-3713	99.53
hsa-miR-5589-3p	99.51
hsa-miR-509-5p	99.49
hsa-miR-3925-5p	99.49
hsa-miR-3179	99.48
hsa-miR-4672	99.45
hsa-miR-509-3-5p	99.39
hsa-miR-3619-5p	99.33
hsa-miR-335-3p	99.31
hsa-miR-424-5p	99.27
hsa-miR-3613-3p	99.21
hsa-miR-5692a	99.17

hsa-miR-507	99.09
hsa-miR-4311	99.05
hsa-miR-193a-5p	99
hsa-miR-664-3p	98.96
hsa-miR-1277-5p	98.77
hsa-miR-4640-5p	98.27
hsa-miR-4282	98.23
hsa-miR-548aw	98.22
hsa-miR-3200-3p	98.21
hsa-miR-4312	98.19
hsa-miR-4671-3p	98.05
hsa-miR-4422	98.04
hsa-miR-1245b-3p	98.01
hsa-miR-5680	98.01
hsa-miR-520d-3p	97.96
hsa-miR-3151	97.94
hsa-miR-520b	97.9
hsa-miR-4659b-3p	97.9
hsa-miR-372	97.86
hsa-miR-214-3p	97.85
hsa-miR-520c-3p	97.82
hsa-miR-4646-3p	97.8
hsa-miR-4659a-3p	97.72
hsa-miR-4428	97.53
hsa-miR-338-5p	97.44
hsa-miR-520a-3p	97.26
hsa-miR-4458	97.22
hsa-miR-520e	97.18
hsa-miR-1275	97.18
hsa-miR-4650-5p	97.15
hsa-miR-4481	97.06
hsa-miR-377-3p	97.03
hsa-miR-1972	96.86
hsa-miR-4747-5p	96.3
hsa-miR-4472	96.18
hsa-let-7d-5p	96.16
hsa-miR-196a-5p	96.12
hsa-miR-196b-5p	96.08
hsa-miR-4782-3p	96.04
hsa-miR-2355-3p	96
hsa-miR-4712-3p	95.81
hsa-miR-5011-3p	95.8
hsa-miR-5196-5p	95.8
hsa-miR-3919	95.78
hsa-miR-548l	95.66
hsa-miR-20a-3p	95.56
hsa-miR-5696	95.53

hsa-miR-4447	95.17
hsa-miR-548an	95.08
hsa-miR-370	95
hsa-miR-548u	94.82
hsa-miR-219-5p	94.61
hsa-miR-4736	94.54
hsa-miR-3658	94.43
hsa-miR-5681a	94.41
hsa-miR-4506	94.22
hsa-miR-557	94.11
hsa-miR-607	93.98
hsa-miR-545-3p	93.92
hsa-miR-4731-3p	93.92
hsa-miR-374a-5p	93.65
hsa-miR-3609	93.64
hsa-miR-4698	93.58
hsa-miR-3908	93.51
hsa-miR-2355-5p	93.46
hsa-miR-3120-3p	93.01
hsa-miR-454-3p	92.99
hsa-miR-548x-3p	92.95
hsa-miR-548aj-3p	92.9
hsa-miR-4652-5p	92.87
hsa-miR-410	92.84
hsa-miR-4763-3p	92.26
hsa-miR-513c-3p	92.22
hsa-miR-548m	92.09
hsa-miR-4761-5p	91.58
hsa-miR-513b	91.47
hsa-miR-513a-3p	91.4
hsa-miR-4503	91.36
hsa-miR-374c-5p	91.27
hsa-miR-520f	91.21
hsa-miR-200b-3p	91.15
hsa-miR-200c-3p	91.13
hsa-miR-519a-3p	91
hsa-miR-4666a-3p	90.85
hsa-miR-519b-3p	90.66
hsa-miR-429	90.61
hsa-miR-7-2-3p	90.57
hsa-miR-5003-3p	90.56
hsa-miR-363-3p	90.55
hsa-miR-3161	90.53
hsa-miR-524-5p	90.49
hsa-miR-578	90.27
hsa-miR-3133	90
hsa-miR-548ah-5p	89.98

hsa-miR-519c-3p	89.9
hsa-miR-4476	89.73
hsa-miR-655	89.64
hsa-miR-520d-5p	89.62
hsa-miR-141-5p	89.57
hsa-miR-367-3p	89.47
hsa-miR-3123	89.37
hsa-miR-7-1-3p	89.34
hsa-miR-4694-3p	89.18
hsa-let-7b-5p	88.74
hsa-miR-4772-5p	88.72
hsa-miR-940	88.3
hsa-miR-548am-3p	88.13
hsa-miR-155-5p	87.87
hsa-let-7i-5p	87.82
hsa-let-7f-5p	87.81
hsa-miR-1207-5p	87.78
hsa-miR-4745-5p	87.74
hsa-miR-5585-3p	87.43
hsa-let-7e-5p	87.31
hsa-let-7g-5p	86.97
hsa-miR-4738-3p	86.96
hsa-miR-500a-3p	86.96
hsa-let-7a-1-5p	86.93
hsa-miR-889	86.65
hsa-miR-3682-3p	86.65
hsa-miR-369-3p	86.64
hsa-miR-3941	86.63
hsa-miR-3148	86.47
hsa-miR-769-3p	86.45
hsa-miR-548ah-3p	86.44
hsa-miR-224-3p	86.37
hsa-miR-548aa	86.36
hsa-miR-548t-3p	86.36
hsa-miR-4483	86.24
hsa-miR-323a-3p	86.23
hsa-miR-519d	86.22
hsa-let-7c	86.22
hsa-miR-548ae	86.14
hsa-miR-106a-5p	86.02
hsa-miR-548aq-3p	85.95
hsa-miR-373-3p	85.88
hsa-miR-4517	85.68
hsa-miR-4500	85.6
hsa-miR-3678-3p	85.45
hsa-miR-4291	85.45
hsa-miR-17-5p	85.15

hsa-miR-606	85.13
hsa-miR-548e	85.01
hsa-miR-522-3p	84.76
hsa-miR-548f	84.76
hsa-miR-98	84.6
hsa-miR-4432	84.55
hsa-miR-2114-5p	84.54
hsa-miR-4726-3p	84.46
hsa-miR-93-5p	84.12
hsa-miR-761	83.74
hsa-miR-502-3p	83.69
hsa-miR-659-5p	83.31
hsa-miR-619	83.2
hsa-miR-548ap-3p	82.92
hsa-miR-20b-5p	82.89
hsa-miR-5197-3p	82.7
hsa-miR-3942-5p	82.66
hsa-miR-330-3p	82.32
hsa-miR-450b-5p	82.18
hsa-miR-4700-3p	81.98
hsa-miR-5089	81.94
hsa-miR-1237	81.86
hsa-miR-5590-3p	81.82
hsa-miR-5579-3p	81.73
hsa-miR-539-5p	81.63
hsa-miR-202-3p	81.58
hsa-miR-4662b	81.31
hsa-miR-3689b-3p	81.25
hsa-miR-3689c	81.25
hsa-miR-4434	81.12
hsa-miR-539-3p	81.05
hsa-miR-423-5p	81.04
hsa-miR-944	80.97
hsa-miR-32-3p	80.96
hsa-miR-342-3p	80.91
hsa-miR-4427	80.17
hsa-miR-181c-5p	80.13
hsa-miR-526b-5p	80.08
hsa-miR-3910	79.93
hsa-miR-603	79.82
hsa-miR-302a-3p	79.79
hsa-miR-23b-3p	79.71
hsa-miR-4638-3p	79.71
hsa-miR-4520b-3p	79.54
hsa-miR-302b-3p	79.54
hsa-miR-492	79.53
hsa-miR-1303	79.52

hsa-miR-501-3p	79.41
hsa-miR-4665-5p	79.38
hsa-miR-3124-3p	79.36
hsa-miR-380-3p	79.35
hsa-miR-4277	79.04
hsa-miR-376c	79.02
hsa-miR-656	79
hsa-miR-5703	78.99
hsa-miR-181a-5p	78.86
hsa-miR-302d-3p	78.86
hsa-miR-181d	78.85
hsa-miR-3909	78.61
hsa-miR-3689a-3p	78.57
hsa-miR-488-3p	78.53
hsa-miR-23a-3p	78.4
hsa-miR-1276	78.28
hsa-miR-4477b	78.15
hsa-miR-181b-5p	78.14
hsa-miR-154-3p	78.1
hsa-miR-587	77.56
hsa-miR-4768-3p	77.53
hsa-miR-600	77.39
hsa-miR-3184-5p	77.3
hsa-miR-4262	77.25
hsa-miR-101-3p	77.18
hsa-miR-371a-5p	77.14
hsa-miR-4468	76.85
hsa-miR-4650-3p	76.79
hsa-miR-23c	76.68
hsa-miR-15a-5p	76.65
hsa-miR-676-5p	76.62
hsa-miR-506-5p	76.57
hsa-miR-5699	76.44
hsa-miR-577	76.31
hsa-miR-4307	76.17
hsa-miR-15b-5p	76.04
hsa-miR-20a-5p	75.97
hsa-miR-302c-3p	75.94
hsa-miR-588	75.92
hsa-miR-938	75.83
hsa-miR-3159	75.76
hsa-miR-30b-3p	75.73
hsa-miR-3926	75.68
hsa-miR-526b-3p	75.66
hsa-miR-4677-5p	75.5
hsa-miR-4649-3p	75.49
hsa-miR-3977	75.19

hsa-miR-548c-3p	75.14
hsa-miR-4793-3p	75.07
hsa-miR-4644	75.05
hsa-miR-3152-5p	74.99
hsa-miR-106b-5p	74.93
hsa-miR-3944-5p	74.91
hsa-miR-188-5p	74.89
hsa-miR-302e	74.81
hsa-miR-5700	74.8
hsa-miR-5582-5p	74.69
hsa-miR-1182	74.4
hsa-miR-4496	74.33
hsa-miR-452-5p	74.19
hsa-miR-4448	73.84
hsa-miR-4762-5p	73.76
hsa-miR-195-5p	73.75
hsa-miR-30e-3p	73.66
hsa-miR-30a-3p	73.56
hsa-miR-4418	73.55
hsa-miR-5586-3p	73.1
hsa-miR-3529-3p	72.96
hsa-miR-5582-3p	72.93
hsa-miR-15a-3p	72.9
hsa-miR-4717-3p	72.9
hsa-miR-4679	72.78
hsa-miR-499a-5p	72.77
hsa-miR-1243	72.69
hsa-miR-30d-3p	72.64
hsa-miR-4756-3p	72.49
hsa-miR-4288	72.47
hsa-miR-185-5p	72.42
hsa-miR-875-3p	72.38
hsa-miR-582-5p	72.28
hsa-miR-4688	72.26
hsa-miR-4306	72.21
hsa-miR-18a-5p	72.16
hsa-miR-18b-5p	71.99
hsa-miR-448	71.99
hsa-miR-623	71.93
hsa-miR-450b-3p	71.81
hsa-miR-3690	71.74
hsa-miR-548aq-5p	71.52
hsa-miR-3680-3p	71.46
hsa-miR-548av-5p	71.32
hsa-miR-548k	71.32
hsa-miR-487a	71.29
hsa-miR-125b-2-3p	71.24

hsa-miR-3672	71.1
hsa-miR-3936	70.9
hsa-miR-608	70.83
hsa-miR-580	70.78
hsa-miR-5000-5p	70.75
hsa-miR-592	70.52
hsa-miR-5583-3p	70.5
hsa-miR-30d-5p	70.43
hsa-miR-4651	70.04
hsa-miR-624-3p	69.87
hsa-miR-491-3p	69.71
hsa-let-7a-2-3p	69.66
hsa-miR-550a-3p	69.41
hsa-miR-3685	69.34
hsa-miR-5584-5p	69.27
hsa-miR-485-3p	68.96
hsa-miR-632	68.79
hsa-miR-4254	68.72
hsa-miR-497-3p	68.62
hsa-miR-922	68.61
hsa-miR-30b-5p	68.5
hsa-miR-3199	68.4
hsa-miR-4701-5p	68.34
hsa-miR-122-3p	68.34
hsa-miR-195-3p	68.28
hsa-miR-4801	68.25
hsa-miR-3182	68.09
hsa-miR-30e-5p	67.87
hsa-miR-30a-5p	67.81
hsa-miR-581	67.79
hsa-miR-617	67.71
hsa-miR-3173-3p	67.48
hsa-miR-2117	67.45
hsa-miR-5192	67.37
hsa-miR-4676-3p	67.33
hsa-miR-3138	67.06
hsa-miR-30c-5p	67
hsa-miR-548s	66.77
hsa-miR-16-5p	66.72
hsa-miR-548ag	66.62
hsa-miR-4744	66.34
hsa-miR-4536-5p	66.19
hsa-miR-4273	66.12
hsa-miR-548ar-3p	66.07
hsa-miR-4436b-3p	65.67
hsa-miR-4740-5p	65.58
hsa-miR-4520a-3p	65.5

hsa-miR-4766-3p	65.43
hsa-miR-4510	65.34
hsa-miR-451b	65.28
hsa-miR-3188	65.14
hsa-miR-3140-3p	65.09
hsa-miR-4445-5p	64.77
hsa-miR-4766-5p	64.77
hsa-miR-489	64.61
hsa-miR-16-2-3p	64.57
hsa-miR-1253	64.53
hsa-miR-4726-5p	64.5
hsa-miR-4457	64.19
hsa-miR-4709-5p	64.16
hsa-miR-4690-5p	63.95
hsa-miR-942	63.69
hsa-miR-1290	63.69
hsa-miR-4716-5p	63.57
hsa-miR-4433-3p	63.47
hsa-miR-877-3p	63.42
hsa-miR-335-5p	63.31
hsa-let-7g-3p	63.28
hsa-miR-3691-3p	63.05
hsa-miR-548ai	62.85
hsa-miR-570-5p	62.85
hsa-miR-3164	62.59
hsa-miR-541-5p	62.51
hsa-miR-4999-5p	61.98
hsa-miR-665	61.8
hsa-miR-183-3p	61.49
hsa-miR-4659a-5p	61.22
hsa-miR-3607-3p	61.21
hsa-miR-708-3p	61.2
hsa-miR-651	61.09
hsa-miR-4668-5p	60.92
hsa-miR-924	60.9
hsa-miR-4487	60.74
hsa-miR-590-3p	60.57
hsa-miR-4521	60.56
hsa-miR-548d-5p	60.56
hsa-miR-5008-3p	60.51
hsa-miR-4645-5p	60.49
hsa-miR-4494	60.47
hsa-miR-374a-3p	60.44
hsa-miR-548ab	60.34
hsa-miR-4251	60.25
hsa-miR-4505	60.13
hsa-miR-548y	60.13

hsa-miR-338-3p	60.02
hsa-miR-5690	59.96
hsa-miR-200c-5p	59.95
hsa-miR-4703-5p	59.79
hsa-miR-103a-3p	59.71
hsa-miR-1257	59.6
hsa-miR-548b-5p	59.55
hsa-miR-548w	59.48
hsa-miR-4275	59.12
hsa-miR-4758-5p	59.06
hsa-miR-34b-5p	59.04
hsa-miR-549	58.91
hsa-miR-5688	58.84
hsa-miR-4769-3p	58.81
hsa-miR-4456	58.62
hsa-miR-4323	58.57
hsa-miR-302d-5p	58.56
hsa-miR-92a-1-5p	58.42
hsa-miR-1261	58.41
hsa-miR-4719	58.35
hsa-miR-4685-5p	58.3
hsa-miR-498	58.04
hsa-miR-3130-3p	57.99
hsa-miR-548i	57.98
hsa-miR-1252	57.78
hsa-miR-4264	57.71
hsa-miR-5004-5p	57.7
hsa-miR-4761-3p	57.68
hsa-miR-4419a	57.53
hsa-miR-599	57.27
hsa-miR-4701-3p	57.01
hsa-miR-1915-3p	57.01
hsa-miR-5587-5p	56.94
hsa-miR-203	56.93
hsa-miR-548am-5p	56.9
hsa-miR-548c-5p	56.9
hsa-miR-548o-5p	56.9
hsa-miR-449a	56.9
hsa-miR-5580-3p	56.76
hsa-miR-3145-5p	56.64
hsa-miR-548au-5p	56.54
hsa-miR-548h-5p	56.44
hsa-miR-548ar-5p	56.32
hsa-miR-4704-5p	56.24
hsa-miR-4720-5p	56.11
hsa-miR-4455	55.82
hsa-miR-3147	55.8

hsa-miR-302b-5p	55.49
hsa-miR-1827	55.25
hsa-miR-548as-5p	55.21
hsa-miR-3127-3p	55.19
hsa-miR-1245a	55.11
hsa-miR-3679-5p	55.08
hsa-miR-548a-5p	54.91
hsa-miR-1912	54.91
hsa-miR-548ak	54.84
hsa-miR-3168	54.76
hsa-miR-1285-5p	54.75
hsa-miR-4704-3p	54.61
hsa-miR-4640-3p	54.58
hsa-miR-34a-5p	54.42
hsa-miR-3174	54.41
hsa-miR-139-5p	54.39
hsa-miR-548n	54.33
hsa-miR-105-5p	54.31
hsa-miR-5010-3p	54.25
hsa-miR-19b-3p	54.2
hsa-miR-519e-3p	54.1
hsa-miR-561-5p	54.04
hsa-miR-4670-3p	54.02
hsa-miR-3189-3p	53.96
hsa-miR-5588-3p	53.9
hsa-miR-548j	53.63
hsa-miR-19a-3p	53.62
hsa-miR-873-5p	53.44
hsa-miR-548ap-5p	53.42
hsa-miR-675-3p	53.1
hsa-miR-4429	53.08
hsa-miR-320d	53.06
hsa-miR-107	53.03
hsa-miR-4280	52.9
hsa-miR-548g-3p	52.87
hsa-miR-323a-5p	52.87
hsa-miR-3928	52.84
hsa-miR-3665	52.72
hsa-miR-4423-3p	52.7
hsa-miR-1272	52.69
hsa-miR-1262	52.68
hsa-miR-4324	52.64
hsa-miR-609	52.61
hsa-miR-4474-3p	52.54
hsa-miR-129-2-3p	52.19
hsa-miR-544a	52.18
hsa-miR-555	52.1

hsa-miR-129-1-3p	52.04
hsa-miR-1304-3p	52.01
hsa-miR-33b-3p	52.01
hsa-miR-216a	51.94
hsa-miR-515-5p	51.94
hsa-miR-5186	51.81
hsa-miR-2682-5p	51.65
hsa-miR-1185-5p	51.61
hsa-miR-181a-2-3p	51.59
hsa-miR-92b-3p	51.56
hsa-miR-586	51.54
hsa-miR-512-5p	51.5
hsa-miR-320a	51.32
hsa-miR-320b	51.32
hsa-miR-222-5p	51.32
hsa-miR-644b-5p	51.28
hsa-miR-4265	51.26
hsa-miR-3714	51.2
hsa-miR-561-3p	50.99
hsa-miR-515-3p	50.92
hsa-miR-4677-3p	50.91
hsa-miR-4707-3p	50.9
hsa-miR-4435	50.87
hsa-miR-4652-3p	50.86
hsa-miR-320c	50.72
hsa-miR-525-5p	50.7
hsa-miR-299-5p	50.61
hsa-miR-3927	50.4
hsa-miR-4728-3p	50.03
hsa-miR-138-2-3p	50
hsa-miR-22-5p	50
hsa-miR-337-3p	50
hsa-miR-411-3p	50
